# Discovery of New Boswellic Acid Hybrid 1*H*-1,2,3-Triazoles for Diabetic Management: In Vitro and In Silico Studies

**DOI:** 10.3390/ph16020229

**Published:** 2023-02-02

**Authors:** Najeeb Ur Rehman, Saeed Ullah, Tanveer Alam, Sobia Ahsan Halim, Tapan Kumar Mohanta, Ajmal Khan, Muhammad U. Anwar, René Csuk, Satya Kumar Avula, Ahmed Al-Harrasi

**Affiliations:** 1 Natural & Medical Sciences Research Center, University of Nizwa, Nizwa 616, Oman; 2 Organic Chemistry, Martin-Luther-University Halle-Wittenberg, Kurt-Mothes-Str. 2, D-06120 Halle (Saale), Germany

**Keywords:** 3-*O*-acetyl-11-keto-β-boswellic acid (AKBA), 11-keto-β-boswellic acid (KBA), 1*H*-1,2,3-triazole hybrids, click chemistry, α-glucosidase inhibitors, molecular docking studies

## Abstract

A series of 24 new 1*H*-1,2,3-triazole hybrids of 3-*O*-acetyl-11-keto-β-boswellic acid (β-AKBA (**1**)) and 11-keto-β-boswellic acid (β-KBA (**2**)) was designed and synthesized by employing “click” chemistry in a highly efficient manner. The 1,3-dipolar cycloaddition reaction between β-AKBA-propargyl ester intermediate **3** or β-KBA-propargyl ester intermediate **4** with substituted aromatic azides **5a–5k** in the presence of copper iodide (CuI) and Hünig’s base furnished the desired products—1*H*-1,2,3-triazole hybrids of β-AKBA (**6a–6k**) and β-KBA (**7a–7k**)—in high yields. All new synthesized compounds were characterized by ^1^H-, ^13^C-NMR spectroscopy, and HR-ESI-MS spectrometry. Furthermore, their α-glucosidase-inhibitory activity was evaluated in vitro. Interestingly, the results obtained from the α-glucosidase-inhibitory assay revealed that all the synthesized derivatives are highly potent inhibitors, with IC_50_ values ranging from 0.22 to 5.32 µM. Among all the compounds, **6f**, **7h**, **6j**, **6h**, **6g**, **6c**, **6k**, **7g**, and **7k** exhibited exceptional inhibitory potency and were found to be several times more potent than the parent compounds **1** and **2**, as well as standard acarbose. Kinetic studies of compounds **6g** and **7h** exhibited competitive and mixed types of inhibition, with ki values of 0.84 ± 0.007 and 1.18 ± 0.0012 µM, respectively. Molecular docking was carried out to investigate the binding modes of these compounds with α-glucosidase. The molecular docking interactions indicated that that all compounds are well fitted in the active site of α-glucosidase, where His280, Gln279, Asp215, His351, Arg442, and Arg315 mainly stabilize the binding of these compounds. The current study demonstrates the usefulness of incorporating a 1*H*-1,2,3-triazole moiety into the medicinally fascinating boswellic acids skeleton.

## 1. Introduction

Frankincense (oleo-gum exudates of *Boswellia* ssp.) is the most precious resin religiously, economically, socially, and medicinally. The distribution of the *Boswellia* trees ranges from South Arabia to India to the horn of Africa [1]. Frankincense exists in traditional Chinese medicine to treat ulcers, rheumatoid arthritis, dysmenorrhea, swelling, osteoarthritis, pain from injuries, and amenorrhea [2,3,4]. *B. sacra* is endemic to Oman, and the resin (olibanum or frankincense) of *B. serrata* is known for its superb anti-inflammatory and pro-apoptotic activity [5,6]. Frankincense and triterpenoids from *B. sacra* and other species of the same genus have been used in traditional medicine since ancient times to treat obesity and lipid disorders [4,7].

**Figure 1:**
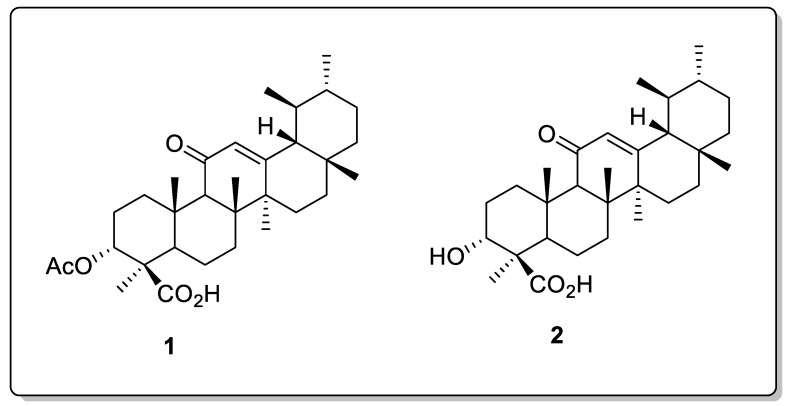
Chemical structures of β-AKBA (**1**) and β-KBA (**2**).

The oleo-gum resin of *Boswellia* contains an abundant quantity of pentacyclic triterpenoid acids and boswellic acids (BAs), and is reported to have multiple beneficial bioactivities: particularly, anticancer [8], hepatoprotective [9,10,11], anti-inflammatory [5], 5-lipoxygenase [12], neuroprotective [13], apoptosis [14], antiproliferative [15], antidepressant [1], anti-arthritic, and anti-tumor [7,9] bioactivities. Due to their known biological activities, boswellic acids are of special interest [16,17,18]. Due to the superb anti-inflammatory activity of β-AKBA (**1**) and β-KBA (**2**) (Figure 1), we selected them as precursors in the current study [15,19].

In addition, the importance of 1*H*-1,2,3-triazole molecules in pharmaceuticals and agrochemicals has increased [20]. Triazole compounds play a key role in organic chemistry due to their multiple and broad range of applications in bio-medicinal, biochemical, and material sciences [21]. The interest of the compounds containing the triazole moiety underwent a substantial growth over the past few decades. Furthermore, heterocycles containing 1,2,3-triazole scaffolds are known to have biological activities including anti-microbial [22], anti-bacterial [23], anti-viral [24], anti-HIV [25], anti-inflammatory [26], and anti-cancer [27] activities. Our group have recently reported the synthetic modifications of the β-AKBA and β-ABA hybrid triazole moieties as potent carbonic anhydrase-II [28].

In the drug discovery process, the incorporation of an active pharmacophoric moiety into a core bioactive natural product is a key step in exploring a wide range of new chemical entities with enhanced biological activities. With this objective in our mind, and in continuation of our research interest involving the chemical transformation of compounds **1** and **2** through the molecular hybridization approach, we herein describe the synthesis of novel β-AKBA (**1**)- and β-KBA (**2**)-based 1*H*-1,2,3-triazole hybrids and molecular docking studies with respect to their α-glucosidase activities.

Hyperglycemia is one of the most serious diabetes outcomes, leading to long-term health issues such as cardiovascular diseases, metastatic cancer, retinopathy, and nephropathy [29]. According to a worldwide projection, factors such as population growth, aging, urbanization, and lifestyle may cause the global prevalence rate of diabetes to rise to 54 percent by 2030 [30]. α-glucosidase is a hydrolase enzyme which is present on the brush border of the small intestine and catalyzes carbohydrates digestion into glucose molecules, which are then absorbed into blood. The use of α-glucosidase inhibitors has been shown to be a reliable approach for drug development and to overcome hyperglycemia. As a result, α-glucosidase inhibitors (AGIs) have received a lot of interest due to their therapeutic usage in the treatment of nocturnal hypoglycemia [31]. A fundamental treatment method used to overcome hyperglycemia is to reduce carbohydrate digestion and absorption, which provides an insight into the important role of α-glucosidase. As a result, novel AGIs with fewer side effects are urgently needed, in contrast to the currently existing clinical AGIs, such as acarbose, voglibose, and miglitol [32,33]. Recently, it was reported in the literature that both 1*H*-1,2,3-triazole and BAs are active pharmacophores of α-glucosidase inhibitors [34,35,36]. In the current study, we conjugated the 1*H*-1,2,3-triazole with BAs to discover their hybrid behavior against α-glucosidase.

Keeping in mind the importance of boswellic acids, triazole moieties, and AGIs, we planned to synthesize their hybrids between BAs and triazoles, and screened them against the α-glucosidase enzyme. Furthermore, this is the first report, to the best of authors’ knowledge, on the synthetic hybrids of 1*H*-1,2,3-triazole with β-AKBA (**1**) and β-KBA (**2**). In addition, here, we report the crystal structure of compound **4** for the first time. Additionally, some structure–activity relationships, molecular docking, and kinetic studies of the active analogs are also discussed.

## 2. Results and Discussion

### 2.1. Chemistry

**Scheme 1:**
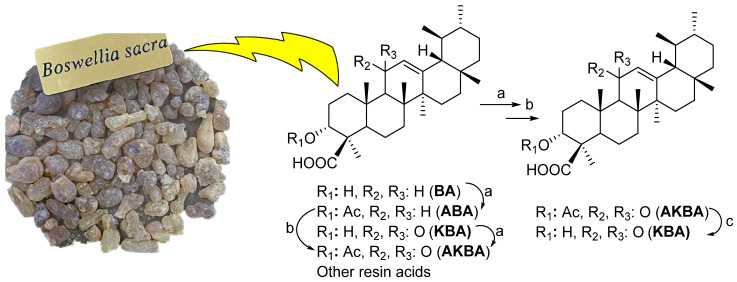
Large-scale production of β-AKBA (**1**) and β-KBA (**2**). Reagents and conditions: (a) Ac_2_O/Py/DMAP, CH_2_Cl_2_, room temperature, 6 h; (b) NBS/CaCO_3_/H_2_O/hν, dioxane, room temperature, 10 h; (c) 0.5 N KOH in ^i^PrOH, reflux, 10 h.

The compounds β-AKBA (**1**) (900 mg) and β-KBA (**2**) (840 mg) were employed as starting materials, which were obtained as white crystals from the oleo-gum resin of *Boswellia sacra* [37]. As the concentration of β-AKBA is low in the resin of *B. sacra* compared to other boswellic acids (BA, KBA, and ABA), in order to prepare (**1**) and (**2**) in large quantities, we transformed a cluster of BAs (20 gm) into β-AKBA and purified them through column chromatography (70–230 mesh) using the increasing polarity of a 20–30% *n*-hexane/EtOAc solvent system as a mobile phase to obtain pure β-AKBA (3.2 gm). In the second step, β-AKBA (1.6 gm) was deacetylated and purified through column chromatography (CC) using 30–40% *n*-hexane/EtOAc as a mobile phase to afford pure β-KBA (1.4 gm) (Scheme 1). The structures of the compounds were confirmed through 1D (^1^H- and ^13^C) NMR spectroscopy and mass spectrometry (HR-ESI-MS) [7,15,37].

**Scheme 2:**
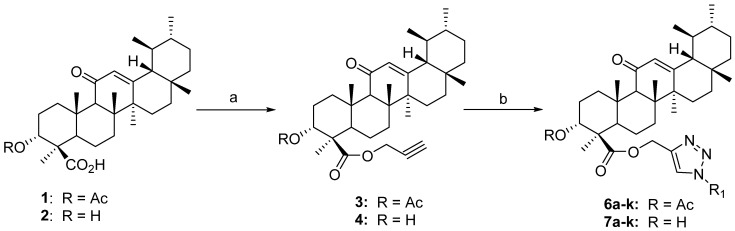
Reagents and conditions: (a) propargyl bromide, K_2_CO_3_, DMF, room temperature, 18 h, 94–96%; (b) R_1_-N_3_ (different substituted aromatic azides, **5a–k**), CuI, Et_3_N, CH_3_CN, room temperature, 3 h, **6a–6k** (87–96%), **7a–7k** (86–96%).

The synthetic scheme of 1*H*-1,2,3-triazole hybrids of β-AKBA (**1**) and β-KBA (**2**) is depicted in Scheme 2. In the present study, a series of new analogues of β-AKBA and β-KBA containing the 1*H*-1,2,3-triazole moiety were designed and synthesized. The desired 1*H*-1,2,3-triazole hybrids (**6a–6k** and **7a–7k**) were achieved using a two-step protocol with propargylation followed by Huisgen’s 1,3-dipolar cycloaddition reaction. Thereby, in the initial step, compounds **1** and **2** were treated in DMF with propargyl bromide in the presence of potassium carbonate at room temperature to afford the β-AKBA-propargyl ester intermediate **3** (yield 96%) and β-KBA-propargyl ester intermediate **4** [38,39,40,41].

Step 2 was carried out using “click” chemistry. In step 2, a 1,3-dipolar cycloaddition reaction was conducted between β-AKBA-propargyl ester intermediate **3** and aromatic azides **5a–5k**, in the presence of copper iodide (CuI) and Hünig’s base in MeCN, to obtain the desired products—1*H*-1,2,3-triazole hybrids of β-AKBA derivatives **6a–6k**—in very good yields (87–96%) [40,41]. Under similar conditions, the 1*H*-1,2,3-triazole hybrids of β-KBA derivatives **7a–7k** in excellent yields of 86–96% were obtained from β-KBA-propargyl ester intermediate **4** (Table 1).

The ^1^H NMR spectrum of compound **3** showed characteristic signals at δ 2.46 and 4.71 corresponding to acetylenic (-C≡CH) and oxymethelene (-OCH_2_-) protons, respectively. The ^13^C NMR spectrum of compound **3** confirmed the above observations by exhibiting signals at δ 174.7, 76.8, 75.0, and 51.8, corresponding to the ester (-COOR), acetylene (C≡CH) and oxymethelene (-OCH_2_-) groups. Further confirmation of compound **3** was conducted by HRMS (ESI), which showed protonated molecular ion at (*m*/*z*) 551.3713 [M+H]^+^.

The ^1^H NMR spectrum of compound **4** showed prominent signals at δ 2.48 and 4.66, corresponding to acetylenic (-C≡CH) and oxymethelene (-OCH_2_-) protons, respectively. The ^13^C NMR spectrum of compound **4** confirmed the above observations by exhibiting signals at δ 167.1, 77.4, 74.7, and 51.5, corresponding to the ester (-COOR), acetylene (-C≡CH), and oxymethelene (-OCH_2_-) functionalities. Further confirmation of compound **4** was achieved by HRMS (ESI), which showed protonated molecular ion at (*m*/*z*) 509.3609 [M+H]^+^.

**Figure 2:**
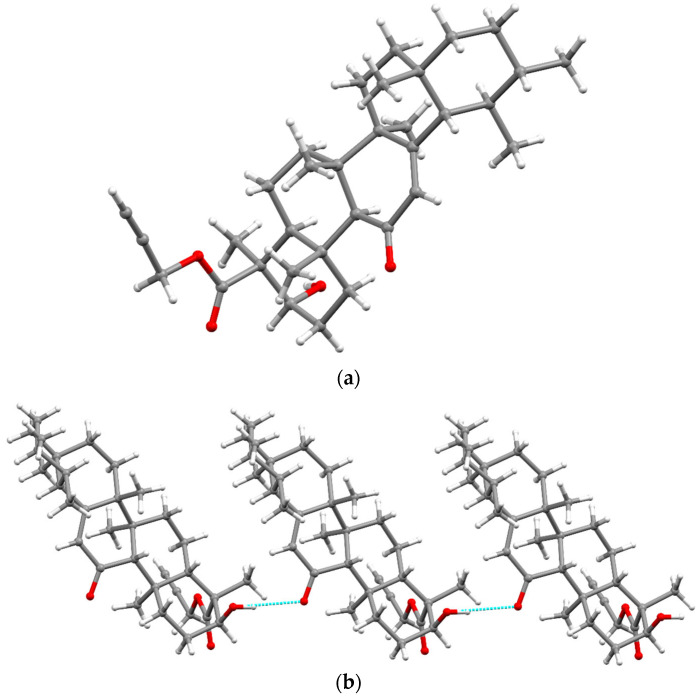
X-ray crystal structure of compound **4** (**a**). Hydrogen bonding in **4**; H-bonds are shown in blue (**b**).

The structure of compound **4** was unambiguously confirmed using X-ray crystallography. The molecular structure of compound **4** is illustrated in Figure 2a, while the crystal data are depicted in Table S2 (Supporting Information). Compound **4** crystallizes in the orthorhombic space group P2_1_P2_1_P2_1_. All cyclohexane rings of the pentacyclic backbone adopt chair conformations, with the exception of a ring containing sp^2^-hybdridized C-atoms (C12–C13). C9 and C11–C14 form an approximately planar region, with C8 out of the plane, resulting in a sofa or a half chair conformation of the ring. The double-bond character of C12–C13 was confirmed by their distance of 1.343 (4) Å. The bond distance between C3–O3 was 1.431 Å, which confirms their single-bond character. The bond distance between C11–O4 was 1.226 (3) Å, which suggests a double-bond character. The C32–C33 bond distance in the propargilate fragment was 1.164 (3), which confirms the triple-bond character. Compound **4** is stabilized by intramolecular hydrogen bonding. The O–H group forms a hydrogen bond with the oxygen (O4) of the neighboring molecule (*d*_O3–H1⋯O4_ = 2.08 (3) Å, *Ɵ*_O3–H1⋯O4_ = 172 (4)^o^)**,** resulting in a hydrogen-bonded one-dimensional chain along *c*-axis (Figure 2b).

The structures of all the synthesized compounds (**6a–6k** and **7a–7k**) were characterized by spectral data analysis (^1^H and ^13^C NMR, and HRMS). ^19^F NMR spectroscopy was used for the compounds containing fluorine. The formation of 1*H*-1,2,3-triazole hybrids of β-AKBA (**6a–6k**) and β-KBA (**7a–7k**) derivatives was confirmed by the presence of a characteristic singlet observed in their corresponding ^1^H NMR spectra at δ 7.60–8.30, which can be attributed to the H-5′ proton of the triazole ring. Further, the ^13^C NMR spectra of the synthesized hybrids (**6a–6k** and **7a–7k**) determined the diagnostic triazole ring carbon signals between δ 123~125 and 142~144. The structures of the synthesized 1*H*-1,2,3-triazole hybrids (**6a–6k** and **7a–7k**) were further supported by HRMS.

### 2.2. α-Glucosidase Activity

The synthesized derivatives of boswellic acids were evaluated for their α-glucosidase-inhibitory potential. Consequently, all the compounds exhibited significant inhibitory activities in the range of 0.22–5.32 µM, at a level even superior to the standard inhibitor acarbose (IC_50_ = 942 ± 0.74 µM). Compounds **3** and **4**, containing the same propargylic groups, displayed slight variations in their α-glucosidase-inhibitory activity after the addition of the triazole moiety with different substituents. Compound **3** with the propargylic group displayed a potent inhibitory activity, with an IC_50_ value of 4.72 ± 0.52 µM, while the addition of the triazole moiety in **6a** was favorable in order to enhance the inhibitory activity (IC_50_ = 2.02 ± 0.031 µM). However, the substitution of the *ortho*-methyl group in **6b** slightly decreased the inhibitory activity of **6b** (IC_50_ = 2.25 ± 0.10 µM), as compared to **6a** with no substitution. However, the substitution of the methoxy group at the *ortho-*position in **6c** (IC_50_ = 1.23 µM) and **6e** (IC_50_ = 2.01 µM) showed only a modest impact on the inhibition, as compared to substitution at the *para-*position. The substitution of the tri-fluoro methyl group at the *meta-*position of **6f** (IC_50_ = 0.22 µM) remarkably increased the inhibitory activity, more so than substitutions at the *ortho-*and *para-*positions of compounds **6d** (IC_50_ = 2.37 µM) and **6k** (IC_50_ = 1.26 µM). Moreover, a minute variation was observed in the inhibitory potential by the substitution of the bromo phenyl group in compounds **6g** and **6h**, with IC_50_ values of 0.78 and 0.69 µM, respectively. In compound **6j**, the addition of a highly electronegative fluorine atom at the *meta-*position displayed significant inhibitory potential, with an IC_50_ value of 0.58 µM, as compared to the replacement of the chlorine atom at the same position in **6i** (IC_50_ = 3.06 µM) (Table 2).

Compound **4** (IC_50_ value of 5.32 ± 0.15 µM) with the propargylic group displayed the least α-glucosidase-inhibitory potential among all the compounds. However, the addition of triazole group, along with various substitutions at different positions, improved the inhibitory potential of compound **4** derivatives. The phenyl-substituted derivative of **4** (**7a**) exhibited a higher level of in vitro inhibitory activity (IC_50_ = 2.31 µM) than compound **4**. Similarly, **7b** with the methyl substitution at the *meta-*position of the phenyl ring also displayed a potent inhibitory activity (IC_50_ = 2.11 µM), while the substitution of the tri-fluoro methyl group at the *meta-*and *ortho-*positions of **7d** and **7f**, respectively, produced only a slight difference in the inhibitory potential, with IC_50_ values of 2.62 and 2.52 µM, respectively, in contrast to the addition of the same group at the *para-*position in **7k**, which displayed an increase in the inhibitory activity (IC_50_ = 1.39 µM). Compound **7c** with a *meta-*methoxy-substituent displayed a slight increase in inhibitory activity (IC_50_ = 2.18 µM), as compared to **7e** (IC_50_ = 2.37 µM) with a similar substituent at the *para-*position. Compound **7h** with *para-*bromo substitution displayed more potent inhibitory activity (IC_50_ = 0.43 µM) than **7g** (IC_50_ = 1.34 µM) with the same substitution at the *ortho-*position. In contrast, compound **7j** with *para-*fluoro substitution exhibited more potent inhibitory activity (IC_50_ = 2.64 µM) than **7i** with a chloro-substituent at the same *para-*position (IC_50_ = 3.18 µM). Comparing both series (**6a–6k** and **7a–7k**), four compounds of AKBA derivatives (**6c**, **6f**, **6g**, and **6j**) displayed a higher level of inhibition than KBA derivatives (**7c**, **7f**, **7g**, and **7j**), compared to the standard, which showed the importance of the acetate group over hydroxyl, while the rest of the compounds determined an almost similar inhibition. Only one compound, **7h** (KBA derivative), showed a higher level of inhibin than **6j** (AKBA derivative), which could possibly be due to the substituted group attached to the triazoles moiety.

#### Kinetic Studies

**Figure 3:**
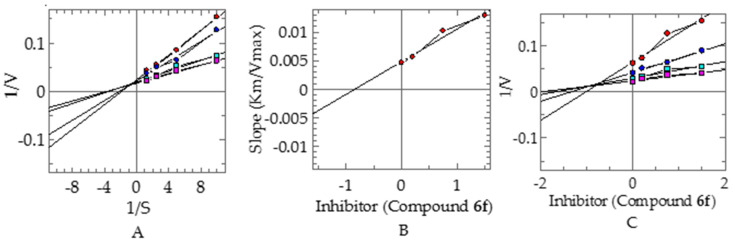
Mode of inhibition of α-glucosidase by compound **6f**: (**A**) Lineweaver–Burk plot of reciprocal of rate of reaction (velocities) vs. reciprocal of substrate p-nitro phenyl-α-D-glucopyranoside in the absence of (■), and in the presence of 1.50 (●), 0.75 µM (●), and 0.200 µM (■) of compound **6f**. (**B**) Secondary replot of Lineweaver–Burk plot between the slopes of each Lineweaver–Burk plot vs. different concentrations of compound **6f**. (**C**) Dixon plot of reciprocal of rate of reaction (velocities) vs. different concentrations of compound **6f**.

**Figure 4:**
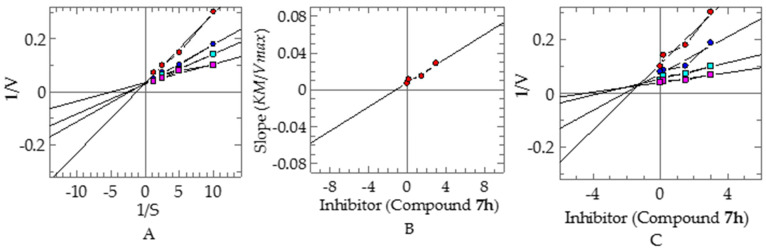
Mode of inhibition of α-glucosidase by compound **7h** (**A**) Lineweaver–Burk plot of reciprocal of rate of reaction (velocities) vs. reciprocal of substrate p-nitro phenyl-α-D-glucopyranoside in the absence of (■), and in the presence of 3.00 (●), 1.50 µM (●), and 0.1875 µM (■) of compound **7h**. (**B**) Secondary replot of Lineweaver–Burk plot between the slopes of each Lineweaver–Burk plot vs. different concentrations of compound **7h**. (**C**) Dixon plot of reciprocal of rate of reaction (velocities) vs. different concentrations of compound **7h**.

To investigate their inhibitory mechanisms, the most potent compounds **6f** and **7h** were subjected to kinetic studies. Compound **6f** exhibited a competitive type of inhibition, with a ki value of 0.84 ± 0.007 µM (Figure 3). In this type of inhibition, the inhibitor binds with the active site residue of enzyme and, therefore, *Vmax* remains the same, with an increase in the *Km*. The kinetic studies of compound **7h** revealed a mixed type of inhibition, with a ki value of 1.18 ± 0.0012 µM (Figure 4). In this type of inhibition, both inhibitors—*Vmax* and *Km*—are affected by *Vmax* decreases and *Km* increases.

### 2.3. Molecular Docking Studies

Initially, the docking protocol was validated through the re-docking of the competitive inhibitor: maltose in the active site of the enzyme (PDB code: 3A4A). The re-docking results showed that the maltose was docked at its binding site with RMSD = 1.31Å (Figure S1, Supplementary Materials) and a docking score of −5.94 kcal/mol, which indicated that the docking method was reliable to use to predict the binding modes of our compounds.

To investigate the binding mode of the compounds in the active site of α-glucosidase, we carried out a molecular docking experiment. In the docking studies, it was observed that the propargyl-linked carbonyl moiety of **3** fit deep inside the active site and formed a hydrogen bond (H-bond) with the side chain of Arg442. In contrast, the carbonyl group of **4** did not interact with the surrounding residues; instead, its -OH group formed an H-bond with the side chain of Arg442. The boswellic acid skeleton of both of the compounds fit at the entrance of the active site and blocked the access to substrate in the active site. The binding modes of compounds **3** and **4** derivatives suggest that the addition of the triazole-substituted phenyl ring dragged the boswellic acid moieties of the compounds towards the entrance, while the substituted groups fit deep inside the active site.

The most active compound, **6f**, mediated multiple H-bonds within the active site. The triazole-linked carbonyl oxygen of **6f** formed an H-bond with His280, while the acetate moiety of **6f** mediated a bidentate interaction with Arg315. Additionally, the side chain of Ser240 donated a H-bond to the carbonyl group in the boswellic acid. The binding modes of **7h** and **6j** reflect that the carbonyl and -OH moieties of **7h** mediated H-bonding with Arg315 and Ser157, respectively, whereas the acetate and the carbonyl groups of **6j** formed a bidentate interaction with Arg315 and Ser240, respectively. Similarly, the carbonyl moiety of **6h** formed an H-bond with Arg315, while its triazole ring interacted with Arg442 through the H-bond. The carbonyl groups of **6g** mediated H-bonds with His280 and Ser240. The side chains of Ser240 and Gln279 bound with the boswellic acid carbonyl oxygen and the phenyl-substituted methoxy group of **6c**, respectively, while His280 bound with the triazole-substituted oxygen of **6c** and carbonyl oxygen of **6k**. Moreover, the -OH and the triazole-substituted oxygen of **7g** formed H-bonds with the side chains of Glu411 and Gln279, respectively. The acetate moiety of **7k** and **6e** was linked with the side chain of Gln279.

Furthermore, the side chains of His351, Arg442, and Asp242 formed H-bonds with the triazole ring of **6a** and the -OH of **7b** and **7c**, respectively. Meanwhile, **6b** formed H-bonds with the side chain of Ser240 and His280 and -OH of **7a** bind with Arg442. The carbonyl oxygen and fluorine atom of **6d** formed an H-bond with the side chains of Gln279 and His351, respectively. Similarly, Ser240 provided an H-bond to the carbonyl oxygen of **7e**. The binding modes of **7f** and **7d** showed that **7f** formed H-bonds with the side chain of Gln279 and His112, while **7d** only mediated an H-bond with the side chain of Arg213. The docked view of **7j** suggested that the compound fit deep inside the active site and mediated multiple H-bonds with His351, Asp215, and Gln279. Similarly, the acetate, triazole, and carbonyl groups of **6i** formed several H-bonds with Arg442, His351, Asn350, and Arg315. The docked conformation of **7i** was found to be more surface-exposed, and formed only an H-bond with the Tyr158. Afterwards, compounds **2** and **1** exhibited the least inhibitory activities, compared to other compounds in this series.

**Figure 5:**
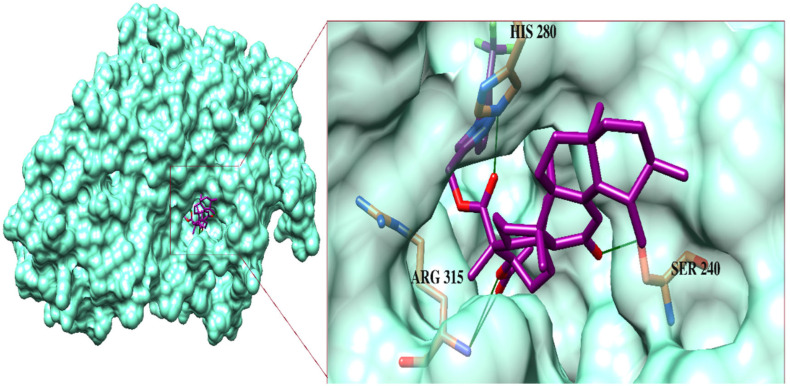
The binding mode of the most active compound, **6f** (shown in the purple stick model), in the active site of enzyme. The ligand-binding residues are presented in the orange stick model and H-bonds are shown in green lines.

The acetate and -OH groups of **2** mediated H-bonds with the side chains of Arg442 and Asp215, respectively. Meanwhile, the acetate moiety of **1** interacted with the side chains of Arg213 and His351. The binding modes of these compounds indicate that His280, Gln279, Asp215, His351, Arg442, and Arg315 are the important residues that provide H-bonds to these compounds, while Phe301 and Phe303 provide π–π interaction to most of the compound at the entrance of the active site. The binding mode of the most active hit is shown in Figure 5. The docking score of the compounds were in the range of −9.18 to −2.23 Kcal/mol. The atom-based protein–ligand interaction of each compound is tabulated in Table S1 (Supplementary Materials), along with their docking scores. The docking results support our experimental finding.

## 3. Experimental Section

### 3.1. General

All experiments were carried out in dry reaction vessels under a dry nitrogen atmosphere. All reagents were purchased from Sigma-Aldrich, Germany. Solvents were purified and dried according to the standard procedures. The high-resolution electrospray ionisation mass spectra (HR-ESI-MS) were recorded on an Agilent 6530 LC Q-TOF LC/MS (Agilent, country of origin USA/EU, made in Singapore). The ^1^H and ^13^C NMR spectra were recorded on a nuclear magnetic resonance (NMR) spectrometer (Bruker, Zürich, Switzerland) operating at 600 MHz (150 MHz for ^13^C) using the solvent peak as an internal reference (CDCl_3_, δ H: 7.26; δ C: 77.0). The ^19^F NMR spectra were recorded at 564 MHz. Data were reported in the following order: chemical shift (δ) in ppm, multiplicities, and coupling constants (*J*) in Hertz (Hz). The column chromatography was carried out using silica gel of the selected particle size of 100–200 mesh. All reactions were monitored by thin layer chromatography (TLC) using silica gel F_254_ pre-coated plates (Merck, Darmstadt, Germany). Visualization was accomplished using UV-light and I_2_ stain. The solvents for column chromatography (EtOAc, *n*-hexane) were technical grade and distilled prior to use. The organic extracts were dried over anhydrous MgSO_4_. Single crystals of **4** were mounted on a MiTeGen loop with grease and examined on a Bruker D8 Venture APEX diffractometer equipped with a Photon 100 CCD area detector at 296 (2) K using graphite-monochromated Mo-K*_α_* radiation (λ = 0.71073 Å). Data were collected using the APEX-II software [42], integrated using SAINT [43], and corrected for absorption using a multi-scan approach (SADABS) [44]. The final cell constant was determined from full least squares refinement of all observed reflections. The structure was solved using intrinsic phasing (SHELXT) [45]. All non-H atoms were in subsequent difference maps and refined anisotropically. H-atoms were added at calculated positions and refined with a riding model. H atoms on O and N atoms were in a difference map and refined with constrained O—H and N—H distances. The structure of **4** was deposited with the CCDC (CCDC deposition number = 2,151,126).

### 3.2. Preparation of Boswellic Acids (BAs) Cluster

The air-dried ground material (100 g) of *B. sacra* resin was exhaustively extracted with 100% MeOH at room temperature (three times). The extract was evaporated to yield the yellowish residue (70 g). The MeOH extract was subjected to column chromatography (CC, SiO_2_ (70–230 mesh; Merck) using *n*-hexane/EtOAc up to 50% EtOAc, and then washed by pure EtOAc) to yielded eight fractions (BSF1–8). After taking comparative TLC with BA standards, three fractions (BSF3–5, 20 gm, 20–40% *n*-hexane/EtOAc) were combined and used for the large-scale production of β-AKBA.

### 3.3. General Procedure for Synthesis of 3 and 4

Anhydrous potassium carbonate (87.0 mg, 0.63 mmol) and propargyl bromide (75.0 mg, 0.63 mmol) were added successively to a solution of β-AKBA (**1**) or β-KBA (**2**) (200.0 mg, 0.42 mmol) in dry DMF (10 mL). The reaction mixture was stirred at room temperature for 18 h until the completion of the reaction (monitored by TLC analysis). After the completion of the reaction, it was extracted with ethyl acetate (3 times). The combined organic layer was dried over anhydrous MgSO_4_ and concentrated under reduced pressure on a rotary evaporator to obtain the crude product, which was purified by column chromatography on silica gel using *n*-hexane/ethyl acetate (95:5 *v*/*v*) as an eluent to furnish the pure compounds **3** (96%) and **4** (94%), respectively.

#### 3.3.1. (Prop-2-yne-1-yl) 3α-acetyloxy-11-oxo-urs-12-en-24-oate (**3**)

White solid; yield = 96%; ^1^H NMR (600 MHz, chloroform-*d*) δ 5.51 (s, 1H), 5.30 (s, 1H), 4.70–4.62 (m, 2H), 2.50 (dt, *J* = 13.5, 3.5 Hz, 1H), 2.43 (d, *J* = 2.4 Hz, 1H), 2.38 (s, 1H), 2.18 (ddd, *J* = 14.6, 11.6, 3.3 Hz, 1H), 2.06 (s, 3H), 1.87 (dd, *J* = 13.5, 4.5 Hz, 2H), 1.81 (d, *J* = 7.6 Hz, 3H), 1.74–1.71 (m, 1H), 1.67–1.62 (m, 1H), 1.60–1.56 (m, 1H), 1.51 (d, *J* = 11.1 Hz, 1H), 1.47–1.41 (m, 3H), 1.39–1.35 (m, 2H), 1.31 (s, 3H), 1.23–1.19 (m, 2H), 1.17 (s, 3H), 1.15 (s, 3H), 1.04 (s, 3H), 1.02–0.98 (m, 1H), 0.91 (s, 3H), 0.84 (d, *J* = 14.1 Hz, 1H), 0.79 (s, 3H), 0.77 (d, *J* = 6.4 Hz, 3H); ^13^C NMR (150 MHz, CDCl_3_) δ 199.2, 174.7, 170.1, 164.9, 130.4, 75.0, 73.0, 60.2, 59.0, 51.8, 50.4, 46.7, 45.0, 43.7, 40.9, 39.3, 37.2, 34.5, 33.9, 32.8, 30.8, 28.8, 27.5, 27.2, 23.7, 23.5, 21.3, 21.1, 20.5, 18.7, 18.2, 17.4, 13.4; HRMS (ESI^+^): found [M+3H]^+^: 551.37139 (calculated for C_36_H_55_O_4_, 551.41004).

#### 3.3.2. (Prop-2-yne-1-yl) 3α-hydroxy-11-oxo-urs-12-en-24-oate (**4**)

Light green-colour crystals; yield = 94%; ^1^H NMR (600 MHz, chloroform-*d*) δ 5.52 (s, 1H), 4.66 (t, *J* = 2.2 Hz, 2H), 4.09 (s, 1H), 2.48 (dd, *J* = 13.6, 3.6 Hz, 1H), 2.45–2.38 (m, 2H), 2.26 (t, *J* = 14.9 Hz, 1H), 2.07 (td, *J* = 13.7, 4.8 Hz, 1H), 1.87 (t, *J* = 13.2 Hz, 2H), 1.71 (d, *J* = 14.3 Hz, 1H), 1.65 (td, *J* = 13.0, 3.9 Hz, 1H), 1.52 (s, 3H), 1.46 (d, *J* = 12.7 Hz, 2H), 1.44–1.37 (m, 2H), 1.33 (d, *J* = 3.3 Hz, 1H), 1.31 (d, *J* = 3.1 Hz, 1H), 1.29 (s, 6H), 1.27–1.21 (m, 2H), 1.21–1.17 (m, 1H), 1.16 (s, 3H), 1.05 (s, 3H), 1.00 (dd, *J* = 12.7, 8.2 Hz, 1H), 0.92 (s, 3H), 0.90–0.83 (m, 1H), 0.80 (s, 3H), 0.77 (d, *J* = 6.4 Hz, 3H); ^13^C NMR (150 MHz, CDCl_3_) δ 199.4, 175.8, 164.9, 156.1, 130.5, 77.4, 74.7, 70.5, 60.3, 59.0, 51.5, 48.8, 47.5, 45.0, 43.8, 40.9, 39.2, 37.4, 33.9, 33.8, 32.8, 30.9, 28.8, 27.5, 27.1, 26.2, 24.0, 21.1, 20.5, 18.8, 18.2, 17.4, 13.3; HRMS (ESI^+^): found [M+H]^+^: 509.36097 (calculated for C_33_H_49_O_4_ required 509.36309).

### 3.4. General Procedure for Synthesis of 1H-1,2,3-Triazol Hybrids of 3-Acetyl-11-keto-β-boswellic Acid (**6a–6k**) and 11-Keto-β-boswellic Acid (**7a–7k**) Derivatives

CuI (20 mg, 0.104 mmol) and Et_3_N (0.022 mL, 0.156 mmol) at room temperature were added to a solution of β-AKBA-propargyl ester intermediate **3** (30 mg, 0.052 mmol) or β-KBA-propargyl ester intermediate **4** (30 mg, 0.051 mmol) and substituted aromatic azides **5a–k** (0.062 mmol, 1.2 eq) in acetonitrile (10 mL), and the mixture was stirred for 3 h. The reaction mixture was diluted with EtOAc (30 mL), 20 mL of aqueous NH_4_Cl was added, and the aqueous layer was extracted with EtOAc (3 × 30 mL). Then, the combined organic layer was washed with brine (1 × 20 mL), dried over anhydrous MgSO_4_, filtered, and the filtrate was concentrated in vacuo. The crude residue was purified by flash column chromatography (silica gel, *n*-hexane/EtOAc, 85:15) to yield **6a–6k** (87–96%) or **7a–7k** (86–96%), respectively.

#### 3.4.1. (4-Phenyl-1*H*-1,2,3-triazol-1-yl) Methyl 3α-acetyloxy-11-oxo-urs-12-en-24-oate (**6a**)

Gummy white solid; yield = 90%; ^1^H NMR (600 MHz, chloroform-d) δ 8.01 (s, 1H), 7.69–7.64 (m, 2H), 7.49 (t, *J* = 7.7 Hz, 2H), 7.41 (t, *J* = 7.3 Hz, 1H), 5.48 (s, 1H), 5.29 (s, 3H), 2.46 (dt, *J* = 13.5, 3.5 Hz, 1H), 2.35 (s, 1H), 2.15 (t, *J* = 14.8 Hz, 1H), 2.04 (s, 3H), 2.00 (s, 1H), 1.87–1.80 (m, 3H), 1.80–1.74 (m, 1H), 1.70 (dd, *J* = 14.2, 3.1 Hz, 1H), 1.64–1.54 (m, 2H), 1.48 (s, 1H), 1.42 (dd, *J* = 12.8, 9.8 Hz, 2H), 1.35 (d, *J* = 11.5 Hz, 2H), 1.29 (s, 3H), 1.26 (d, *J* = 10.1 Hz, 1H), 1.22 (t, *J* = 7.1 Hz, 2H), 1.18 (d, *J* = 4.3 Hz, 1H), 1.14 (s, 3H), 1.09 (s, 3H), 0.99–0.96 (m, 1H), 0.90 (s, 3H), 0.86 (s, 3H), 0.77 (s, 3H), 0.75 (d, *J* = 6.3 Hz, 3H); ^13^C NMR (150 MHz, CDCl_3_) δ 199.2, 175.6, 170.1, 165.0, 142.9, 136.8, 130.4, 129.8, 128.9, 122.3, 120.6, 73.1, 60.2, 59.0, 57.4, 50.4, 46.7, 45.0, 43.7, 40.8, 39.2, 37.2, 34.5, 33.9, 32.7, 30.8, 28.8, 27.4, 27.1, 23.7, 23.5, 21.3, 21.1, 20.5, 18.8, 18.2, 17.3, 14.1, 13.1; HRMS (ESI^+^): found [M+3H]^+^: 670.42317 (calculated for C_42_H_60_N_3_O_4,_ 670.45838).

#### 3.4.2. (4-(O-tolyl)-1*H*-1,2,3-triazol-1-yl) Methyl 3α-acetyloxy-11-oxo-urs-12-en-24-oate (**6b**)

Gummy white solid; yield = 87%; ^1^H NMR (600 MHz, chloroform-d) δ 7.78 (s, 1H), 7.39 (t, *J* = 7.5 Hz, 1H), 7.32 (dd, *J* = 20.4, 7.6 Hz, 2H), 7.26 (s, 1H), 5.50 (s, 1H), 5.34 (d, *J* = 12.6 Hz, 1H), 5.32–5.28 (m, 2H), 2.47 (dd, *J* = 13.7, 3.6 Hz, 1H), 2.36 (s, 1H), 2.16 (s, 3H), 2.05 (s, 3H), 2.02 (s, 1H), 1.83 (ddd, *J* = 23.3, 14.1, 5.9 Hz, 2H), 1.73 (s, 1H), 1.68 (s, 3H), 1.66–1.54 (m, 3H), 1.50 (d, *J* = 11.0 Hz, 1H), 1.47–1.40 (m, 3H), 1.36 (d, *J* = 12.3 Hz, 2H), 1.31 (s, 3H), 1.23 (t, *J* = 5.4 Hz, 2H), 1.16 (s, 3H), 1.10 (s, 3H), 1.01–0.97 (m, 1H), 0.91 (s, 3H), 0.87 (s, 3H), 0.78 (s, 3H), 0.77 (d, *J* = 6.3 Hz, 3H); ^13^C NMR (150 MHz, CDCl_3_) δ 199.2, 175.6, 170.1, 165.0, 142.1, 136.2, 133.6, 131.4, 130.4, 129.9, 126.9, 125.9, 125.6, 73.1, 60.2, 59.0, 57.4, 50.4, 46.7, 45.0, 43.7, 40.8, 39.2, 37.2, 34.5, 33.9, 32.7, 30.8, 28.8, 27.4, 27.2, 23.7, 23.5, 21.3, 21.1, 20.5, 18.8, 18.2, 17.8, 17.4, 14.1, 13.2; HRMS (ESI^+^): found [M+H]^+^: 684.45097 (calculated for C_42_H_58_N_3_O_5,_ 684.43765).

#### 3.4.3. (4-(Ortho-methoxyphenyl)-1*H*-1,2,3-triazol-1-yl) Methyl 3α-acetyloxy-11-oxo-urs-12-en-24-oate (**6c**)

Gummy white solid; yield = 89%; ^1^H NMR (600 MHz, chloroform-d) δ 8.12 (s, 1H), 7.71 (d, *J* = 7.8 Hz, 1H), 7.39 (t, *J* = 7.9 Hz, 1H), 7.11–7.01 (m, 2H), 5.48 (s, 1H), 5.30 (d, *J* = 7.2 Hz, 3H), 3.85 (s, 3H), 2.45 (dt, *J* = 13.5, 3.5 Hz, 1H), 2.35 (s, 1H), 2.15 (t, *J* = 15.2 Hz, 1H), 2.04 (s, 3H), 2.01 (s, 1H), 1.89–1.75 (m, 3H), 1.73–1.69 (m, 1H), 1.69–1.51 (m, 3H), 1.49 (d, *J* = 10.9 Hz, 1H), 1.45–1.39 (m, 3H), 1.34 (d, *J* = 12.2 Hz, 2H), 1.29 (s, 3H), 1.22 (t, *J* = 7.1 Hz, 2H), 1.18 (s, 1H), 1.15 (s, 3H), 1.09 (s, 3H), 1.00–0.96 (m, 1H), 0.90 (s, 3H), 0.85 (s, 3H), 0.77 (s, 3H), 0.75 (d, *J* = 6.4 Hz, 3H); ^13^C NMR (150 MHz, CDCl_3_) δ 199.2, 175.5, 170.1, 164.9, 151.1, 141.6, 130.4, 130.2, 126.3, 126.0, 125.4, 121.1, 112.2, 73.2, 60.2, 59.0, 57.5, 55.9, 50.4, 46.7, 45.0, 43.7, 40.8, 39.2, 37.2, 34.5, 33.9, 32.7, 30.8, 28.8, 27.5, 27.1, 23.7, 23.5, 21.3, 21.1, 20.5, 18.8, 18.2, 17.3, 14.1, 13.1; HRMS (ESI^+^): found [M+H]^+^: 700.43333 (calculated for C_42_H_58_N_3_O_6,_ 700.43256).

#### 3.4.4. (4-(Ortho-trifluoromethyl) phenyl)-1*H*-1,2,3-triazol-1-yl) Methyl 3α-acetyloxy-11-oxo-urs-12-en-24-oate (**6d**)

Gummy pale yellow-colour solid; yield = 96%; ^1^H NMR (600 MHz, Chloroform-d) δ 7.87 (s, 1H), 7.83 (d, *J* = 7.8 Hz, 1H), 7.73 (t, *J* = 7.8 Hz, 1H), 7.66 (t, *J* = 7.7 Hz, 1H), 7.49 (d, *J* = 7.8 Hz, 1H), 5.51 (d, *J* = 8.4 Hz, 1H), 5.30 (d, *J* = 11.6 Hz, 3H), 2.47 (dt, *J* = 13.6, 3.6 Hz, 1H), 2.36 (s, 1H), 2.15 (ddd, *J* = 18.2, 9.5, 3.6 Hz, 1H), 2.05 (d, *J* = 5.0 Hz, 3H), 2.01 (s, 2H), 1.85 (ddd, *J* = 20.2, 12.8, 5.6 Hz, 2H), 1.78 (dd, *J* = 12.7, 3.1 Hz, 1H), 1.74–1.68 (m, 2H), 1.64 (dd, *J* = 13.0, 4.1 Hz, 1H), 1.60–1.56 (m, 1H), 1.50 (d, *J* = 11.0 Hz, 1H), 1.46–1.40 (m, 3H), 1.38–1.34 (m, 2H), 1.30 (s, 3H), 1.22 (t, *J* = 7.1 Hz, 3H), 1.14 (s, 3H), 1.11 (s, 3H), 0.91 (s, 3H), 0.89 (s, 3H), 0.78 (s, 3H), 0.76 (d, *J* = 6.2 Hz, 3H); ^13^C NMR (150 MHz, CDCl_3_) δ 199.2, 175.5, 170.1, 165.0, 142.3, 134.5, 133.1, 130.5, 130.4, 128.9, 127.3, 126.8, 73.2, 60.2, 59.0, 57.2, 50.4, 46.7, 45.0, 43.7, 40.8, 39.3, 39.2, 37.2, 34.5, 33.9, 32.7, 30.8, 28.8, 27.4, 27.1, 23.6, 23.5, 21.3, 21.1, 21.0, 20.5, 18.8, 18.2, 17.4, 14.1, 13.1; ^19^F NMR (564 MHz, chloroform-*d*) δ −59.21; HRMS (ESI^+^): found [M+H]^+^: 738.40951 (calculated for C_42_H_55_F_3_N_3_O_5,_ 738.40938).

#### 3.4.5. (4-(Para-methoxyphenyl)-1*H*-1,2,3-triazol-1-yl) Methyl 3α-acetyloxy-11-oxo-urs-12-en-24-oate (**6e**)

Gummy pale yellow-colour solid; yield = 88%; ^1^H NMR (600 MHz, Chloroform-d) δ 7.92 (s, 1H), 7.56 (d, *J* = 8.5 Hz, 2H), 6.98 (d, *J* = 8.5 Hz, 2H), 5.48 (s, 1H), 5.28 (d, *J* = 5.7 Hz, 3H), 3.83 (s, 3H), 2.45 (dt, *J* = 13.5, 3.5 Hz, 1H), 2.35 (s, 1H), 2.19–2.11 (m, 1H), 2.04 (s, 3H), 2.01 (s, 2H), 1.86–1.75 (m, 3H), 1.70 (d, *J* = 13.7 Hz, 1H), 1.59 (ddd, *J* = 30.7, 14.3, 6.3 Hz, 2H), 1.49 (d, *J* = 11.1 Hz, 1H), 1.46–1.39 (m, 3H), 1.34 (d, *J* = 12.3 Hz, 2H), 1.29 (s, 3H), 1.22 (t, *J* = 7.1 Hz, 2H), 1.18 (d, *J* = 3.6 Hz, 1H), 1.14 (s, 3H), 1.09 (s, 3H), 1.00–0.96 (m, 1H), 0.90 (s, 3H), 0.85 (s, 3H), 0.77 (s, 3H), 0.75 (d, *J* = 6.3 Hz, 3H); ^13^C NMR (150 MHz, CDCl_3_) δ 199.2, 175.6, 170.1, 164.9, 159.9, 142.7, 130.4, 130.2, 122.5, 122.3, 114.8, 73.1, 60.3, 60.2, 59.0, 57.4, 55.6, 50.5, 46.7, 45.0, 43.7, 40.8, 39.3, 39.2, 37.2, 34.5, 33.9, 32.7, 30.8, 28.8, 27.4, 27.2, 23.7, 23.5, 21.2, 21.1, 20.5, 18.8, 18.2, 17.3, 14.1, 13.1; HRMS (ESI^+^): found [M+Na]^+^: 722.41206 (calculated for C_42_H_57_N_3_NaO_6_, 722.41451).

#### 3.4.6. (4-(Meta-trifluoromethyl) phenyl)-1*H*-1,2,3-triazol-1-yl) Methyl 3α-acetyloxy-11-oxo-urs-12-en-24-oate (**6f**)

Gummy red-colour solid; yield = 95%; ^1^H NMR (600 MHz, Chloroform-d) δ 8.13 (s, 1H), 8.04 (s, 1H), 7.95 (d, *J* = 7.8 Hz, 1H), 7.72 (dt, *J* = 15.7, 7.8 Hz, 2H), 5.53 (s, 1H), 5.34 (s, 3H), 2.51 (dt, *J* = 13.7, 3.4 Hz, 1H), 2.40 (s, 1H), 2.22–2.16 (m, 1H), 2.09 (s, 3H), 2.06 (s, 1H), 1.93–1.82 (m, 2H), 1.76 (d, *J* = 13.7 Hz, 3H), 1.70–1.64 (m, 1H), 1.61 (dt, *J* = 15.4, 3.5 Hz, 1H), 1.54 (d, *J* = 11.1 Hz, 1H), 1.51–1.44 (m, 3H), 1.40 (d, *J* = 11.6 Hz, 2H), 1.34 (s, 3H), 1.27 (dd, *J* = 8.9, 5.1 Hz, 2H), 1.23 (d, *J* = 4.2 Hz, 1H), 1.19 (s, 3H), 1.15 (s, 3H), 1.05–1.00 (m, 1H), 0.95 (s, 3H), 0.91 (s, 3H), 0.82 (s, 3H), 0.80 (d, *J* = 6.4 Hz, 3H); ^13^C NMR (150 MHz, CDCl_3_) δ 199.1, 175.6, 170.1, 165.0, 143.4, 137.1, 132.6, 130.6, 130.4, 125.5, 124.1, 123.5, 122.3, 117.5, 73.0, 60.1, 59.0, 57.2, 50.5, 46.7, 43.7, 40.8, 39.3, 39.2, 37.2, 34.5, 33.9, 32.7, 30.8, 28.8, 27.5, 27.2, 23.7, 23.5, 21.3, 21.1, 20.5, 18.8, 18.2, 17.3, 14.1, 13.1; ^19^F NMR (564 MHz, chloroform-*d*) δ −62.85; HRMS (ESI^+^): found [M+Na]^+^: 760.39146 (calculated for C_42_H_54_F_3_N_3_ NaO_5_, 760.39133).

#### 3.4.7. (4-(Meta-bromo) phenyl)-1*H*-1,2,3-triazol-1-yl) Methyl 3α-Acetyloxy-11-oxo-urs-12-en-24-oate (**6g**)

Gummy orange-colour solid; yield = 92%; ^1^H NMR (600 MHz, Chloroform-d) δ 8.02 (s, 1H), 7.90 (d, *J* = 1.9 Hz, 1H), 7.63 (dd, *J* = 8.1, 2.0 Hz, 1H), 7.55 (d, *J* = 8.0 Hz, 1H), 7.38 (t, *J* = 8.0 Hz, 1H), 5.49 (s, 1H), 5.28 (s, 3H), 2.46 (dt, *J* = 13.4, 3.4 Hz, 1H), 2.35 (s, 1H), 2.17–2.11 (m, 1H), 2.05 (s, 3H), 2.01 (d, *J* = 1.3 Hz, 2H), 1.85 (dt, *J* = 14.2, 7.1 Hz, 1H), 1.80–1.75 (m, 2H), 1.72–1.69 (m, 1H), 1.65–1.55 (m, 2H), 1.49 (d, *J* = 11.1 Hz, 1H), 1.45–1.41 (m, 2H), 1.36 (m, 1H), 1.35 (d, *J* = 11.9 Hz, 2H), 1.30 (s, 3H), 1.24–1.21 (m, 2H), 1.20–1.15 (m, 2H), 1.14 (s, 3H), 1.10 (s, 3H), 1.00–0.96 (m, 1H), 0.91 (s, 3H), 0.86 (s, 3H), 0.78 (s, 3H), 0.75 (d, *J* = 6.3 Hz, 3H); ^13^C NMR (150 MHz, CDCl_3_) δ 199.1, 175.6, 170.1, 164.9, 143.2, 137.7, 131.9, 131.1, 130.4, 123.7, 123.3, 122.2, 119.0, 73.1, 60.2, 59.0, 57.3, 50.4, 46.7, 45.0, 43.7, 40.8, 39.2, 37.2, 34.5, 33.9, 32.7, 30.8, 28.8, 27.5, 27.2, 23.7, 23.5, 21.3, 21.1, 20.5, 18.8, 18.2, 17.3, 14.1, 13.1; HRMS (ESI^+^): found [M+Na]^+^: 770.31261 (calculated for C_41_H_54_^79^BrN_3_O_5_Na, 770.31445). found [M+Na]^+^: 772.31313 (calculated for C_41_H_54_^81^BrN_3_O_5_Na, 772.31342).

#### 3.4.8. (4-(Para-bromo) phenyl)-1*H*-1,2,3-triazol-1-yl) Methyl 3α-acetyloxy-11-oxo-urs-12-en-24-oate (**6h**)

Gummy orange-colour solid; yield = 91%; ^1^H NMR (600 MHz, Chloroform-d) δ 8.00 (s, 1H), 7.63 (d, *J* = 8.3 Hz, 2H), 7.58 (d, *J* = 8.5 Hz, 2H), 5.49 (s, 1H), 5.29 (d, *J* = 4.5 Hz, 3H), 2.46 (dt, *J* = 13.5, 3.5 Hz, 1H), 2.35 (s, 1H), 2.14 (tt, *J* = 14.8, 3.3 Hz, 1H), 2.05 (s, 3H), 2.01 (s, 1H), 1.85 (dt, *J* = 14.0, 7.0 Hz, 1H), 1.74 (td, *J* = 18.1, 17.0, 7.6 Hz, 3H), 1.59 (ddd, *J* = 32.7, 14.2, 6.3 Hz, 2H), 1.49 (s, 1H), 1.47–1.38 (m, 3H), 1.38–1.32 (m, 2H), 1.30 (s, 3H), 1.23 (t, *J* = 6.9 Hz, 2H), 1.20–1.15 (m, 2H), 1.14 (s, 3H), 1.09 (s, 3H), 1.00–0.96 (m, 1H), 0.91 (s, 3H), 0.85 (s, 3H), 0.78 (s, 3H), 0.75 (d, *J* = 6.3 Hz, 3H); ^13^C NMR (150 MHz, CDCl_3_) δ 199.1, 175.6, 171.1, 170.1, 164.9, 143.2, 135.7, 132.9, 130.4, 122.6, 122.2, 122.0, 73.0, 60.2, 59.0, 57.3, 50.5, 46.7, 45.0, 43.7, 40.8, 39.3, 39.2, 37.2, 34.5, 33.9, 32.7, 30.8, 28.8, 27.5, 27.2, 23.7, 23.5, 21.2, 21.1, 20.5, 18.8, 18.2, 17.3, 14.1, 13.1; HRMS (ESI^+^): found [M+Na]^+^: 770.31480 (calculated for C_41_H_54_^79^BrN_3_O_5_Na, 770.31445). found [M+Na]^+^: 772.31427 (calculated for C_41_H_54_^81^BrN_3_O_5_Na, 772.31460).

#### 3.4.9. 4-(Para-chloro) phenyl)-1*H*-1,2,3-triazol-1-yl) Methyl 3α-acetyloxy-11-oxo-urs-12-en-24-oate (**6i**)

Gummy red-colour solid; yield = 93%; ^1^H NMR (600 MHz, Chloroform-d) δ 8.01 (s, 1H), 7.65 (dd, *J* = 8.8, 2.1 Hz, 2H), 7.53–7.44 (m, 2H), 5.50 (s, 1H), 5.30 (s, 3H), 2.47 (d, *J* = 13.3 Hz, 1H), 2.36 (s, 1H), 2.15 (t, *J* = 14.4 Hz, 1H), 2.06 (s, 3H), 2.02 (d, *J* = 2.0 Hz, 1H), 1.86 (dt, *J* = 14.0, 7.0 Hz, 1H), 1.81–1.70 (m, 3H), 1.66–1.56 (m, 2H), 1.50 (d, *J* = 11.1 Hz, 1H), 1.48–1.41 (m, 3H), 1.36 (d, *J* = 12.1 Hz, 2H), 1.31 (s, 3H), 1.26–1.22 (m, 2H), 1.18 (d, *J* = 15.6 Hz, 2H), 1.15 (s, 3H), 1.11 (s, 3H), 0.99 (dd, *J* = 14.8, 4.3 Hz, 1H), 0.92 (s, 3H), 0.86 (s, 3H), 0.79 (s, 3H), 0.76 (d, *J* = 6.3 Hz, 3H); ^13^C NMR (150 MHz, CDCl_3_) δ 199.2, 175.6, 170.1, 165.0, 143.2, 135.3, 134.8, 130.4, 130.0, 121.8, 73.0, 60.2, 59.0, 57.3, 50.5, 46.7, 45.0, 43.7, 40.8, 39.3, 39.2, 37.2, 34.5, 33.9, 32.7, 30.9, 28.8, 27.5, 27.2, 23.7, 23.5, 21.3, 21.1, 20.5, 18.8, 18.2, 17.4, 14.1, 13.1; HRMS (ESI^+^): found [M+H]^+^: 704.38440 (calculated for C_41_H_55_ClN_3_O_5_, 704.38302).

#### 3.4.10. 4-(Para-fluoro) phenyl)-1*H*-1,2,3-triazol-1-yl) Methyl 3α-acetyloxy-11-oxo-urs-12-en-24-oate (**6j**)

Gummy yellow-colour solid; yield = 94%; ^1^H NMR (600 MHz, Chloroform-d) δ 7.97 (s, 1H), 7.70–7.63 (m, 2H), 7.20 (t, *J* = 7.9 Hz, 2H), 5.50 (s, 1H), 5.30 (d, *J* = 3.6 Hz, 3H), 2.47 (d, *J* = 13.3 Hz, 1H), 2.36 (s, 1H), 2.18–2.12 (m, 1H), 2.05 (d, *J* = 1.8 Hz, 3H), 2.03 (s, 1H), 1.88–1.84 (m, 1H), 1.84–1.67 (m, 3H), 1.66–1.56 (m, 2H), 1.50 (d, *J* = 11.2 Hz, 1H), 1.47–1.40 (m, 3H), 1.35 (d, *J* = 12.2 Hz, 2H), 1.30 (s, 3H), 1.29–1.24 (m, 2H), 1.24–1.19 (m, 2H), 1.15 (s, 3H), 1.10 (s, 3H), 0.99 (dd, *J* = 14.3, 4.3 Hz, 1H), 0.91 (s, 3H), 0.86 (s, 3H), 0.78 (s, 3H), 0.76 (d, *J* = 6.3 Hz, 3H); ^13^C NMR (150 MHz, CDCl_3_) δ 199.2, 175.6, 170.1, 165.0, 163.3, 161.7, 143.0, 133.0, 130.4, 122.7, 122.6, 116.8, 116.7, 73.1, 60.2, 59.0, 57.3, 50.5, 46.7, 45.0, 43.7, 40.9, 39.2, 37.2, 34.5, 33.9, 32.7, 30.9, 28.8, 27.5, 27.2, 23.7, 23.5, 21.3, 21.1, 20.5, 18.8, 18.2, 17.4, 14.2, 13.1; ^19^F NMR (564 MHz, chloroform-*d*) δ −111.78; HRMS (ESI^+^): found [M+H]^+^: 688.41281 (calculated for C_41_H_55_FN_3_O_5_, 688.41258).

#### 3.4.11. 4-(Para-trifluoromethyl) phenyl)-1*H*-1,2,3-triazol-1-yl) Methyl 3α-acetyloxy-11-oxo-urs-12-en-24-oate (**6k**)

Gummy orange-colour solid; yield = 96%; ^1^H NMR (600 MHz, Chloroform-d) δ 8.09 (s, 1H), 7.86 (d, *J* = 8.2 Hz, 2H), 7.79 (d, *J* = 8.3 Hz, 2H), 5.50 (s, 1H), 5.31 (s, 3H), 2.46 (dd, *J* = 10.4, 7.0 Hz, 1H), 2.36 (s, 1H), 2.18–2.12 (m, 1H), 2.06 (s, 3H), 1.87 (dd, *J* = 13.7, 5.0 Hz, 1H), 1.80 (d, *J* = 13.1 Hz, 1H), 1.63 (td, *J* = 13.0, 3.9 Hz, 3H), 1.59–1.49 (m, 2H), 1.47–1.39 (m, 3H), 1.36 (d, *J* = 11.7 Hz, 2H), 1.31 (s, 3H), 1.27 (s, 1H), 1.23 (q, *J* = 5.1, 2.8 Hz, 2H), 1.23–1.17 (m, 2H), 1.16 (s, 3H), 1.11 (s, 3H), 1.02–0.97 (m, 1H), 0.91 (s, 3H), 0.85 (s, 3H), 0.78 (s, 3H), 0.76 (d, *J* = 6.3 Hz, 3H); ^13^C NMR (150 MHz, CDCl_3_) δ 199.1, 175.6, 170.1, 165.0, 143.4, 139.1, 130.4, 127.1, 122.2, 120.5, 73.0, 60.1, 59.0, 57.2, 50.5, 46.7, 45.0, 43.7, 40.8, 39.3, 39.2, 37.2, 34.5, 33.9, 32.7, 30.8, 28.8, 27.5, 27.2, 23.7, 23.5, 21.3, 21.1, 20.5, 18.8, 18.2, 17.4, 14.1, 13.1; ^19^F NMR (564 MHz, chloroform-*d*) δ −62.65; HRMS (ESI^+^): found [M+H]^+^: 738.41080 (calculated for C_42_H_55_F_3_N_3_O_5_, 738.40938).

#### 3.4.12. (4-Phenyl-1*H*-1,2,3-triazol-1-yl) Methyl 3α-hydroxy-11-oxo-urs-12-en-24-oate (**7a**)

Gummy pale yellow-colour solid; yield = 89%; ^1^H NMR (600 MHz, Chloroform-d) δ 8.01 (s, 1H), 7.70–7.63 (m, 2H), 7.53–7.47 (m, 2H), 7.45–7.40 (m, 1H), 5.48 (s, 1H), 5.34–5.24 (m, 2H), 2.43 (d, *J* = 13.6 Hz, 1H), 2.37 (s, 1H), 2.26–2.18 (m, 1H), 2.01 (d, *J* = 1.8 Hz, 2H), 1.85–1.76 (m, 3H), 1.69 (d, *J* = 14.1 Hz, 1H), 1.61 (dt, *J* = 13.2, 7.7 Hz, 1H), 1.53–1.47 (m, 2H), 1.46–1.34 (m, 5H), 1.34–1.28 (m, 2H), 1.28–1.26 (m, 6H), 1.24–1.21 (m, 2H), 1.18–1.13 (m, 1H), 1.09 (s, 3H), 1.00–0.94 (m, 1H), 0.90 (s, 3H), 0.86 (d, *J* = 1.9 Hz, 3H), 0.77 (d, *J* = 1.9 Hz, 3H), 0.75 (dd, *J* = 6.4, 1.8 Hz, 3H); ^13^C NMR (150 MHz, CDCl_3_) δ 199.4, 176.7, 165.0, 143.1, 136.8, 130.4, 129.8, 128.9, 122.2, 120.6, 70.5, 60.3, 59.0, 57.1, 48.8, 47.5, 45.0, 43.7, 40.9, 39.2, 37.3, 33.9, 33.8, 32.8, 30.9, 28.8, 27.4, 27.1, 26.2, 24.1, 21.1, 20.5, 18.9, 18.2, 17.4, 14.1, 13.1; HRMS (ESI^+^): found [M+H]^+^: 628.41011 (calculated for C_39_H_54_N_3_O_4_, 628.41143).

#### 3.4.13. (4-(Ortho-tolyl)-1*H*-1,2,3-triazol-1-yl) Methyl 3α-hydroxy-11-oxo-urs-12-en-24-oate (**7b**)

Gummy pale yellow-colour solid; yield = 86%; ^1^H NMR (600 MHz, Chloroform-d) δ 7.77 (s, 1H), 7.39 (t, *J* = 7.5 Hz, 1H), 7.37–7.28 (m, 2H), 7.26 (d, *J* = 7.8 Hz, 1H), 5.49 (d, *J* = 1.8 Hz, 1H), 5.35–5.25 (m, 2H), 2.43 (d, *J* = 13.5 Hz, 1H), 2.38 (s, 1H), 2.25–2.18 (m, 1H), 2.16 (d, *J* = 1.9 Hz, 3H), 2.09–2.03 (m, 1H), 2.01 (d, *J* = 1.7 Hz, 1H), 1.87–1.75 (m, 3H), 1.70 (d, *J* = 14.2 Hz, 1H), 1.62 (dt, *J* = 13.2 Hz, 1H), 1.50 (t, *J* = 13.1 Hz, 2H), 1.47–1.35 (m, 5H), 1.31 (d, *J* = 14.6 Hz, 2H), 1.28–1.26 (m, 6H), 1.24–1.22 (m, 2H), 1.19–1.15 (m, 1H), 1.09 (s, 3H), 1.00–0.95 (m, 1H), 0.91 (s, 3H), 0.86 (d, *J* = 1.7 Hz, 3H), 0.77 (d, *J* = 1.8 Hz, 3H), 0.75 (dd, *J* = 6.5, 1.7 Hz, 3H); ^13^C NMR (150 MHz, CDCl_3_) δ 199.4, 176.6, 136.2, 133.6, 131.4, 130.4, 129.9, 126.9, 125.9, 125.6, 70.5, 60.3, 59.0, 57.1, 48.8, 47.5, 45.0, 43.7, 40.9, 39.2, 37.4, 33.9, 33.8, 32.8, 30.9, 28.8, 27.4, 27.1, 26.2, 24.2, 21.1, 20.5, 18.9, 18.2, 17.7, 17.4, 14.1, 13.2; HRMS (ESI^+^): found [M+H]^+^: 642.42489 (calculated for C_40_H_56_N_3_O_4_, 642.42708).

#### 3.4.14. (4-(Ortho-methoxyphenyl)-1*H*-1,2,3-triazol-1-yl) Methyl 3α-hydroxy-11-oxo-urs-12-en-24-oate (**7c**)

Gummy pale yellow-colour solid; yield = 88%; ^1^H NMR (600 MHz, Chloroform-d) δ 8.11 (s, 1H), 7.71 (d, *J* = 7.9 Hz, 1H), 7.40 (t, *J* = 8.0 Hz, 1H), 7.07 (q, *J* = 8.0 Hz, 2H), 5.48 (d, *J* = 1.9 Hz, 1H), 5.34–5.27 (m, 2H), 3.86 (d, *J* = 1.8 Hz, 3H), 2.43 (d, *J* = 13.5 Hz, 1H), 2.37 (s, 1H), 2.23 (ddd, *J* = 16.5, 9.5, 3.2 Hz, 1H), 2.03 (dd, *J* = 18.1, 3.9 Hz, 3H), 1.82 (ddd, *J* = 17.5, 12.1, 4.2 Hz, 3H), 1.69 (d, *J* = 14.3 Hz, 1H), 1.65–1.59 (m, 1H), 1.50 (t, *J* = 14.4 Hz, 2H), 1.46–1.36 (m, 5H), 1.27 (t, *J* = 2.5 Hz, 6H), 1.23 (td, *J* = 7.2, 1.8 Hz, 3H), 1.18–1.15 (m, 1H), 1.09 (s, 3H), 0.99–0.95 (m, 1H), 0.90 (s, 3H), 0.86 (d, *J* = 1.9 Hz, 3H), 0.77 (d, *J* = 1.9 Hz, 3H), 0.76–0.74 (m, 3H); ^13^C NMR (150 MHz, CDCl_3_) δ 199.5, 176.6, 165.0, 151.1, 141.8, 130.4, 130.2, 126.2, 125.5, 121.2, 112.2, 70.5, 60.3, 59.0, 57.2, 55.9, 48.8, 47.5, 45.0, 43.7, 40.9, 39.2, 37.3, 33.9, 33.8, 32.8, 30.9, 28.8, 27.5, 27.1, 26.2, 24.2, 21.1, 20.5, 18.9, 18.2, 17.4, 14.1, 13.2; HRMS (ESI^+^): found [M+H]^+^: 658.42103 (calculated for C_40_H_56_N_3_O_5_, 658.42200).

#### 3.4.15. (4-(Ortho-trifluoromethyl) phenyl)-1*H*-1,2,3-triazol-1-yl) Methyl 3α-acetyloxy-11-oxo-urs-12-en-24-oate (**7d**)

Gummy white solid; yield = 96%; ^1^H NMR (600 MHz, Chloroform-d) δ 7.87 (s, 1H), 7.84 (d, *J* = 7.9 Hz, 1H), 7.73 (t, *J* = 7.8 Hz, 1H), 7.67 (t, *J* = 7.8 Hz, 1H), 7.50 (d, *J* = 7.8 Hz, 1H), 5.50 (d, *J* = 1.9 Hz, 1H), 5.30 (s, 2H), 2.44 (d, *J* = 13.6 Hz, 1H), 2.38 (s, 1H), 2.26–2.19 (m, 1H), 1.88–1.77 (m, 2H), 1.85–1.79 (m, 2H), 1.71–1.61 (m, 3H), 1.51 (t, *J* = 12.5 Hz, 2H), 1.47–1.36 (m, 5H), 1.31 (d, *J* = 14.9 Hz, 2H), 1.28–1.25 (m, 6H), 1.23 (td, *J* = 7.1, 1.9 Hz, 2H), 1.20–1.16 (m, 1H), 1.11 (s, 3H), 1.00–0.95 (m, 1H), 0.92–0.89 (m, 6H), 0.78 (d, *J* = 1.9 Hz, 3H), 0.76 (dd, *J* = 6.5, 1.8 Hz, 3H); ^13^C NMR (150 MHz, CDCl_3_) δ 199.4, 176.6, 165.0, 142.5, 134.6, 133.1, 130.5, 130.4, 128.9, 127.2, 126.7, 126.7, 70.5, 60.3, 59.0, 57.0, 48.8, 47.5, 45.0, 43.7, 40.9, 39.2, 37.4, 33.9, 33.8, 32.8, 30.9, 28.8, 27.5, 27.1, 26.2, 24.1, 21.1, 20.5, 18.9, 18.2, 17.4, 14.1, 13.1; ^19^F NMR (564 MHz, chloroform-*d*) δ −59.20; HRMS (ESI^+^): found [M+H]^+^: 696.39734 (calculated for C_40_H_53_F_3_N_3_O_4_, 696.39882).

#### 3.4.16. (4-(Para-methoxyphenyl)-1*H*-1,2,3-triazol-1-yl) Methyl 3α-hydroxy-11-oxo-urs-12-en-24-oate (**7e**)

Gummy white solid; yield = 87%; ^1^H NMR (600 MHz, Chloroform-*d*) δ 7.96 (s, 1H), 7.57 (d, J = 8.4 Hz, 2H), 7.00 (d, J = 8.2 Hz, 2H), 5.49 (s, 1H), 5.28 (s, 2H), 3.84 (d, J = 1.8 Hz, 3H), 2.44 (d, J = 13.7 Hz, 1H), 2.37 (s, 1H), 2.26–2.20 (m, 1H), 2.08–2.00 (m, 3H), 1.89–1.75 (m, 3H), 1.70 (s, 1H), 1.63 (d, J = 13.7 Hz, 1H), 1.49 (d, J = 11.8 Hz, 3H), 1.45–1.40 (m, 3H), 1.33 (d, J = 18.5 Hz, 2H), 1.27 (s, 6H), 1.24–1.22 (m, 2H), 1.18–1.15 (m, 1H), 1.10 (d, J = 6.4 Hz, 3H), 0.99–0.96 (m, 1H), 0.91 (s, 3H), 0.86 (s, 3H), 0.78 (s, 3H), 0.75 (d, J = 6.4 Hz, 3H); ^13^C NMR (150 MHz, CDCl_3_) δ 199.4, 176.6, 165.0, 159.9, 130.4, 122.3, 114.8, 70.5, 60.3, 59.0, 57.1, 55.6, 48.9, 47.5, 45.0, 43.7, 40.9, 39.2, 37.3, 33.9, 33.8, 32.8, 30.9, 28.8, 27.5, 27.1, 26.2, 24.1, 21.1, 20.5, 18.9, 18.2, 17.4, 14.1, 13.1; HRMS (ESI^+^): found [M+H]^+^: 658.42086 (calculated for C_40_H_56_N_3_O_5_, 658.42200)..4.17. (4-(meta-trifluoromethyl) phenyl)-1*H*-1,2,3-triazol-1-yl) Methyl 3α-hydroxy-11-oxo-urs-12-en-24-oate (**7f**)

Gummy pale yellow-colour solid; yield = 94%; ^1^H NMR (600 MHz, Chloroform-d) δ 8.09 (s, 1H), 7.99 (s, 1H), 7.91 (d, *J* = 8.0 Hz, 1H), 7.68 (dd, *J* = 17.2, 7.8 Hz, 2H), 5.49 (d, *J* = 1.9 Hz, 1H), 5.29 (s, 2H), 2.44 (d, *J* = 13.8 Hz, 1H), 2.38 (s, 1H), 2.26–2.19 (m, 1H), 2.03 (dd, *J* = 18.7, 3.5 Hz, 2H), 1.86–1.77 (m, 2H), 1.68 (dd, *J* = 24.7, 11.1 Hz, 3H), 1.52 (d, *J* = 18.9 Hz, 2H), 1.47–1.38 (m, 5H), 1.35–1.30 (m, 2H), 1.29–1.27 (m, 6H), 1.23 (dt, *J* = 7.1, 3.5 Hz, 2H), 1.17 (dd, *J* = 11.4, 3.0 Hz, 1H), 1.10 (s, 3H), 1.00–0.96 (m, 1H), 0.91 (s, 3H), 0.87 (d, *J* = 1.9 Hz, 3H), 0.78 (d, *J* = 1.9 Hz, 3H), 0.75 (dd, *J* = 6.5, 1.9 Hz, 3H); ^13^C NMR (150 MHz, CDCl_3_) δ 199.4, 176.7, 165.0, 137.1, 130.6, 130.4, 125.5, 123.5, 117.4, 70.5, 60.3, 60.3, 59.0, 56.9, 48.8, 47.5, 45.0, 43.7, 40.9, 39.2, 37.3, 33.9, 33.7, 32.8, 30.9, 28.8, 27.4, 27.1, 26.2, 24.1, 21.1, 20.5, 18.9, 18.2, 17.4, 14.1, 13.1; ^19^F NMR (564 MHz, chloroform-*d*) δ −62.85; HRMS (ESI^+^): found [M+H]^+^: 696.39788 (calculated for C_40_H_53_F_3_N_3_O_4_, 696.39882).

#### 3.4.17. (4-(Meta-bromo) phenyl)-1*H*-1,2,3-triazol-1-yl) Methyl 3α-hydroxy-11-oxo-urs-12-en-24-oate (**7g**)

Gummy white solid; yield = 91%; ^1^H NMR (600 MHz, Chloroform-d) δ 8.02 (s, 1H), 7.90 (d, *J* = 2.2 Hz, 1H), 7.64 (d, *J* = 8.1 Hz, 1H), 7.56 (d, *J* = 8.1 Hz, 1H), 7.38 (td, *J* = 8.1, 1.6 Hz, 1H), 5.49 (s, 1H), 5.28 (s, 2H), 2.44 (d, *J* = 13.7 Hz, 1H), 2.38 (s, 1H), 2.26–2.19 (m, 1H), 2.07–2.02 (m, 2H), 1.86–1.77 (m, 2H), 1.72–1.62 (m, 3H), 1.51 (dd, *J* = 17.6, 12.4 Hz, 2H), 1.47–1.37 (m, 5H), 1.31 (d, *J* = 14.6 Hz, 2H), 1.27 (d, *J* = 2.6 Hz, 6H), 1.24 (dd, *J* = 7.2, 1.6 Hz, 2H), 1.18–1.15 (m, 1H), 1.10 (s, 3H), 1.00–0.96 (m, 1H), 0.91 (s, 3H), 0.86 (s, 3H), 0.78 (s, 3H), 0.75 (dd, *J* = 6.5, 1.6 Hz, 3H); ^13^C NMR (150 MHz, CDCl_3_) δ 199.4, 176.7, 165.0, 131.9, 131.1, 130.4, 123.6, 123.3, 119.0, 70.5, 60.3, 60.3, 59.0, 57.0, 48.8, 47.5, 45.0, 43.7, 40.9, 39.2, 37.3, 33.9, 33.8, 32.8, 30.9, 28.8, 27.5, 27.1, 26.2, 24.1, 21.1, 20.5, 18.9, 18.3, 17.4, 14.1, 13.1; HRMS (ESI^+^): found [M+H]^+^: 706.32360 (calculated for C_39_H_52_^79^BrN_3_O_4_, 706.32194). found [M+H]^+^: 708.32173 (calculated for C_39_H_52_^81^BrN_3_O_4_ required 708.32190).

#### 3.4.18. (4-(Para-bromo) phenyl)-1*H*-1,2,3-triazol-1-yl) Methyl 3α-hydroxy-11-oxo-urs-12-en-24-oate (**7h**)

Gummy white solid; yield = 93%; ^1^H NMR (600 MHz, Chloroform-d) δ 8.00 (s, 1H), 7.64 (dd, *J* = 8.7, 1.9 Hz, 2H), 7.61–7.54 (m, 2H), 5.49 (s, 1H), 5.28 (s, 2H), 2.43 (d, *J* = 13.4 Hz, 1H), 2.37 (s, 1H), 2.22 (ddd, *J* = 15.8, 9.1, 3.1 Hz, 1H), 2.02 (d, *J* = 1.7 Hz, 2H), 1.86–1.75 (m, 2H), 1.72–1.63 (m, 3H), 1.50 (d, *J* = 14.0 Hz, 2H), 1.47–1.35 (m, 5H), 1.34–1.29 (m, 2H), 1.26 (s, 6H), 1.25–1.22 (m, 2H), 1.17 (dd, *J* = 11.9, 3.5 Hz, 1H), 1.09 (s, 3H), 0.97 (dd, *J* = 14.5, 4.0 Hz, 1H), 0.91 (s, 3H), 0.84 (s, 3H), 0.78 (d, *J* = 1.8 Hz, 3H), 0.76–0.74 (m, 3H); ^13^C NMR (150 MHz, CDCl_3_) δ 199.4, 176.7, 171.1, 165.0, 135.8, 132.9, 130.4, 122.6, 121.9, 70.5, 60.3, 59.0, 57.0, 48.8, 47.5, 45.0, 43.7, 40.9, 39.2, 37.3, 33.9, 33.7, 32.8, 30.9, 28.8, 27.5, 27.1, 26.2, 24.1, 21.1, 20.5, 18.9, 18.2, 17.4, 14.1, 13.1; HRMS (ESI^+^): found (M^+^): 706.32121 (clad. for C_39_H_52_^79^BrN_3_O_4_, 706.32194). found [M^+^]: 708.32030 (calculated for C_39_H_52_^81^BrN_3_O_4_, 708.32060).

#### 3.4.19. 4-(Para-chloro) phenyl)-1*H*-1,2,3-triazol-1-yl) Methyl 3α-acetyloxy-11-oxo-urs-12-en-24-oate (**7i**)

Gummy orange-colour solid; yield = 92%; ^1^H NMR (600 MHz, Chloroform-d) δ 7.99 (s, 1H), 7.68–7.59 (m, 2H), 7.55–7.44 (m, 2H), 5.49 (s, 1H), 5.27 (s, 2H), 2.46–2.40 (m, 1H), 2.37 (s, 1H), 2.21 (d, *J* = 15.4, 14.9, 3.9 Hz, 1H), 2.05–2.01 (m, 2H), 1.82 (ddd, *J* = 32.8, 16.2, 8.9 Hz, 2H), 1.72–1.59 (m, 3H), 1.51 (d, *J* = 15.7 Hz, 2H), 1.47–1.36 (m, 5H), 1.30 (d, *J* = 13.6 Hz, 2H), 1.26 (s, 6H), 1.24–1.22 (m, 2H), 1.18–1.15 (m, 1H), 1.09 (s, 3H), 1.00–0.95 (m, 1H), 0.91 (s, 3H), 0.84 (s, 3H), 0.77 (s, 3H), 0.75 (d, *J* = 6.3 Hz, 3H); ^13^C NMR (150 MHz, CDCl_3_) δ 199.4, 176.7, 165.1, 135.3, 134.7, 130.4, 130.0, 122.2, 121.7, 70.5, 60.3, 60.3, 59.0, 57.0, 48.8, 47.5, 45.0, 43.7, 40.9, 39.2, 37.3, 33.9, 33.7, 32.8, 30.8, 28.8, 27.4, 27.1, 26.2, 24.1, 21.1, 20.5, 18.9, 18.2, 17.4, 14.1, 13.1; HRMS (ESI^+^): found [M+H]^+^: 662.37150 (calculated for C_39_H_53_ClN_3_O_4_, 662.37246).

#### 3.4.20. 4-(Para-fluoro) phenyl)-1*H*-1,2,3-triazol-1-yl) Methyl 3α-acetyloxy-11-oxo-urs-12-en-24-oate (**7j**)

Gummy pale yellow-colour solid; yield = 94%; ^1^H NMR (600 MHz, Chloroform-d) δ 7.96 (d, *J* = 1.7 Hz, 1H), 7.65 (ddd, *J* = 8.9, 4.5, 1.7 Hz, 2H), 7.24–7.15 (m, 2H), 5.48 (s, 1H), 5.27 (dd, *J* = 4.8, 1.6 Hz, 2H), 2.45–2.39 (m, 1H), 2.37 (s, 1H), 2.26–2.17 (m, 1H), 2.01 (d, *J* = 1.6 Hz, 2H), 1.85–1.74 (m, 2H), 1.71–1.58 (m, 3H), 1.54–1.47 (m, 2H), 1.48–1.36 (m, 5H), 1.35–1.29 (m, 2H), 1.26 (s, 6H), 1.24–1.21 (m, 2H), 1.16 (dt, *J* = 13.9, 3.2 Hz, 1H), 1.09 (s, 3H), 0.99–0.95 (m, 1H), 0.90 (s, 3H), 0.84 (d, *J* = 1.7 Hz, 3H), 0.77 (d, *J* = 1.7 Hz, 3H), 0.75 (dd, *J* = 6.4, 1.6 Hz, 3H); ^13^C NMR (150 MHz, CDCl_3_) δ 199.5, 176.7, 165.1, 143.2, 133.0, 130.4, 122.6, 122.5, 122.4, 116.8, 116.7, 70.5, 60.3, 60.3, 59.0, 57.0, 48.8, 47.5, 45.0, 43.7, 40.9, 39.2, 37.3, 33.9, 33.7, 32.8, 30.8, 28.8, 27.4, 27.1, 26.2, 24.2, 21.1, 20.5, 18.9, 18.2, 17.4, 14.1, 13.1; ^19^F NMR (564 MHz, chloroform-*d*) δ −111.79; HRMS (ESI^+^): found [M+H]^+^: 646.40098 (calculated for C_39_H_53_FN_3_O_4_ required 646.40201).

#### 3.4.21. 4-(Para-trifluoromethyl) phenyl)-1*H*-1,2,3-triazol-1-yl) Methyl 3α-acetyloxy-11-oxo-urs-12-en-24-oate (**7k**)

Gummy white solid; yield = 96%; ^1^H NMR (600 MHz, Chloroform-d) δ 8.08 (s, 1H), 7.85 (d, *J* = 8.3 Hz, 2H), 7.78 (d, *J* = 8.3 Hz, 2H), 5.49 (s, 1H), 5.35–5.23 (m, 2H), 2.45–2.40 (m, 1H), 2.37 (s, 1H), 2.25–2.18 (m, 1H), 2.03 (dd, *J* = 19.6, 3.2 Hz, 2H), 1.86–1.76 (m, 2H), 1.72–1.59 (m, 3H), 1.54–1.48 (m, 2H), 1.47–1.35 (m, 5H), 1.35–1.29 (m, 2H), 1.27 (s, 6H), 1.23 (td, *J* = 7.1, 1.5 Hz, 2H), 1.19–1.15 (m, 1H), 1.09 (s, 3H), 0.99–0.95 (m, 1H), 0.91 (s, 3H), 0.84 (s, 3H), 0.77 (s, 3H), 0.75 (d, *J* = 6.3 Hz, 3H); ^13^C NMR (150 MHz, CDCl_3_) δ 199.4, 176.7, 171.1, 165.1, 143.6, 139.1, 130.4, 127.2, 127.1, 122.1, 120.5, 70.5, 60.2, 59.0, 56.9, 48.8, 47.5, 45.0, 43.7, 40.9, 39.2, 37.3, 33.9, 33.7, 32.8, 30.8, 28.8, 27.4, 27.1, 26.2, 24.1, 21.1, 20.5, 18.9, 18.2, 17.4, 14.1, 13.1; ^19^F NMR (564 MHz, chloroform-*d*) δ −62.66; HRMS (ESI^+^): found [M+H]^+^: 696.40558 (calculated for C_40_H_53_F_3_N_3_O_3_, 696.39882).

### 3.5. α-Glucosidase-Inhibitory Assay

The α-glucosidase (EC 3.2.1.20; *Saccharomyces cerevisiae*) assay was carried out using 50 mM of phosphate buffer (pH = 6.8). The test samples were dissolved in DMSO. The enzyme (1U/2mL) dissolved in phosphate buffer: 20 µL/well of enzyme, 20 µL/well of test samples, and 135 µL/well of phosphate buffer were employed in the 96-well plate. After adding all of these into the 96-well plate, followed by 15 min incubation at 37 °C, 25 µL/well of substrate *para*-nitro phenyl-α-D-glucopyranoside (0.7 mM) was added to the 96-well plate, and measurements were taken at 400 nm for 30 min using a spectrophotometer (xMarkTM Microplate, Bio-Rad, Hercules, CA, USA). As a control, 7% DMSO was employed. Acarbose was used as a standard inhibitor [35]. For mechanistic studies, a similar procedure was employed, with the addition of four different substrate concentrations: 0.1, 0.2, 0.4, and 0.8 µM. The percentage of inhibition was estimated using the following formula:

$$\%\;Inhibition=100\times \frac{O.D.\;of\;test\;compound}{O.D.\;of\;control}-100$$

### 3.6. In Silico Docking Studies

In this study, we conducted a docking experiment using Molecular Operating Environment (MOE version 2020.09) [46]. The X-ray crystal structure of α-glucosidase from *Saccharomyces cerevisiae* in a complex with ligand (α-D-glucopyranose) was taken from the Protein databank with PDB code 3A4A [47]. This structure was selected because of its high resolution, i.e., 1.6 Å. The protein file was prepared using the QuickPrep module of MOE, which adds missing hydrogen atoms to the protein structure, calculates the charges of each atom of protein (AMBER 10: EHT force field), and refines the structure after a short minimization, with a gradient of 0.1 kcal/mol/Å. The ligands files were prepared for docking by Chem Draw and converted into 3D-form by the MOE WASH module in the MOE database. WASH adds hydrogen atoms on the ligand and calculates the partial charges (AMBER 10: EHT force field) on each atom of the ligand. Moreover, in the database, WASG minimizes the structure of each ligand with the default parameter, i.e., gradient = 0.1 kcal/mol/Å. After preparing the protein file and ligand database, docking was performed using the Triangle Matcher placement method and London dG scoring function. Thirty docked conformations of each ligand were saved and, using the conformational sampling method, the best pose of each ligand was selected according to the docking score and ligand’s interactions with the enzyme.

## 4. Conclusions

A series of 24 novel 1*H*-1,2,3-triazole hybrids of β-AKBA (**6a–6k**) and β-KBA (**7a–7k**) was designed and synthesized by employing “click” chemistry in a highly efficient manner. The study of α-glucosidase inhibition determined that all the synthesized derivatives are highly potent inhibitors, with IC_50_ values ranging from 0.22 to 5.32 µM. Among all the synthesized compounds, nine compounds (**6f**, **7h**, **6j**, **6h**, **6g**, **6c**, **6k**, **7g**, and **7k**) exhibited the highest inhibition and were found to be several times more potent than the standard (acarbose). Furthermore, molecular docking was performed to discover the binding modes of these compounds with the α-glucosidase enzyme. The results showed that all compounds are accommodated well in the active site of α-glucosidase, where His280, Gln279, Asp215, His351, Arg442, and Arg315 are mainly stabilized the binding of these compounds. These encouraging results enable us to deeply explore the diabetic potential of boswellic acids hybrid triazoles in the field of medicinal chemistry. Furthermore, here, the α-glucosidase activity of all the active compounds is reported for the first time for these BA derivatives. The pharmacophoric features of substituted triazoles at the C-24 position of boswellic acids might be an interesting lead for the further development of new diabetic-like drug molecules.

## Figures and Tables

**Table 1:** Synthesis of 1*H*-1,2,3-triazole hybrids of β-AKBA a β-KBA.

Compounds	Azides (5a-k) R_1_-Group	1*H*-1,2,3-Triazole Products (6a–k and 7a–k)	Yields of Products % ^a^
**3**	Ph	**6a**	90
**3**	2-MeC_6_H_4_	**6b**	87
**3**	2-MeOC_6_H_4_	**6c**	89
**3**	2-F_3_CC_6_H_4_	**6d**	96
**3**	4-MeOC_6_H_4_	**6e**	88
**3**	3-F_3_CC_6_H_4_	**6f**	95
**3**	3-BrC_6_H_4_	**6g**	92
**3**	4-BrC_6_H_4_	**6h**	91
**3**	4-ClC_6_H_4_	**6i**	93
**3**	4-FC_6_H_4_	**6j**	94
**3**	4-F_3_CC_6_H_4_	**6k**	96
**4**	Ph	**7a**	89
**4**	2-MeC_6_H_4_	**7b**	86
**4**	2-MeOC_6_H_4_	**7c**	88
**4**	2-F_3_CC_6_H_4_	**7d**	96
**4**	4-MeOC_6_H_4_	**7e**	87
**4**	3-F_3_CC_6_H_4_	**7f**	94
**4**	3-BrC_6_H_4_	**7g**	91
**4**	4-BrC_6_H_4_	**7h**	93
**4**	4-ClC_6_H_4_	**7i**	92
**4**	4-FC_6_H_4_	**7j**	94
**4**	4-F_3_CC_6_H_4_	**7k**	96

^a^ Yields refer to pure isolated products.

**Table 2:** In vitro α-glucosidase-inhibitory activities of compounds **6a–6k** and **7a–7k**.

Compounds	IC_50_ ± µM	Compounds	IC_50_ ± µM	R_1_-Group
**6a**	2.02 ± 0.03	**7a**	2.31 ± 0.17	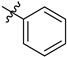
**6b**	2.25 ± 0.10	**7b**	2.11 ± 0.10	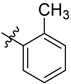
**6c**	1.23 ± 0.08	**7c**	2.18 ± 0.01	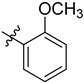
**6d**	2.37 ± 0.11	**7d**	2.62 ± 0.06	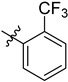
**6e**	2.01 ± 0.09	**7e**	2.37 ± 0.07	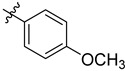
**6f**	0.22 ± 0.08	**7f**	2.52 ± 0.12	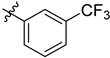
**6g**	0.78 ± 0.01	**7g**	1.34 ± 0.02	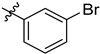
**6h**	0.69 ± 0.04	**7h**	0.43 ± 0.05	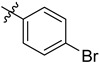
**6i**	3.06 ± 0.22	**7i**	3.18 ± 0.69	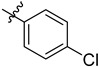
**6j**	0.58 ± 0.04	**7j**	2.64 ± 0.03	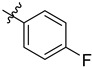
**6k**	1.26 ± 0.05	**7k**	1.39 ± 0.11	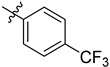
Acarbose (Standard)	942 ± 0.74	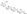		

## Data Availability

The spectroscopic data presented in this study are available in the supporting information.

## Supplementary Materials

The following supporting information can be downloaded at: https://www.mdpi.com/article/10.3390/ph16020229/s1. Figures S1–S80: ^1^H, ^13^C-NMR and HRMS spectra of the synthesized compounds (**3**,4 and **6a**–**6k**, and **7a**–**7k**); Figure S81: The re-docked conformation (green stick model) of maltose is superimposed on the X-ray conformation (grey stick model) present in the PDB code 3A4A; Table S1. The docking results of all the compounds; Table S2. Crystal data of the compound **4**.
