# Supplementary material for: Discovery of New Boswellic Acid Hybrid 1H-1,2,3-Triazoles for Diabetic Management: In Vitro and In Silico Studies
Source: Pharmaceuticals (Basel). 2023 Feb 2;16(2):229. doi: 10.3390/ph16020229 (PMC9960759; doi:10.3390/ph16020229)
Supplement: Supplementary file 1 [file pharmaceuticals-16-00229-s001.zip › pharmaceuticals-2159733-supplementary.pdf]

Discovery of New Boswellic Acid Hybrid 1*H*-1,2,3-Triazoles for Diabetic Management: In Vitro and In Silico Studies

Najeeb Ur Rehman [1], Saeed Ullah [1], Tanveer Alam [1], Sobia Ahsan Halim [1], Tapan Kumar Mohanta [1], Ajmal Khan [1], Muhammad U. Anwar [1], René Csuk [2], Satya Kumar Avula [1,*] and Ahmed Al-Harrasi [1,*]

[1] Natural & Medical Sciences Research Center, University of Nizwa, Nizwa 616, Oman
[2] Organic Chemistry, Martin-Luther-University Halle-Wittenberg, Kurt-Mothes-Str. 2, D-06120 Halle (Saale), Germany
* Correspondence: chemisatya@unizwa.edu.om (S.K.A.); aharrasi@unizwa.edu.om (A.A.-H.)

Contents

Table of Contents Page **Page**

**Figure S1**: ^{1}H-NMR spectrum (600 MHz, $CDCl_3$) of compound **3**	6
**Figure S2**: ^{13}C-NMR spectrum (150 MHz, $CDCl_3$) of compound **3**	7
**Figure S3**: HRMS spectrum of compound **3**	8
**Figure S4**: ^{1}H-NMR spectrum (600 MHz, $CDCl_3$) of compound **4**	9
**Figure S5**: ^{13}C-NMR spectrum (150 MHz, $CDCl_3$) of compound **4**	10
**Figure S6**: HRMS spectrum of compound **4**	11
**Figure S7**: ^{1}H-NMR spectrum (600 MHz, $CDCl_3$) of compound **6a**	12
**Figure S8**: ^{13}C-NMR spectrum (150 MHz, $CDCl_3$) of compound **6a**	13
**Figure S9**: HRMS spectrum of compound **6a**	14
**Figure S10**: ^{1}H-NMR spectrum (600 MHz, $CDCl_3$) of compound **6b**	15
**Figure S11**: ^{13}C-NMR spectrum (150 MHz, $CDCl_3$) of compound **6b**	16
**Figure S12**: HRMS spectrum of compound **6b**	17
**Figure S13**: ^{1}H-NMR spectrum (600 MHz, $CDCl_3$) of compound **6c**	18
**Figure S14**: ^{13}C-NMR spectrum (150 MHz, $CDCl_3$) of compound **6c**	19
**Figure S15**: HRMS spectrum of compound **6c**	20
**Figure S16**: ^{1}H-NMR spectrum (600 MHz, $CDCl_3$) of compound **6d**	21
**Figure S17**: ^{13}C-NMR spectrum (150 MHz, $CDCl_3$) of compound **6d**	22
**Figure S18**: ^{19}F-NMR spectrum (564 MHz, $CDCl_3$) of compound **6d**	23
**Figure S19**: HRMS spectrum of compound **6d**	24

**Figure S20**: ^{1}H-NMR spectrum (600 MHz, $CDCl_3$) of compound **6e**	25
**Figure S21**: ^{13}C-NMR spectrum (150 MHz, $CDCl_3$) of compound **6e**	26
**Figure S22**: HRMS spectrum of compound **6e**	27
**Figure S23**: ^{1}H-NMR spectrum (600 MHz, $CDCl_3$) of compound **6f**	28
**Figure S24**: ^{13}C-NMR spectrum (150 MHz, $CDCl_3$) of compound **6f**	29
**Figure S25**: ^{19}F-NMR spectrum (564 MHz, $CDCl_3$) of compound **6f**	30
**Figure S26**: HRMS spectrum of compound **6f**	31
**Figure S27**: ^{1}H-NMR spectrum (600 MHz, $CDCl_3$) of compound **6g**	32
**Figure S28**: ^{13}C-NMR spectrum (150 MHz, $CDCl_3$) of compound **6g**	33
**Figure S29**: HRMS spectrum of compound **6g**	34
**Figure S30**: ^{1}H-NMR spectrum (600 MHz, $CDCl_3$) of compound **6h**	35
**Figure S31**: ^{13}C-NMR spectrum (150 MHz, $CDCl_3$) of compound **6h**	36
**Figure S32**: HRMS spectrum of compound **6h**	37
**Figure S33**: ^{1}H-NMR spectrum (600 MHz, $CDCl_3$) of compound **6i**	38
**Figure S34**: ^{13}C-NMR spectrum (150 MHz, $CDCl_3$) of compound **6i**	39
**Figure S35**: HRMS spectrum of compound **6i**	40
**Figure S36**: ^{1}H-NMR spectrum (600 MHz, $CDCl_3$) of compound **6j**	41
**Figure S37**: ^{13}C-NMR spectrum (150 MHz, $CDCl_3$) of compound **6j**	42
**Figure S38**: ^{19}F-NMR spectrum (564 MHz, $CDCl_3$) of compound **6j**	43
**Figure S39**: HRMS spectrum of compound **6j**	44
**Figure S40**: ^{1}H-NMR spectrum (600 MHz, $CDCl_3$) of compound **6k**	45

**Figure S41**: ^{13}C-NMR spectrum (150 MHz, $CDCl_3$) of compound **6k**	46
**Figure S42**: ^{19}F-NMR spectrum (564 MHz, $CDCl_3$) of compound **6k**	47
**Figure S43**: HRMS spectrum of compound **6k**	48
**Figure S44**: ^{1}H-NMR spectrum (600 MHz, $CDCl_3$) of compound **7a**	49
**Figure S45**: ^{13}C-NMR spectrum (150 MHz, $CDCl_3$) of compound **7a**	50
**Figure S46**: HRMS spectrum of compound **7a**	51
**Figure S47**: ^{1}H-NMR spectrum (600 MHz, $CDCl_3$) of compound **7b**	52
**Figure S48**: ^{13}C-NMR spectrum (150 MHz, $CDCl_3$) of compound **7b**	53
**Figure S49**: HRMS spectrum of compound **7b**	54
**Figure S50**: ^{1}H-NMR spectrum (600 MHz, $CDCl_3$) of compound **7c**	55
**Figure S51**: ^{13}C-NMR spectrum (150 MHz, $CDCl_3$) of compound **7c**	56
**Figure S52**: HRMS spectrum of compound **7c**	57
**Figure S53**: ^{1}H-NMR spectrum (600 MHz, $CDCl_3$) of compound **7d**	58
**Figure S54**: ^{13}C-NMR spectrum (150 MHz, $CDCl_3$) of compound **7d**	59
**Figure S55**: ^{19}F-NMR spectrum (564 MHz, $CDCl_3$) of compound **7d**	60
**Figure S56**: HRMS spectrum of compound **7d**	61
**Figure S57**: ^{1}H-NMR spectrum (600 MHz, $CDCl_3$) of compound **7e**	62
**Figure S58**: ^{13}C-NMR spectrum (150 MHz, $CDCl_3$) of compound **7e**	63
**Figure S59**: HRMS spectrum of compound **7e**	64
**Figure S60**: ^{1}H-NMR spectrum (600 MHz, $CDCl_3$) of compound **7f**	65
**Figure S61**: ^{13}C-NMR spectrum (150 MHz, $CDCl_3$) of compound **7f**	66

**Figure S62**: ^{19}F-NMR spectrum (564 MHz, $CDCl_3$) of compound **7f**	67
**Figure S63**: HRMS spectrum of compound **7f**	68
**Figure S64**: ^{1}H-NMR spectrum (600 MHz, $CDCl_3$) of compound **7g**	69
**Figure S65**: ^{13}C-NMR spectrum (150 MHz, $CDCl_3$) of compound **7g**	70
**Figure S66**: HRMS spectrum of compound **7g**	71
**Figure S67**: ^{1}H-NMR spectrum (600 MHz, $CDCl_3$) of compound **7h**	72
**Figure S68**: ^{13}C-NMR spectrum (150 MHz, $CDCl_3$) of compound **7h**	73
**Figure S69**: HRMS spectrum of compound **7h**	74
**Figure S70**: ^{1}H-NMR spectrum (600 MHz, $CDCl_3$) of compound **7i**	75
**Figure S71**: ^{13}C-NMR spectrum (150 MHz, $CDCl_3$) of compound **7i**	76
**Figure S72**: HRMS spectrum of compound **7i**	77
**Figure S73**: ^{1}H-NMR spectrum (600 MHz, $CDCl_3$) of compound **7j**	78
**Figure S74**: ^{13}C-NMR spectrum (150 MHz, $CDCl_3$) of compound **7j**	79
**Figure S75**: ^{19}F-NMR spectrum (564 MHz, $CDCl_3$) of compound **7j**	80
**Figure S76** HRMS spectrum of compound **7j**	81
**Figure S77**: ^{1}H-NMR spectrum (600 MHz, $CDCl_3$) of compound **7k**	82
**Figure S78**: ^{13}C-NMR spectrum (150 MHz, $CDCl_3$) of compound **7k**	83
**Figure S79**: ^{19}F-NMR spectrum (564 MHz, $CDCl_3$) of compound **7k**	84
**Figure S80**: HRMS spectrum of compound **7k**	85

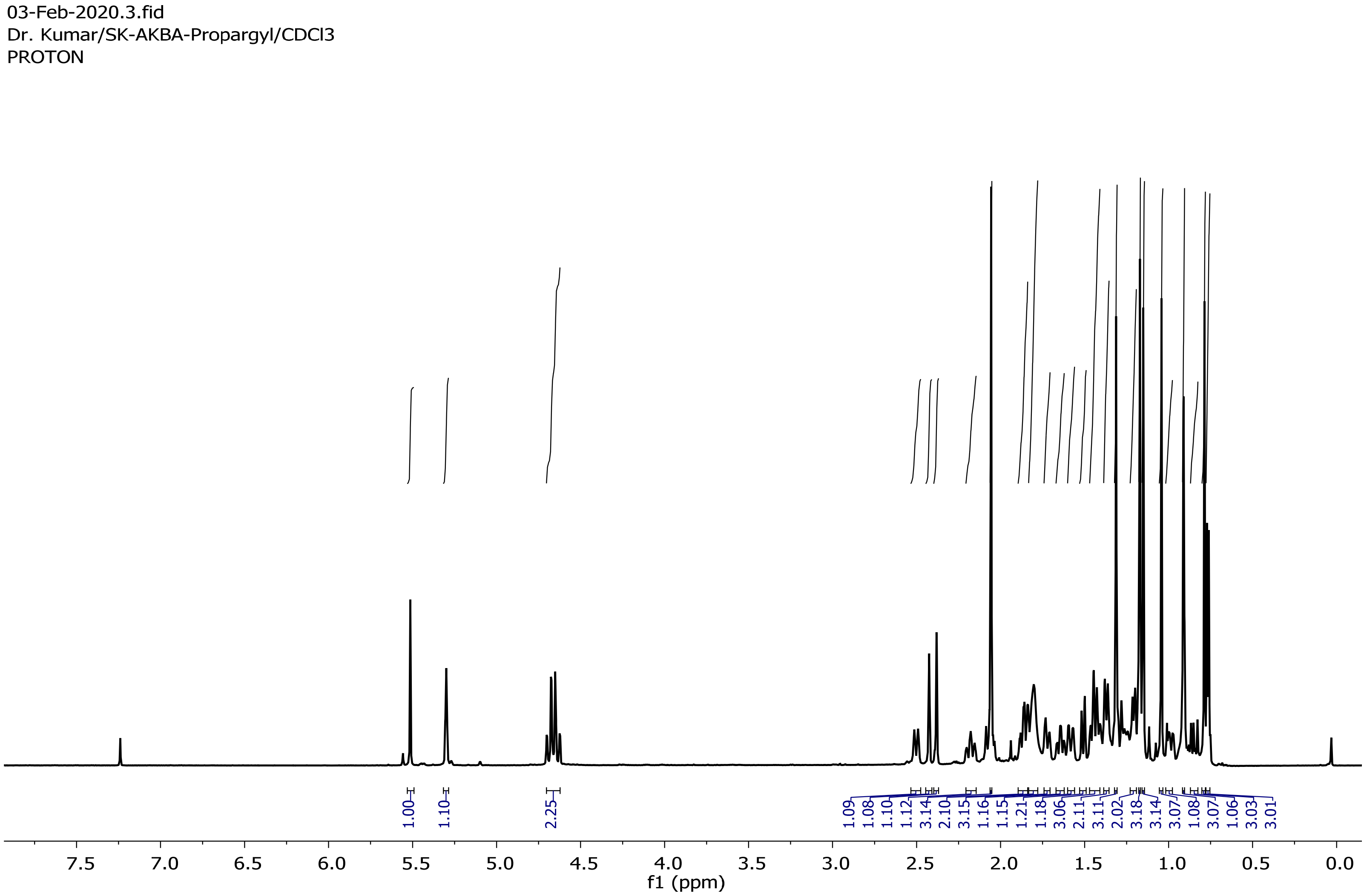

Figure S1: ^{1}H-NMR spectrum (600 MHz, $CDCl_3$) of compound **3**

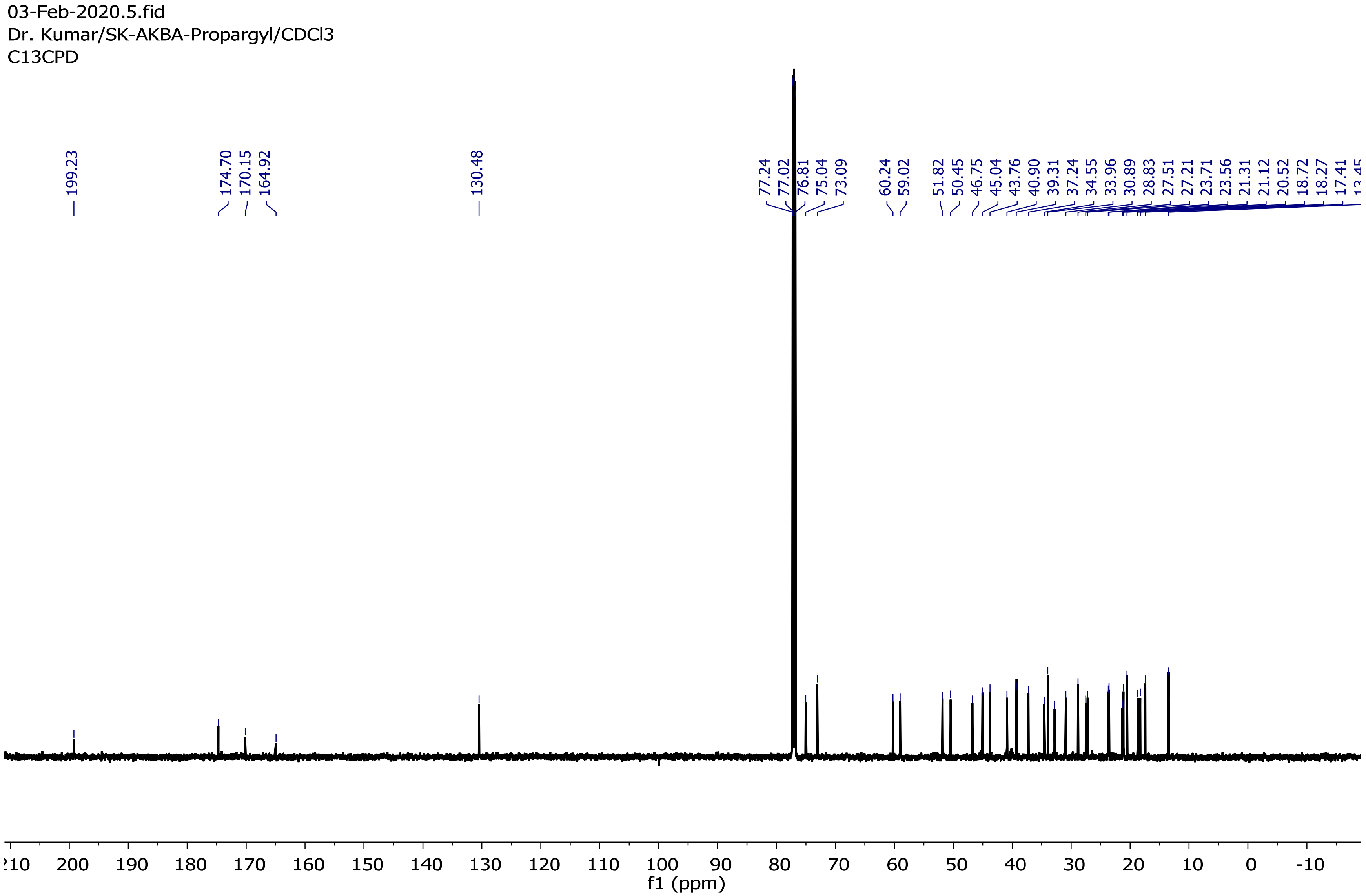

Figure S2: ^{13}C-NMR spectrum (150 MHz, $CDCl_3$) of compound **3**

**Sample Name**	SK-AKBA-PROP	**Position**	Vial 21	**Instrument Name**	Instrument 1
**User Name**		**Inj Vol**	2	**InjPosition**	
**Sample Type**	Sample	**IRM Calibration Status**	Success	**Data Filename**	SK-AKBA-PROP_POS_01.d
**ACQ Method**	POSITIVE ION METHOD MS.m	**Comment**	SK	**Acquired Time**	18-Oct-20 12:57:03 PM

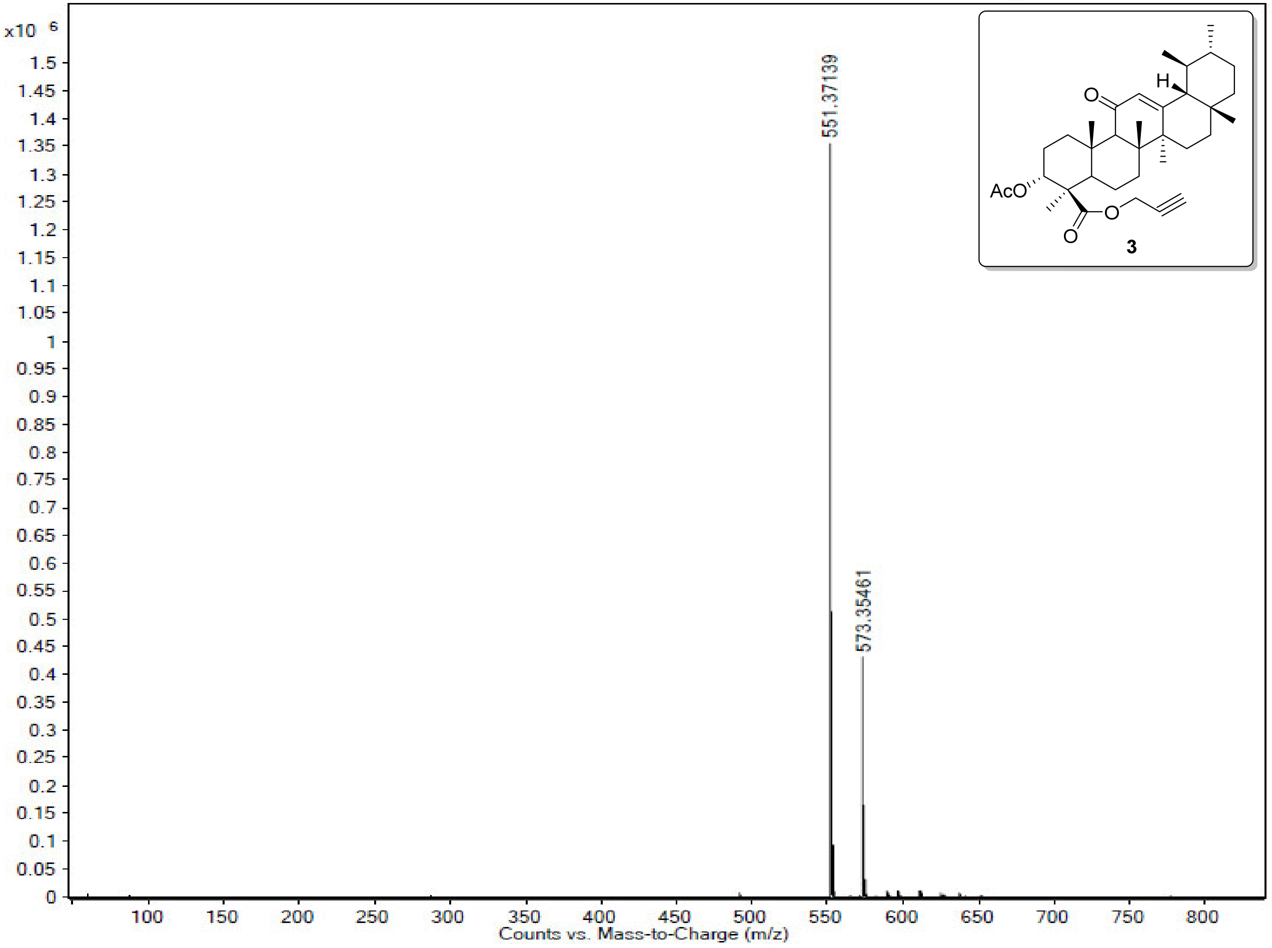

Figure S3: HRMS spectrum of compound **3**

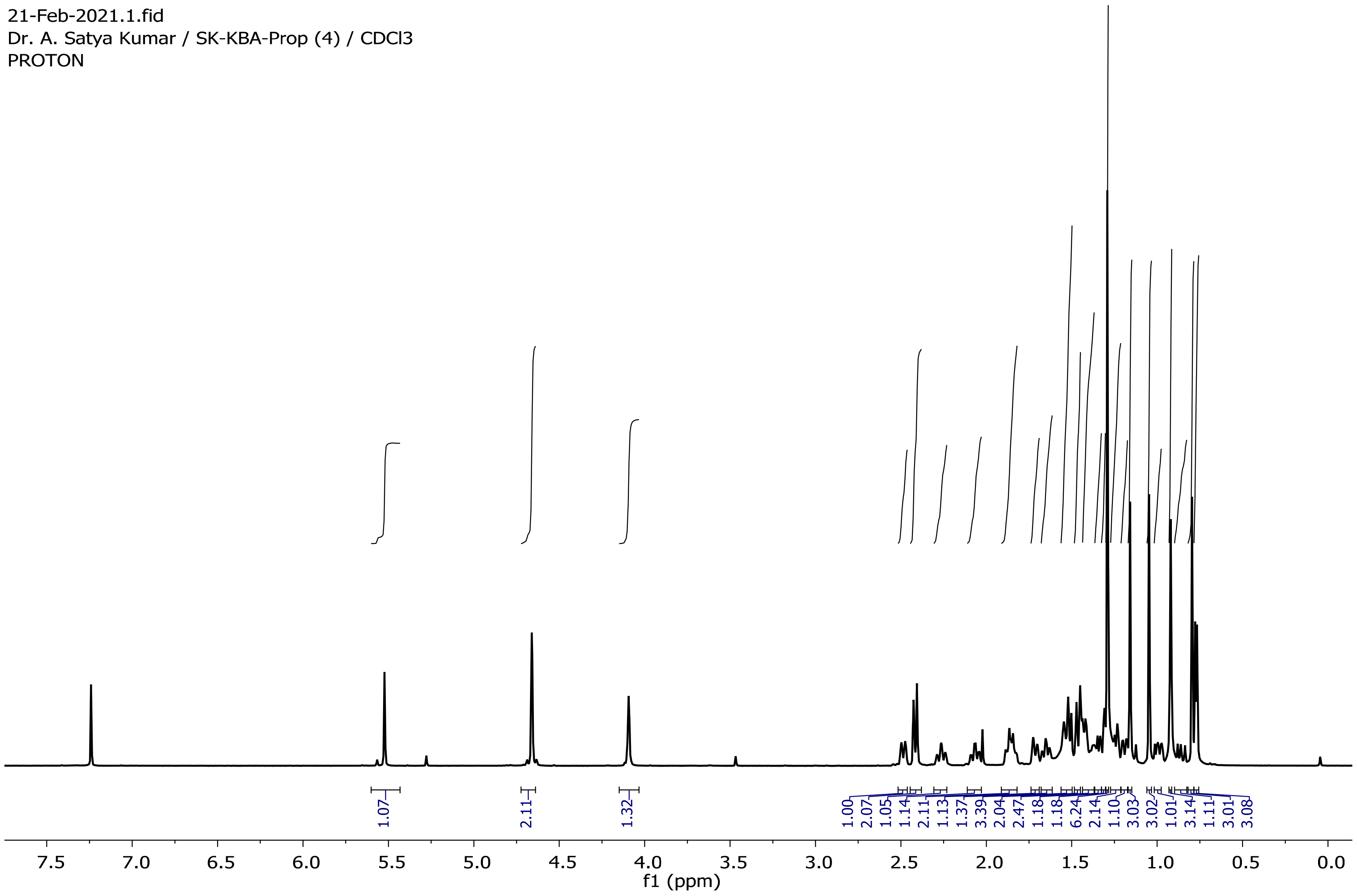

Figure S4: ^{1}H-NMR spectrum (600 MHz, $CDCl_3$) of compound **4**

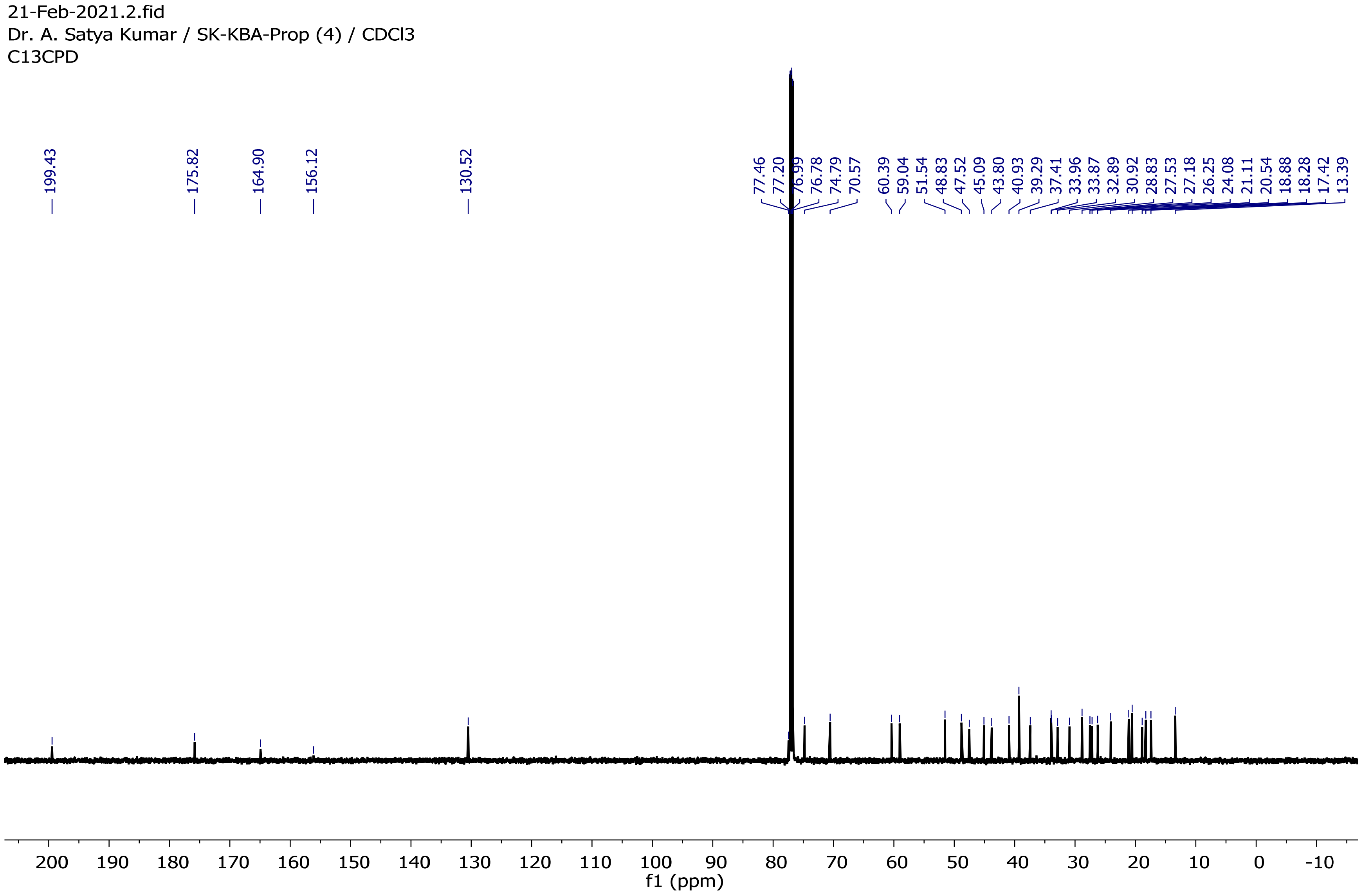

Figure S5: ^{13}C-NMR spectrum (150 MHz, $CDCl_3$) of compound **4**

**Sample Name**	KBA-PRO-A	**Position**	Vial 62	**Instrument Name**	Instrument 1
**User Name**		**Inj Vol**	2	**InjPosition**	
**Sample Type**	Sample	**IRM Calibration Status**	Success	**Data Filename**	KBA-PRO-A_POS.d
**ACQ Method**	POSITIVE ION METHOD MS.m	**Comment**		**Acquired Time**	24-Feb-21 12:20:31 PM

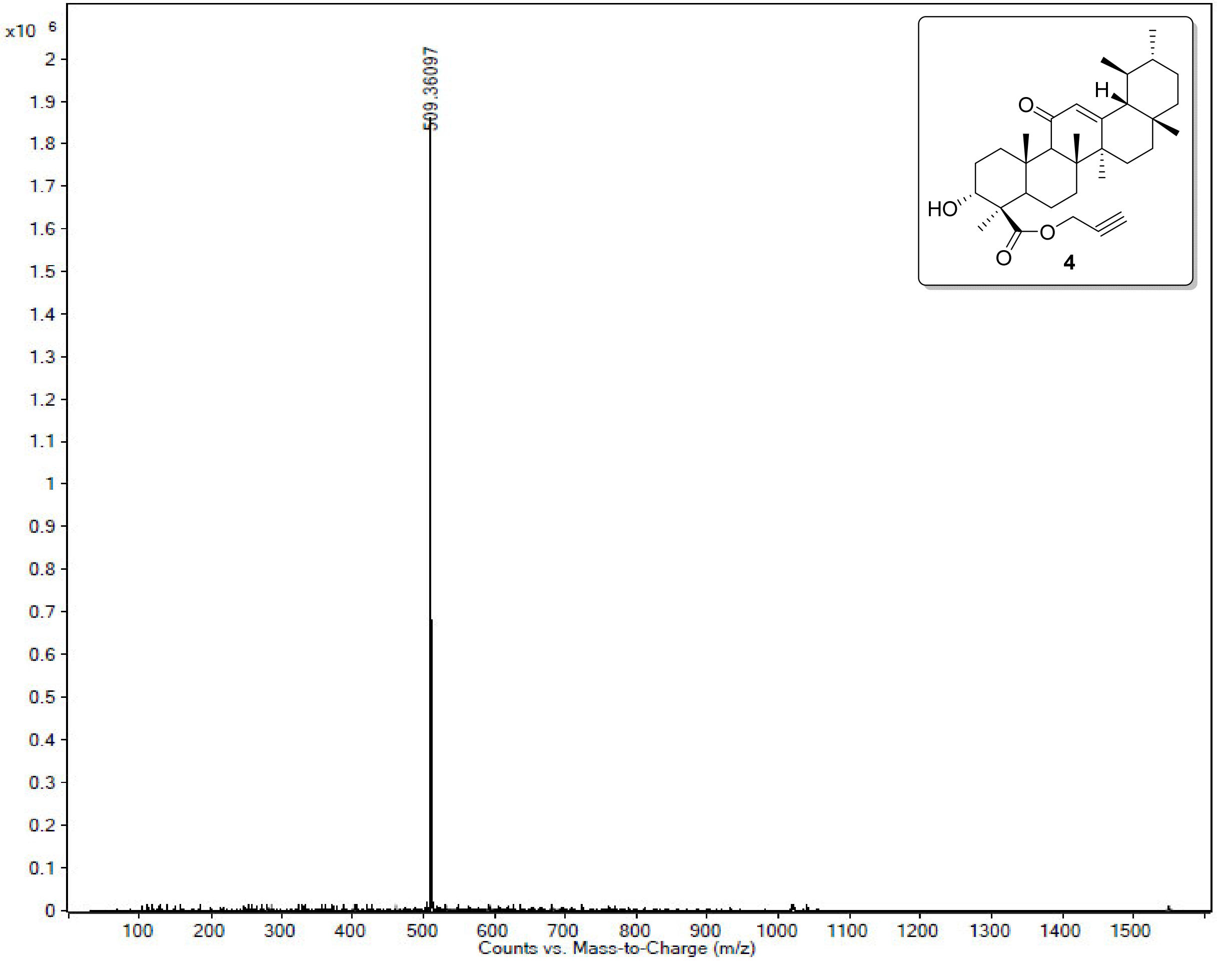

Figure S6: HRMS spectrum of compound **4**

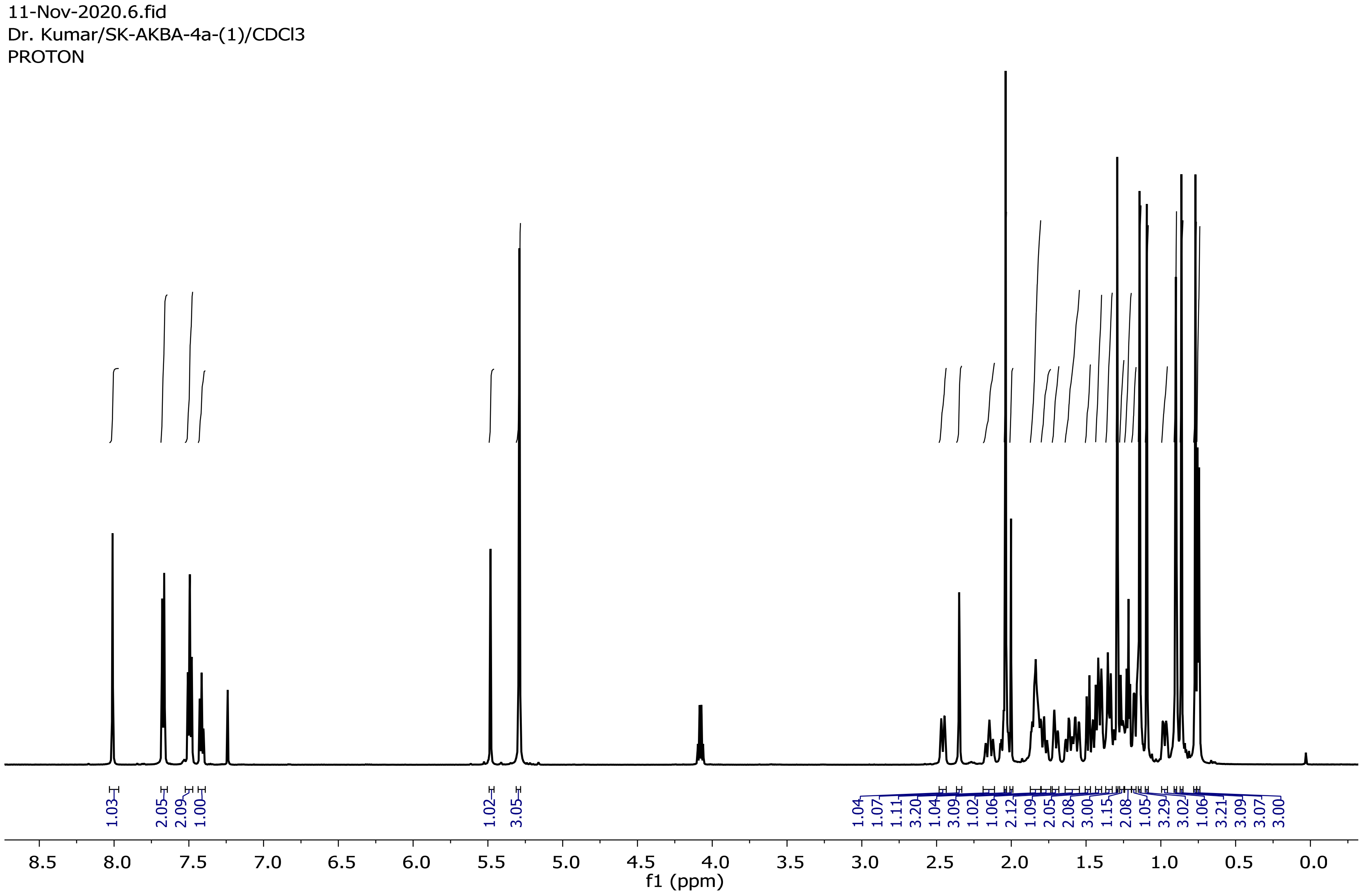

Figure S7: ^{1}H-NMR spectrum (600 MHz, $CDCl_3$) of compound **6a**

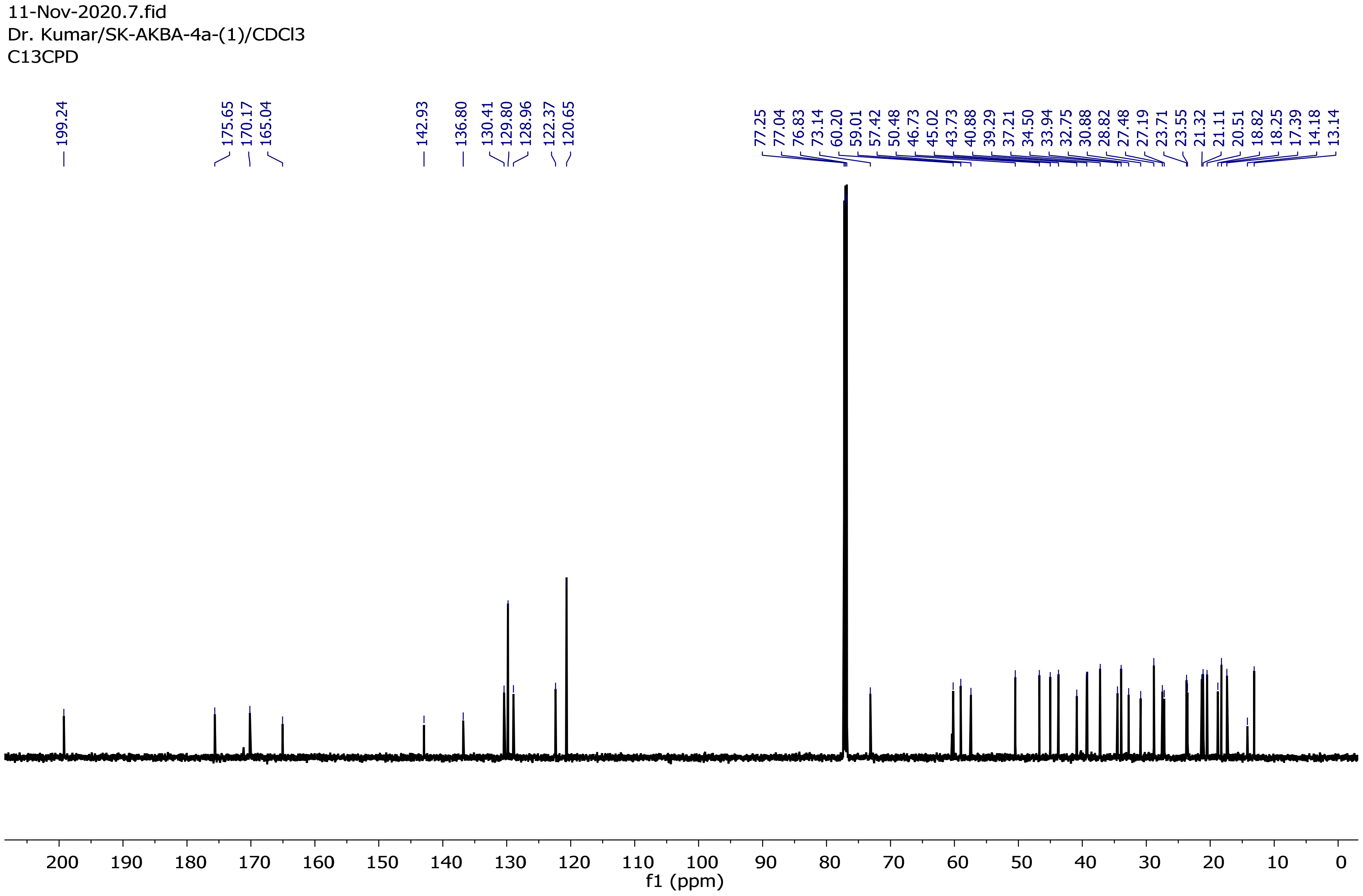

Figure S8: ^{13}C-NMR spectrum (150 MHz, $CDCl_3$) of compound **6a**

**Sample Name**	SK-AKBA-4A	**Position**	Vial 14	**Instrument Name**	Instrument 1
**User Name**		**Inj Vol**	2	**InjPosition**	
**Sample Type**	Sample	**IRM Calibration Status**	Success	**Data Filename**	SK-AKBA-4A_POS_01.d
**ACQ Method**	POSITIVE ION METHOD MS.m	**Comment**	SK	**Acquired Time**	11-Nov-20 3:19:42 PM

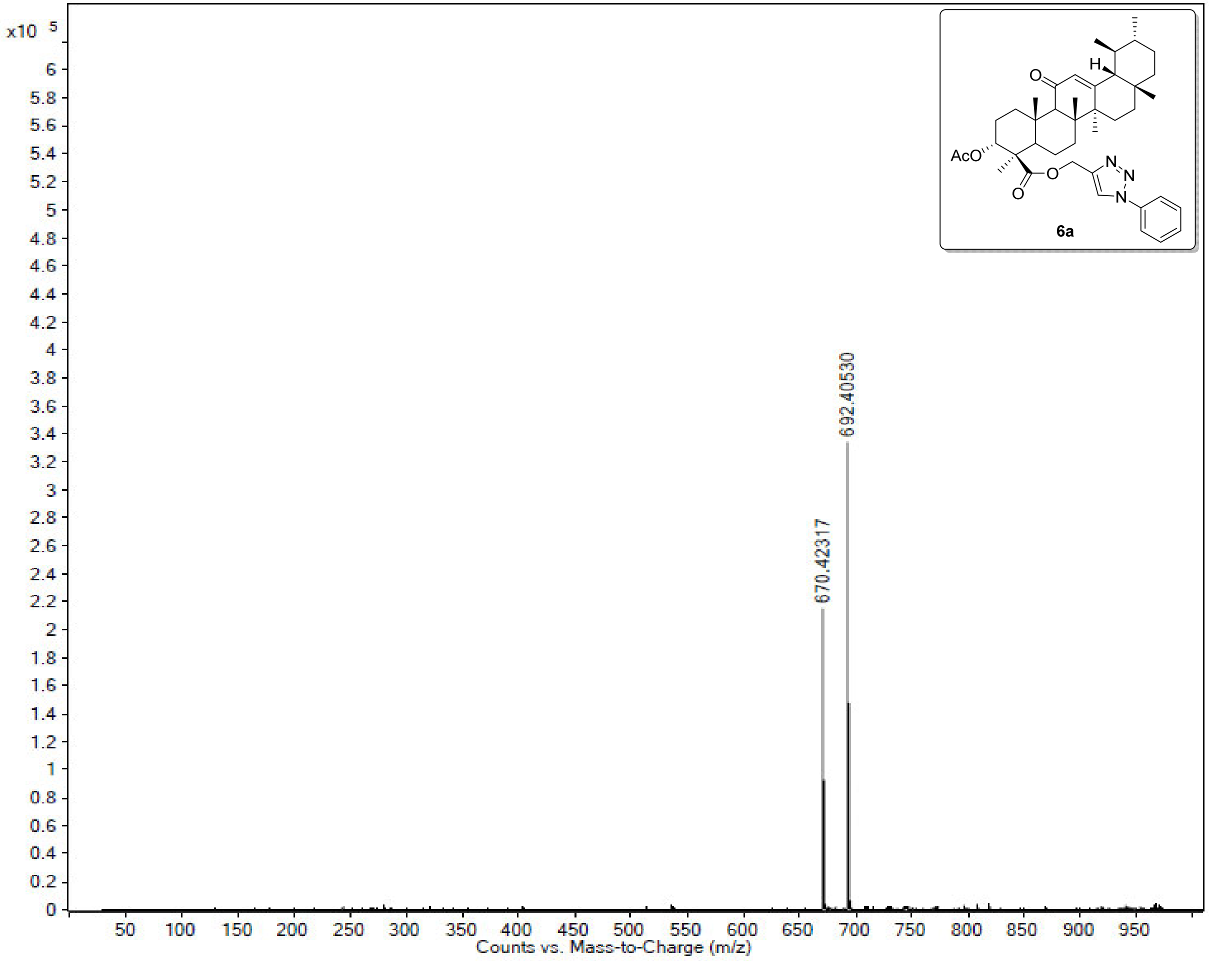

Figure S9: HRMS spectrum of compound **6a**

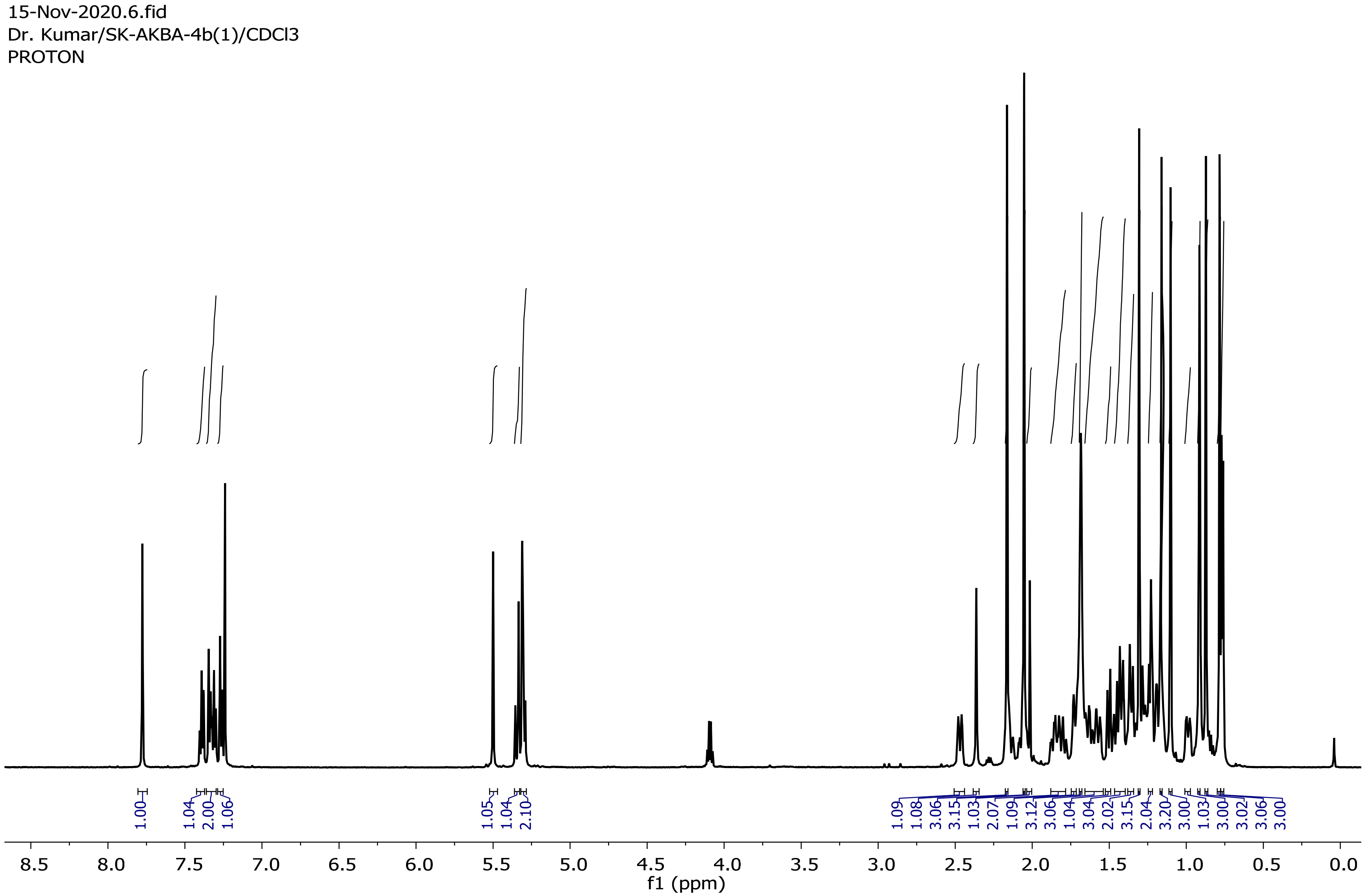

Figure S10: ^{1}H-NMR spectrum (600 MHz, $CDCl_3$) of compound **6b**

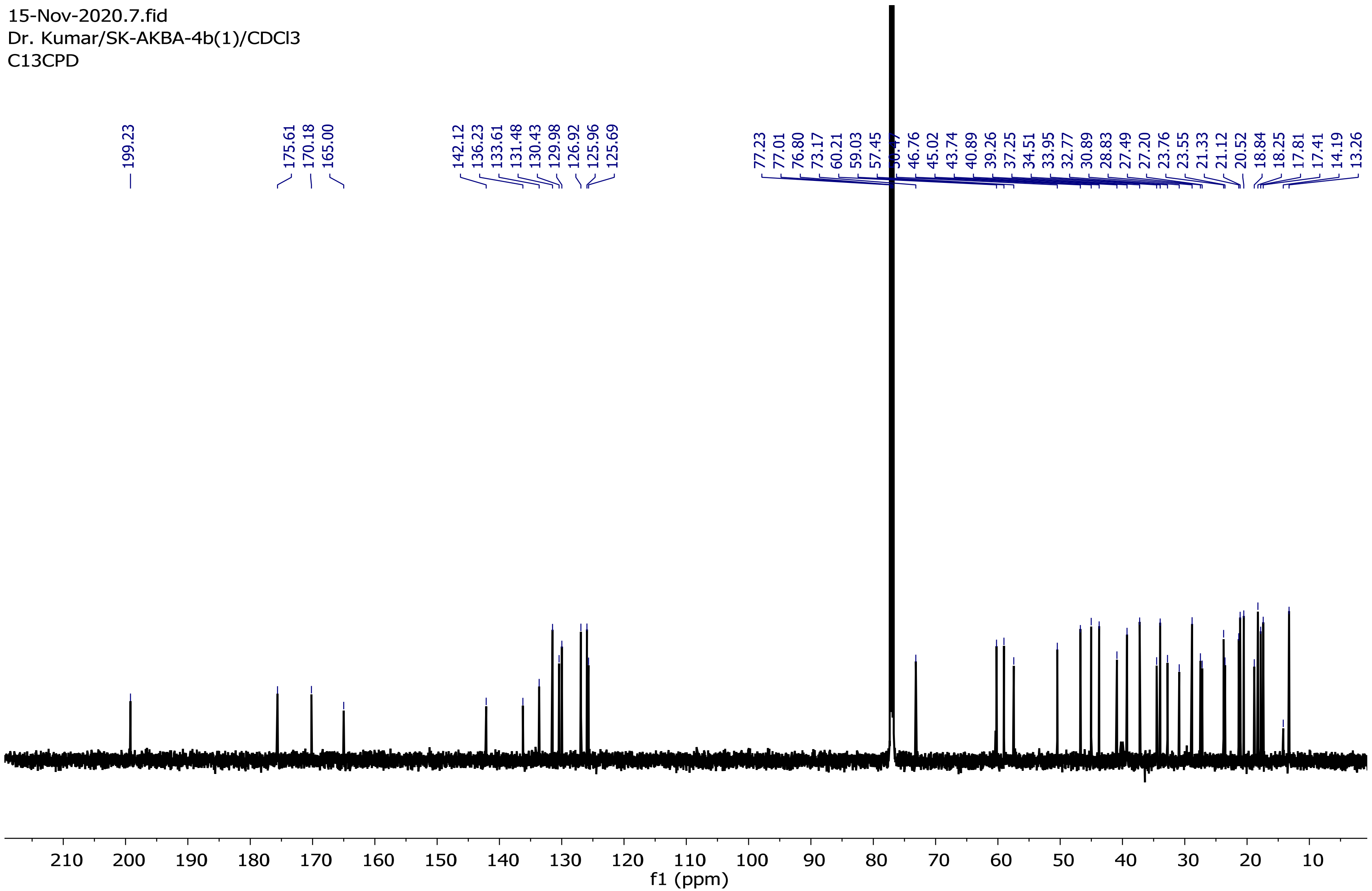

Figure S11: ^{13}C-NMR spectrum (150 MHz, $CDCl_3$) of compound **6b**

**Sample Name**	SK-AKBA-4B	**Position**	Vial 15	**Instrument Name**	Instrument 1
**User Name**		**Inj Vol**	2	**InjPosition**	
**Sample Type**	Sample	**IRM Calibration Status**	Success	**Data Filename**	SK-AKBA-4B_POS_01.d
**ACQ Method**	POSITIVE ION METHOD MS.m	**Comment**	SK	**Acquired Time**	11-Nov-20 3:25:23 PM

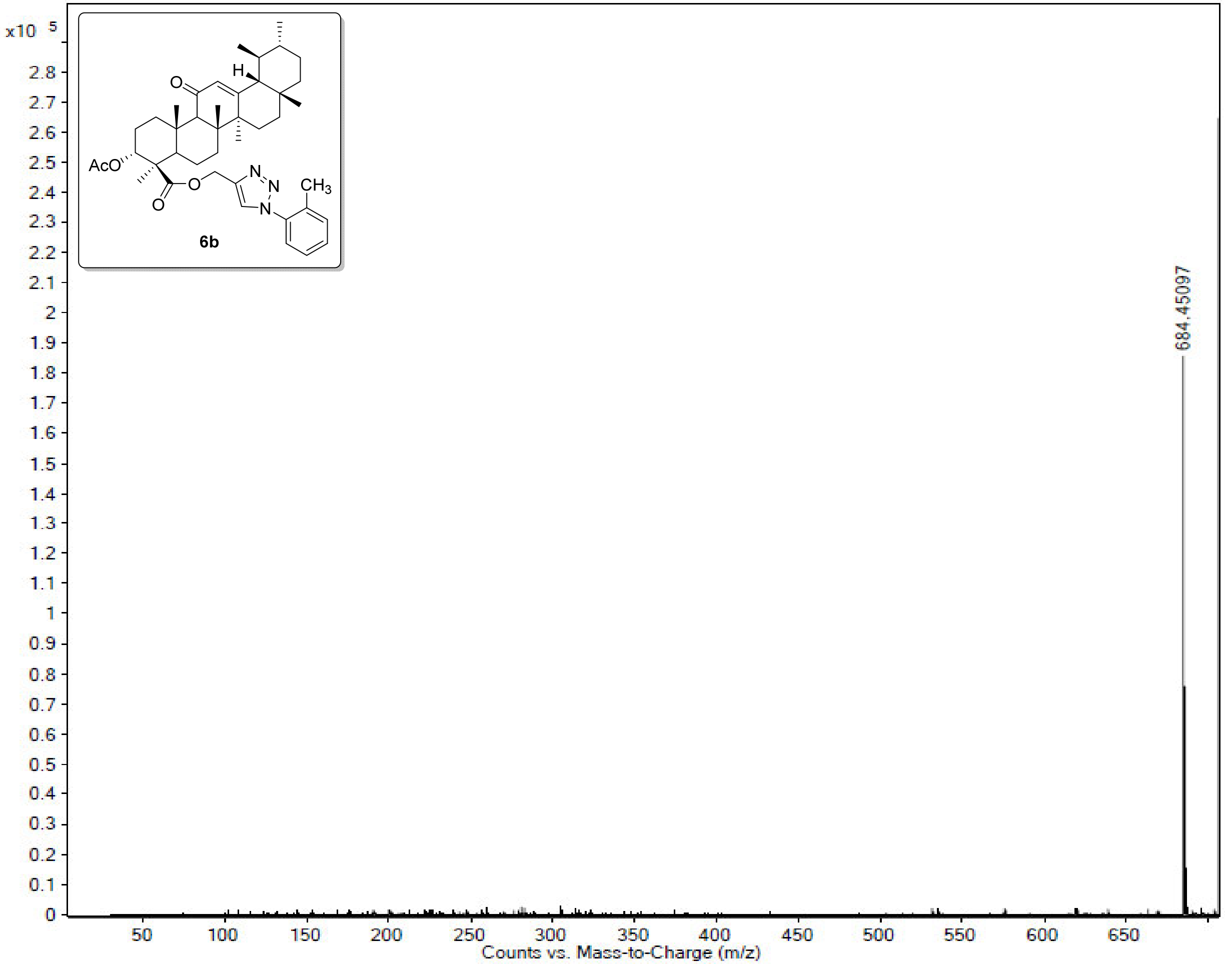

Figure S12: HRMS spectrum of compound **6b**

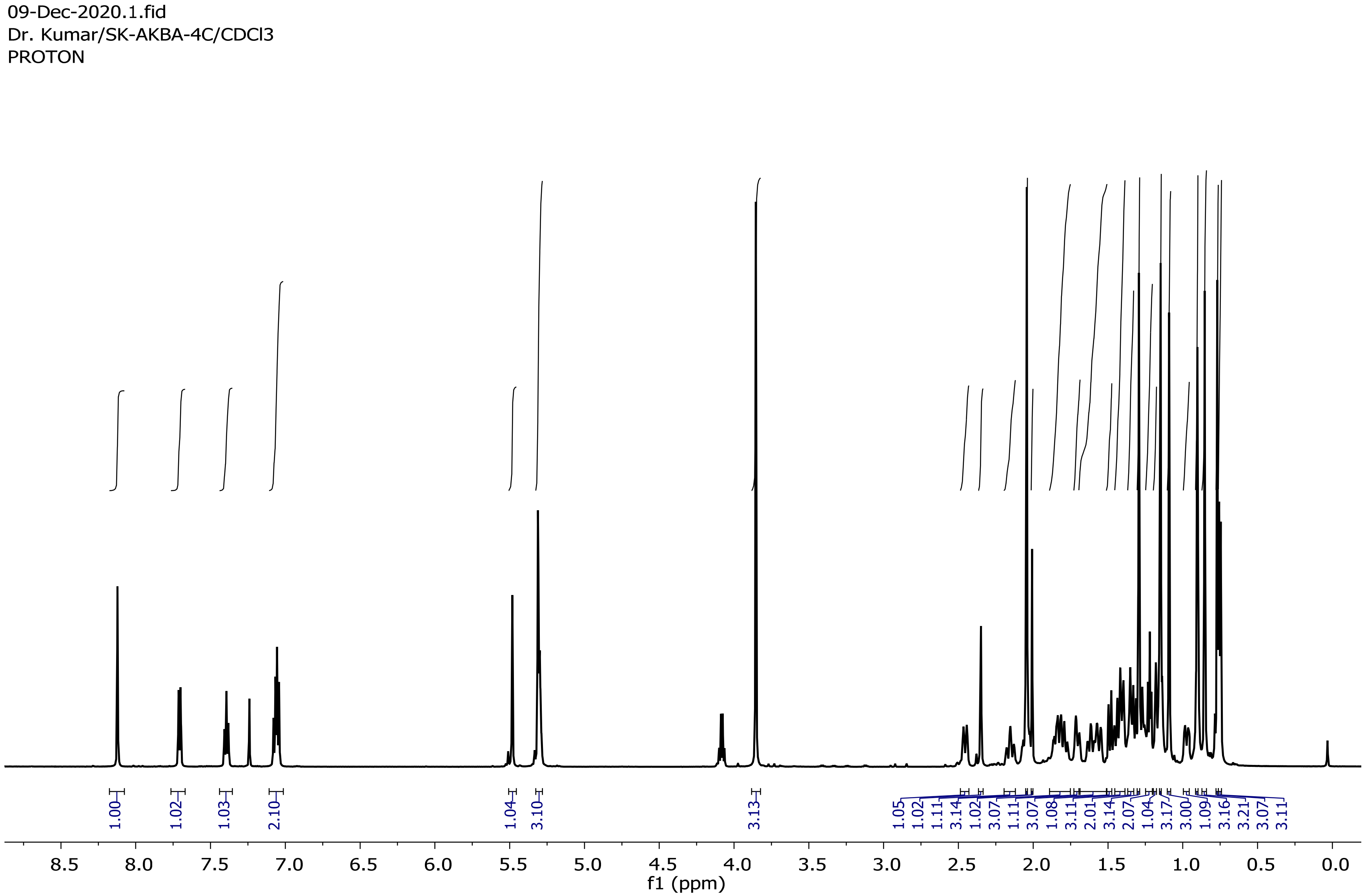

Figure S13: ^{1}H-NMR spectrum (600 MHz, $CDCl_3$) of compound **6c**

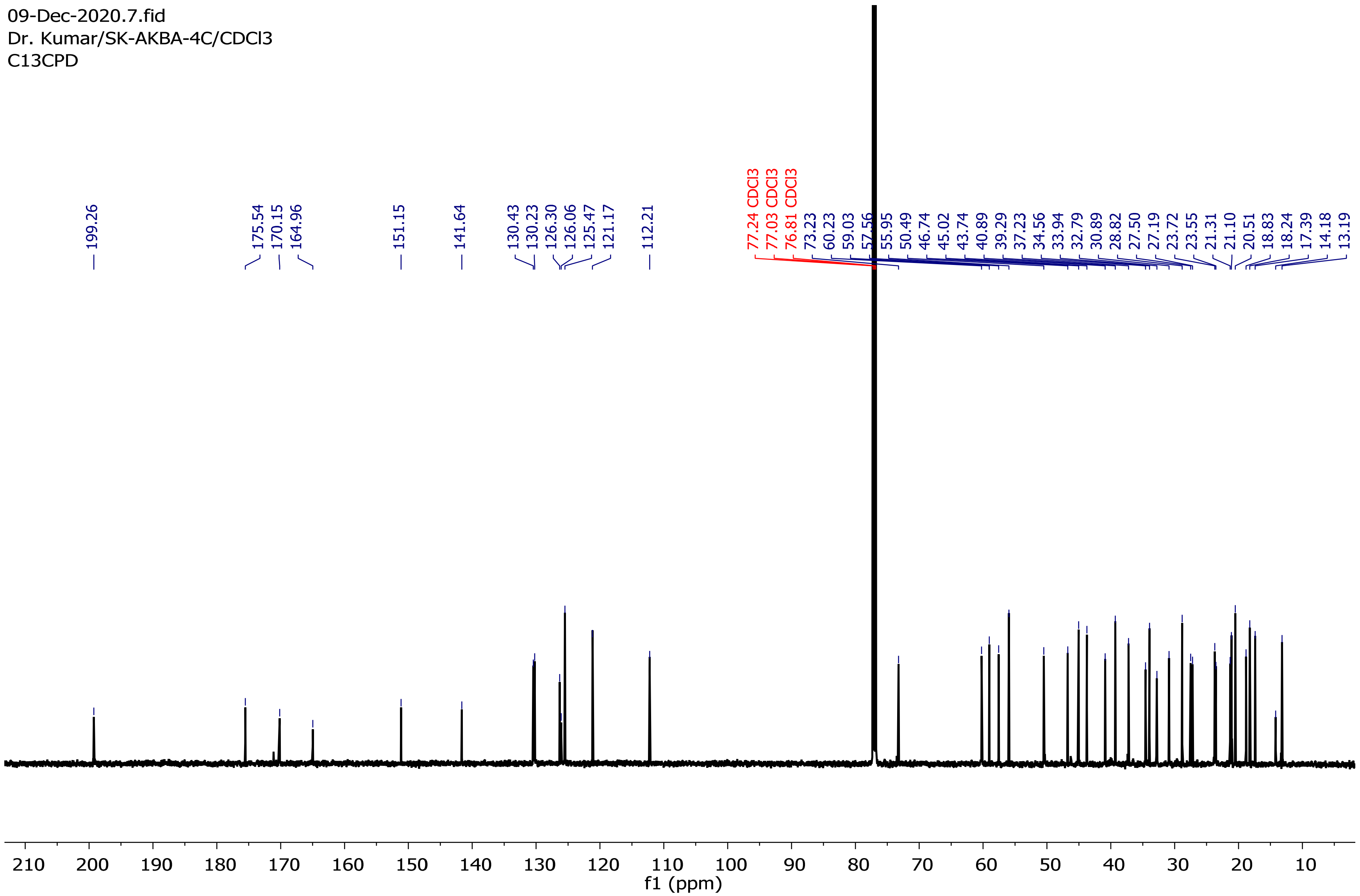

Figure S14: ^{13}C-NMR spectrum (150 MHz, $CDCl_3$) of compound **6c**

**Sample Name**	SK-AKBA-4C	**Position**	Vial 41	**Instrument Name**	Instrument 1
**User Name**		**Inj Vol**	2	**InjPosition**	
**Sample Type**	Sample	**IRM Calibration Status**	Success	**Data Filename**	SK-AKBA-4C_POS_01.d
**ACQ Method**	POSITIVE ION METHOD MS.m	**Comment**	SK	**Acquired Time**	11-Nov-20 3:02:18 PM

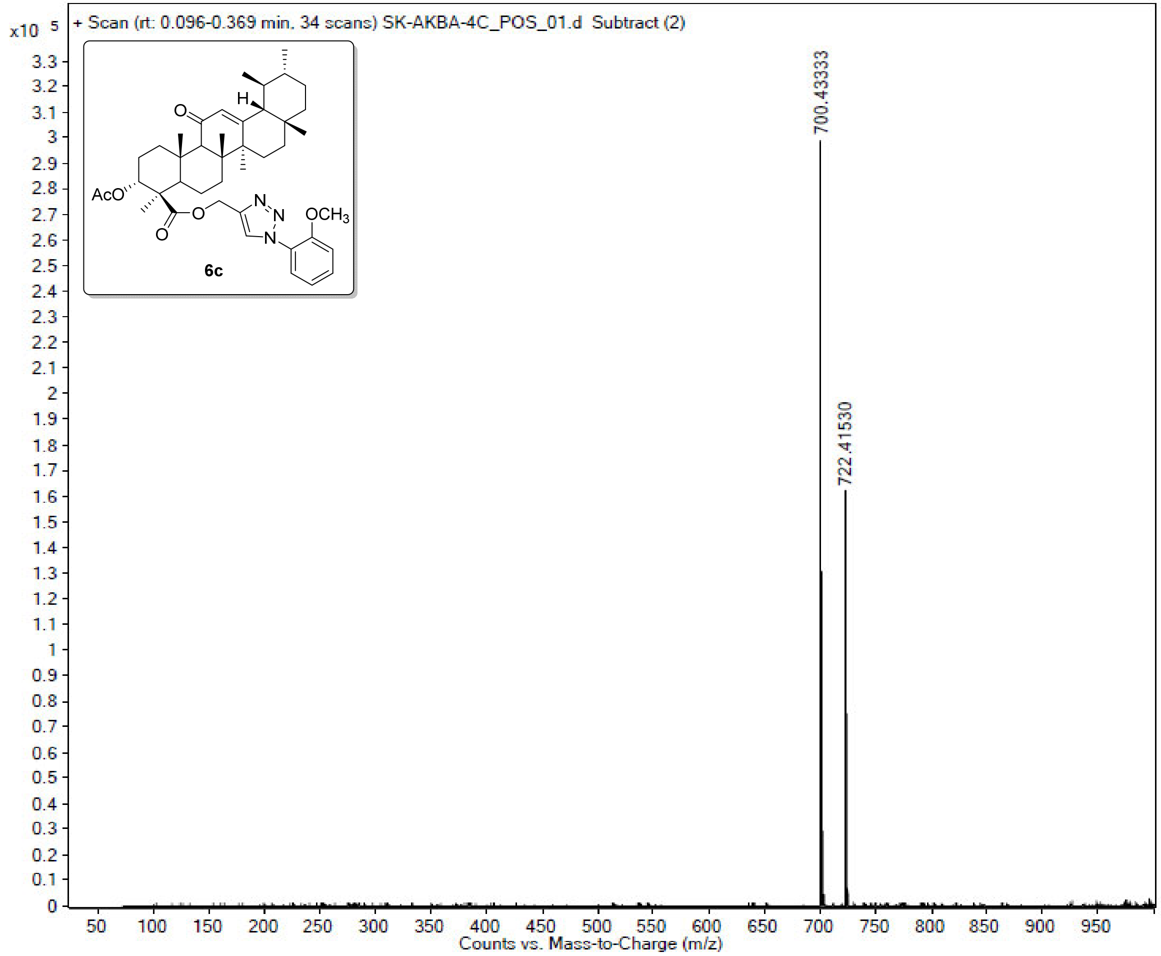

Figure S15: HRMS spectrum of compound **6c**

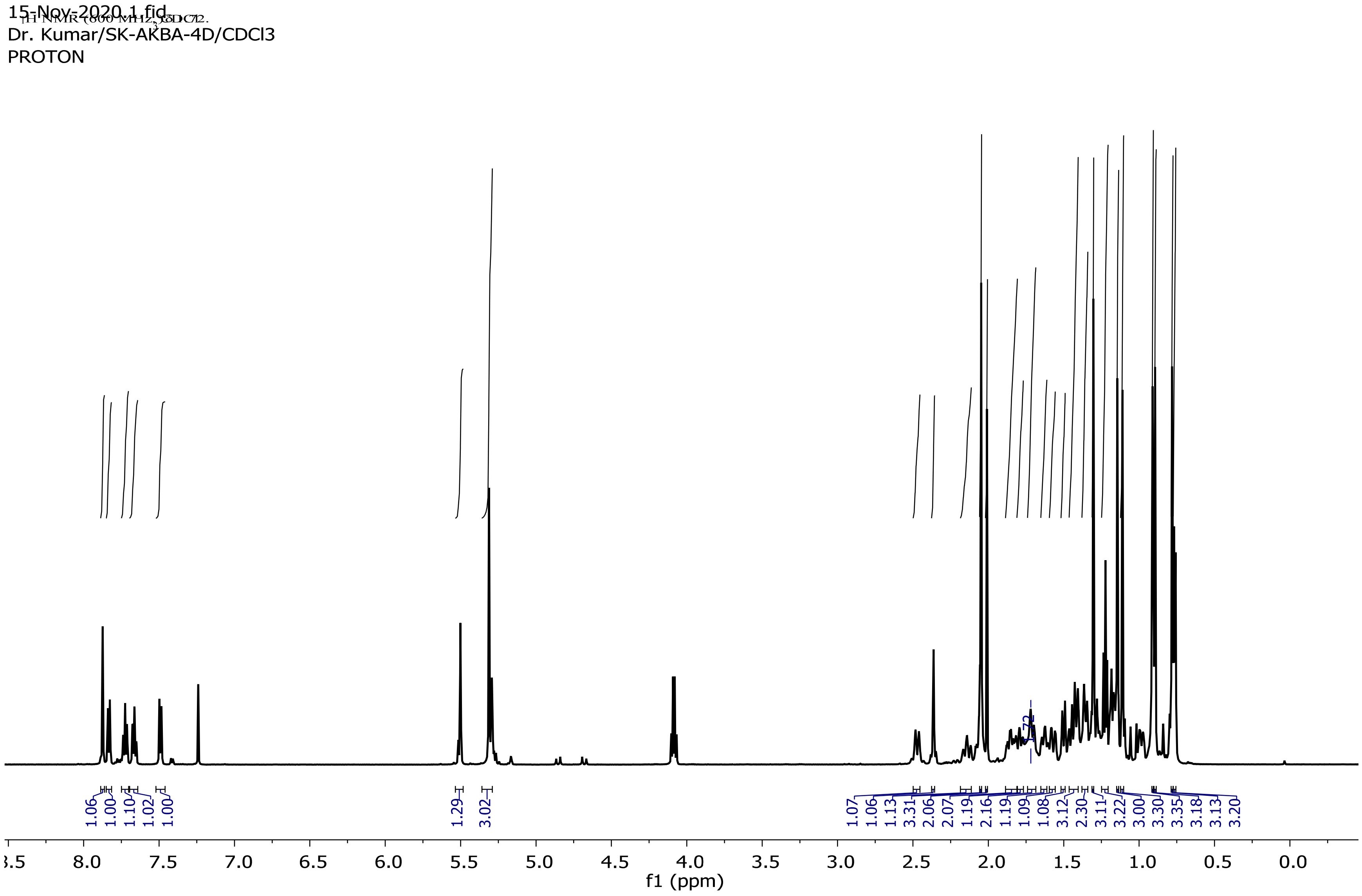

Figure S16: ^{1}H-NMR spectrum (600 MHz, $CDCl_3$) of compound **6d**

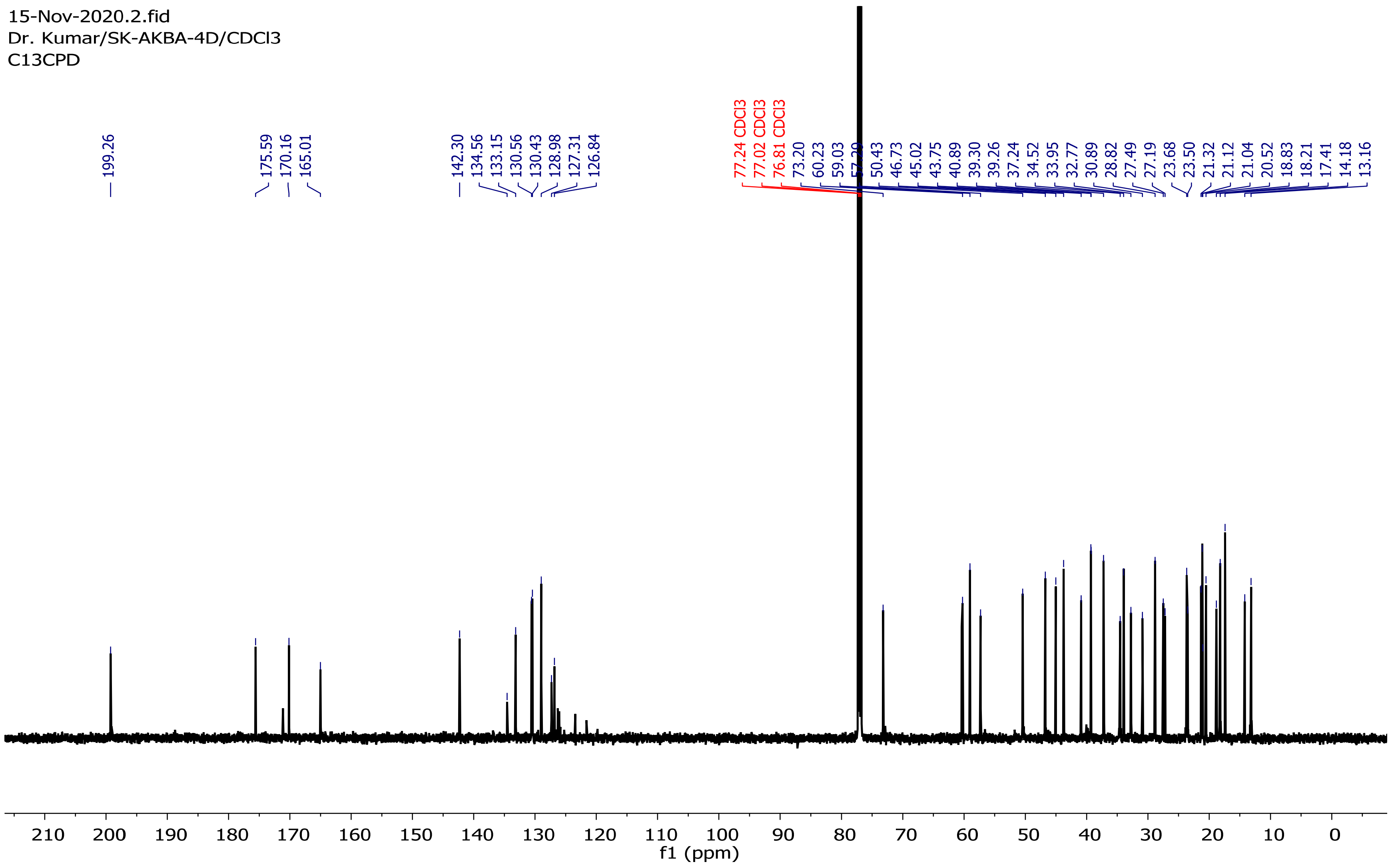

Figure S17: ^{13}C-NMR spectrum (150 MHz, $CDCl_3$) of compound **6d**

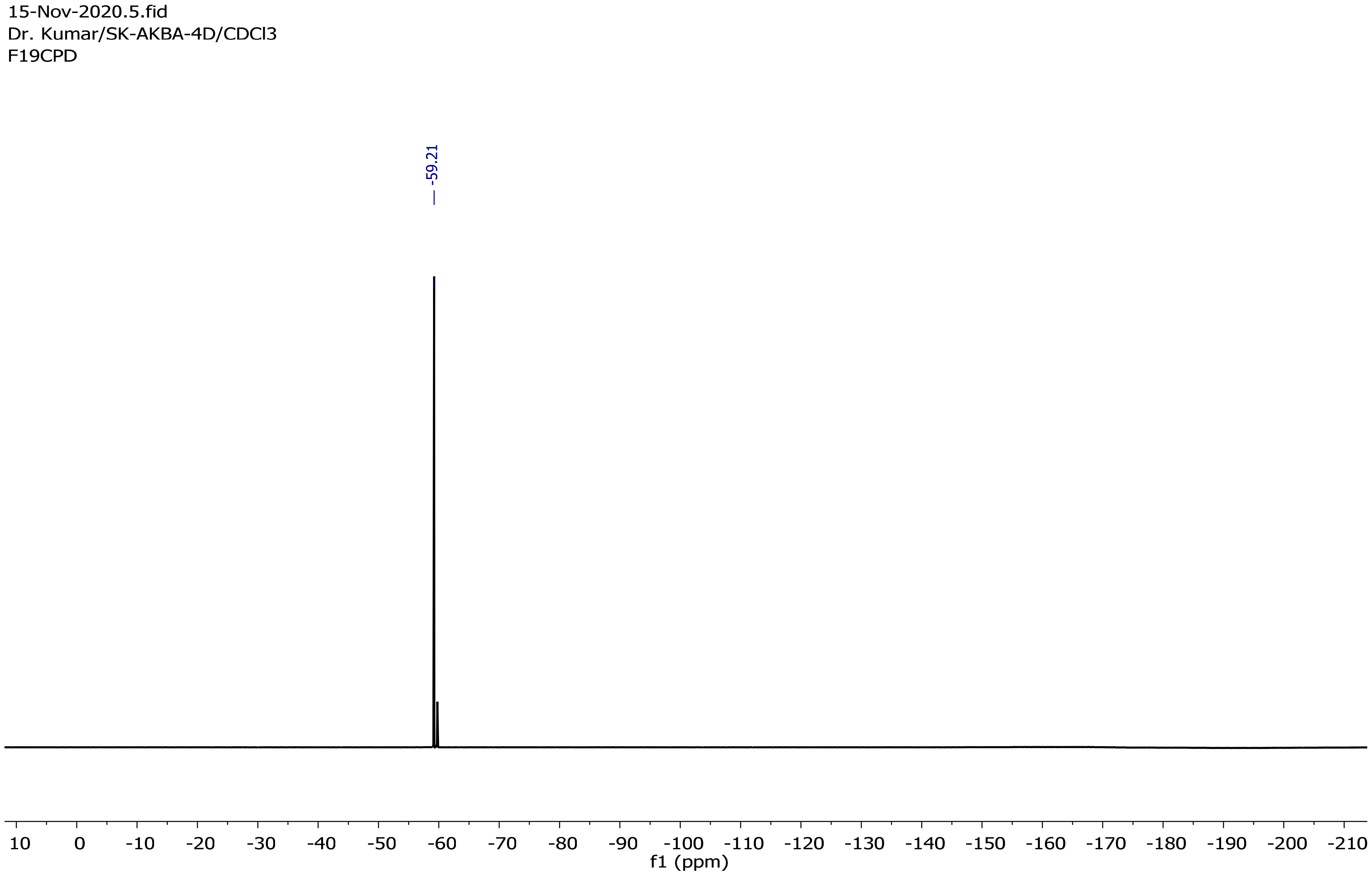

Figure S18: ^{19}F-NMR spectrum (564 MHz, $CDCl_3$) of compound **6d**

**Sample Name**	SK-AKBA-4D	**Position**	Vial 42	**Instrument Name**	Instrument 1
**User Name**		**Inj Vol**	2	**InjPosition**	
**Sample Type**	Sample	**IRM Calibration Status**	Success	**Data Filename**	SK-AKBA-4D_POS_01.d
**ACQ Method**	POSITIVE ION METHOD MS.m	**Comment**	SK	**Acquired Time**	11-Nov-20 3:07:56 PM

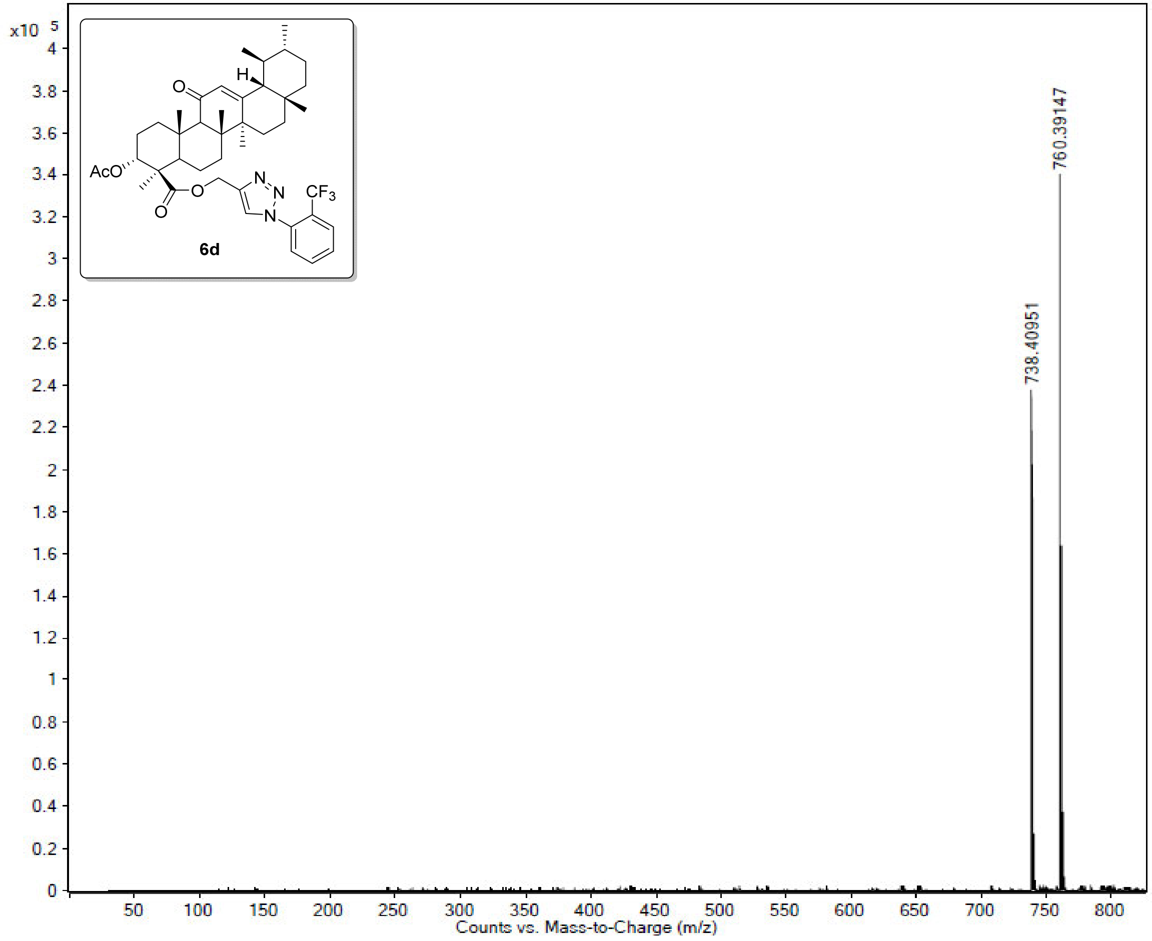

Figure S19: HRMS spectrum of compound **6d**

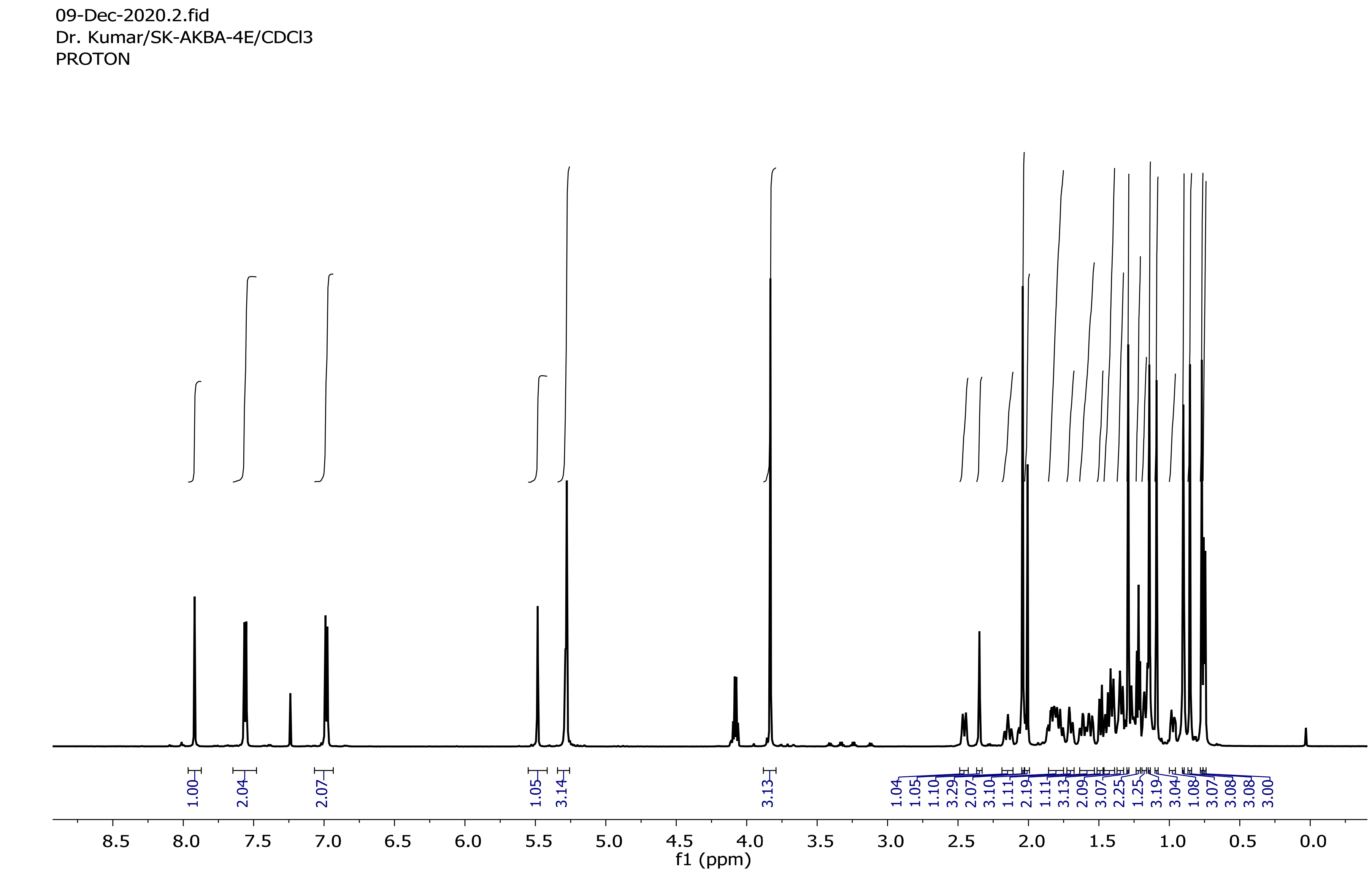

Figure S20: ^{1}H-NMR spectrum (600 MHz, $CDCl_3$) of compound **6e**

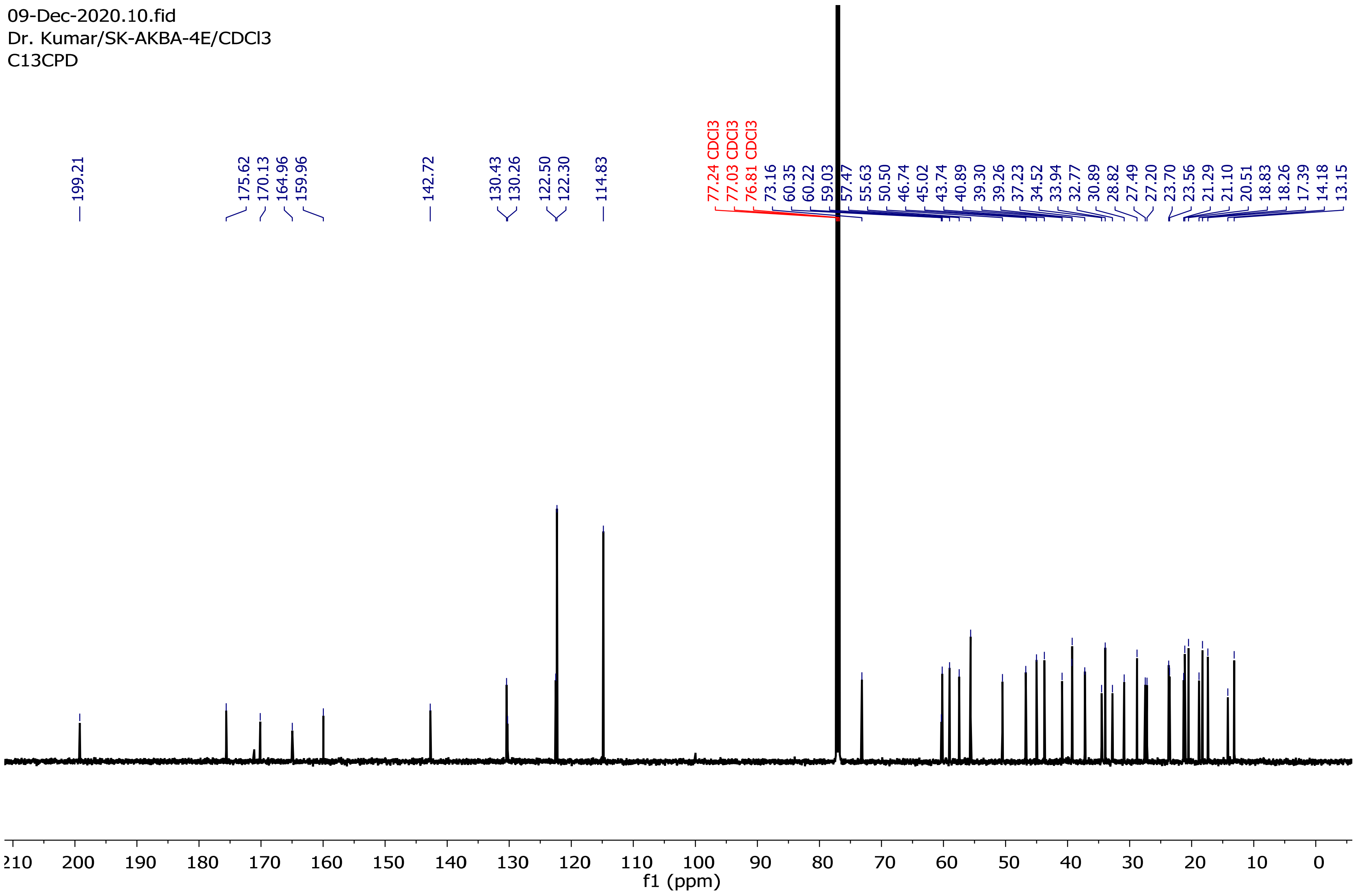

Figure S21: ^{13}C-NMR spectrum (150 MHz, $CDCl_3$) of compound **6e**

**Sample Name**	4E	**Position**	Vial 44	**Instrument Name**	Instrument 1
**User Name**		**Inj Vol**	2	**InjPosition**	
**Sample Type**	Sample	**IRM Calibration Status**	Success	**Data Filename**	4E_POS_01.d
**ACQ Method**	POSITIVE ION METHOD MS.m	**Comment**	SK	**Acquired Time**	20-Nov-20 6:38:30 PM

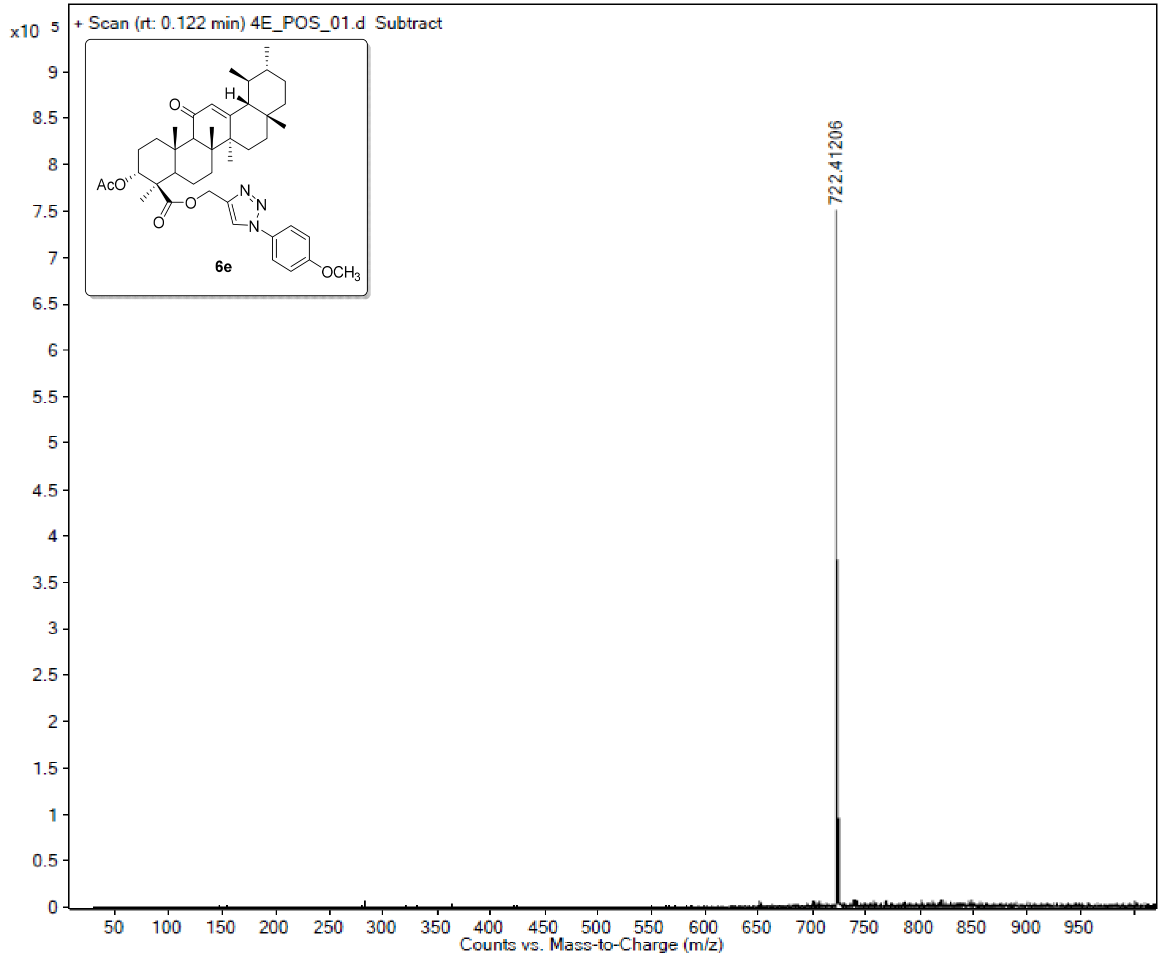

Figure S22: HRMS spectrum of compound **6e**

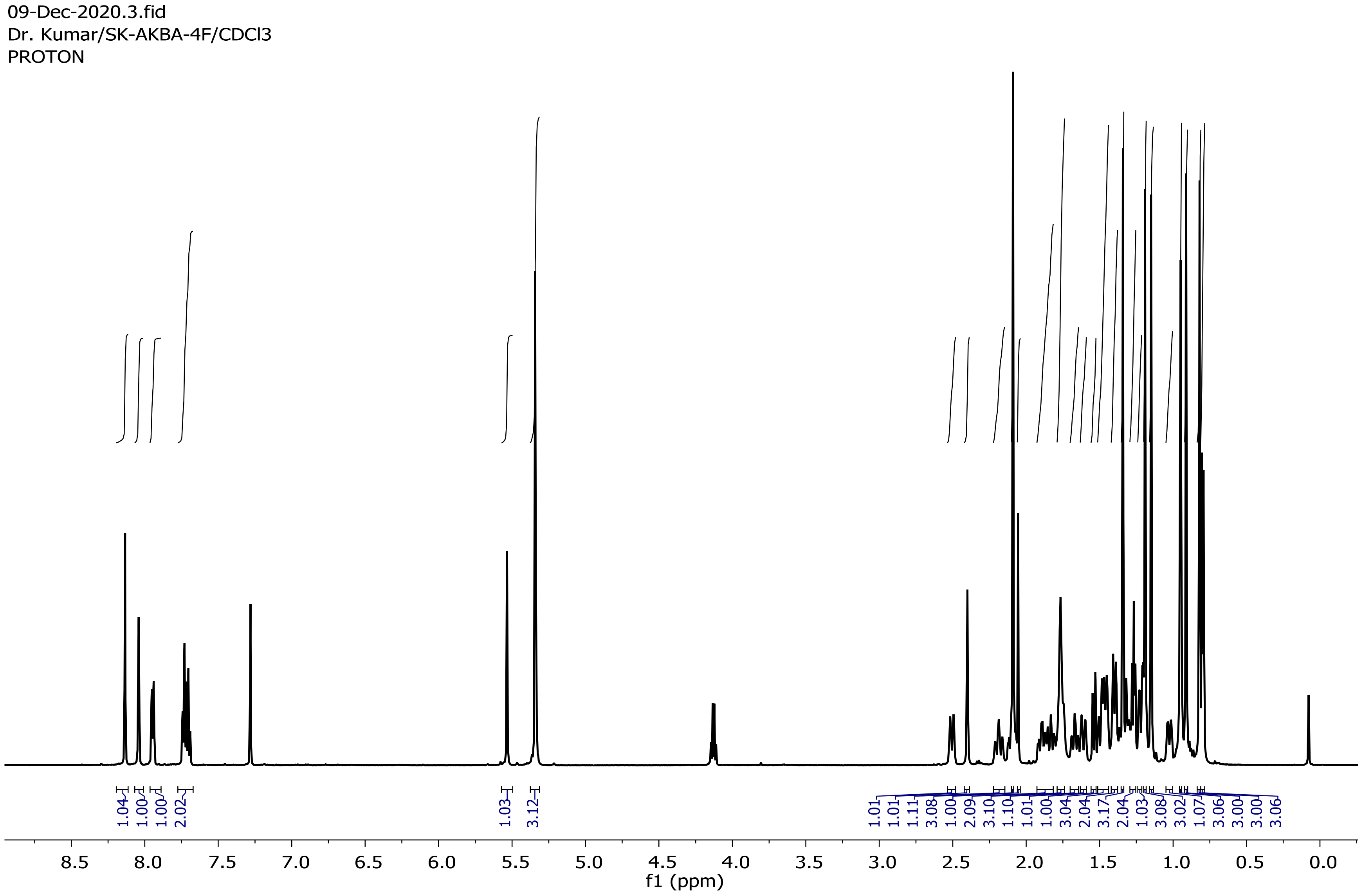

Figure S23: ^{1}H-NMR spectrum (600 MHz, $CDCl_3$) of compound **6f**

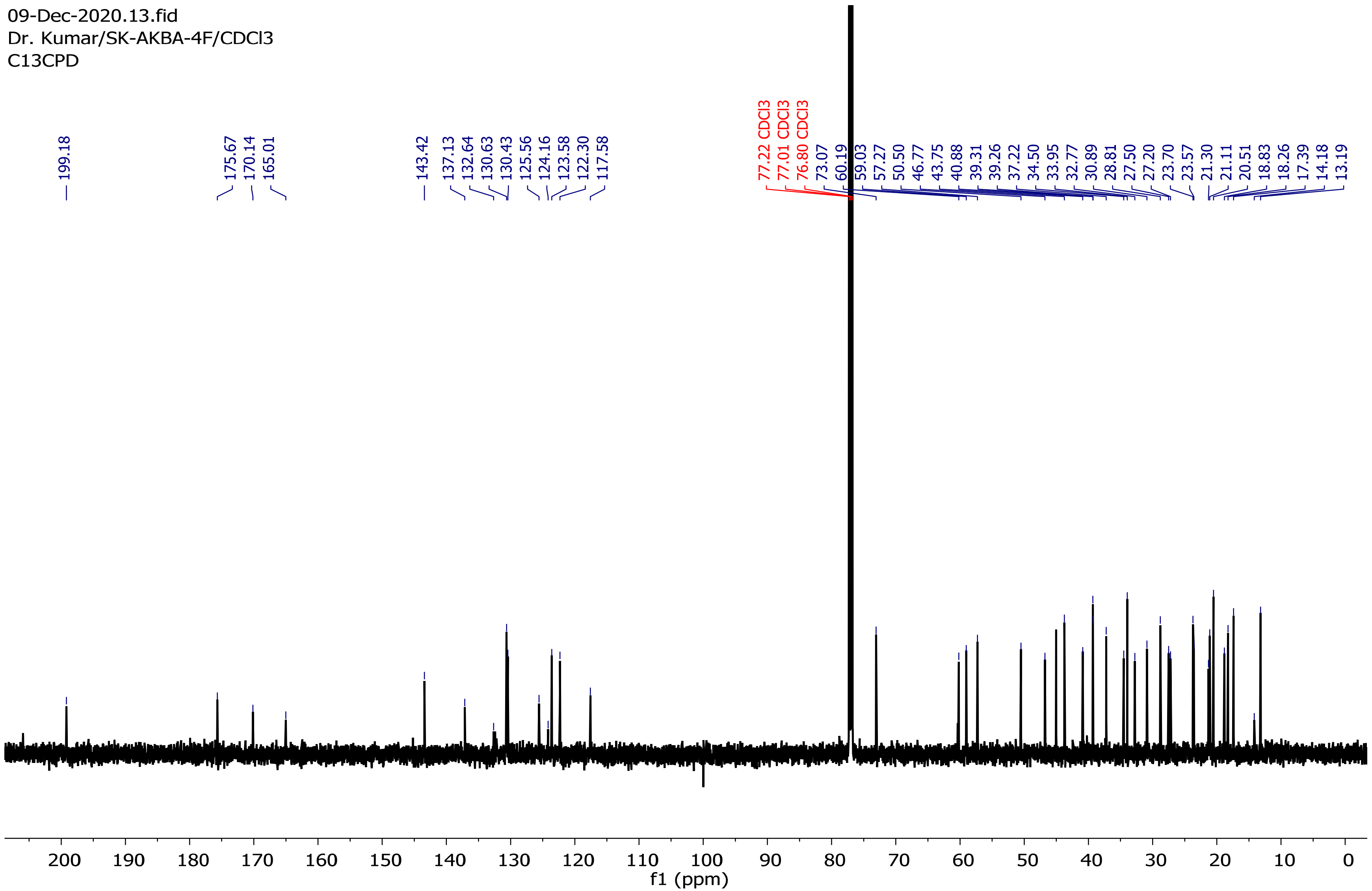

Figure S24: ^{13}C-NMR spectrum (150 MHz, $CDCl_3$) of compound **6f**

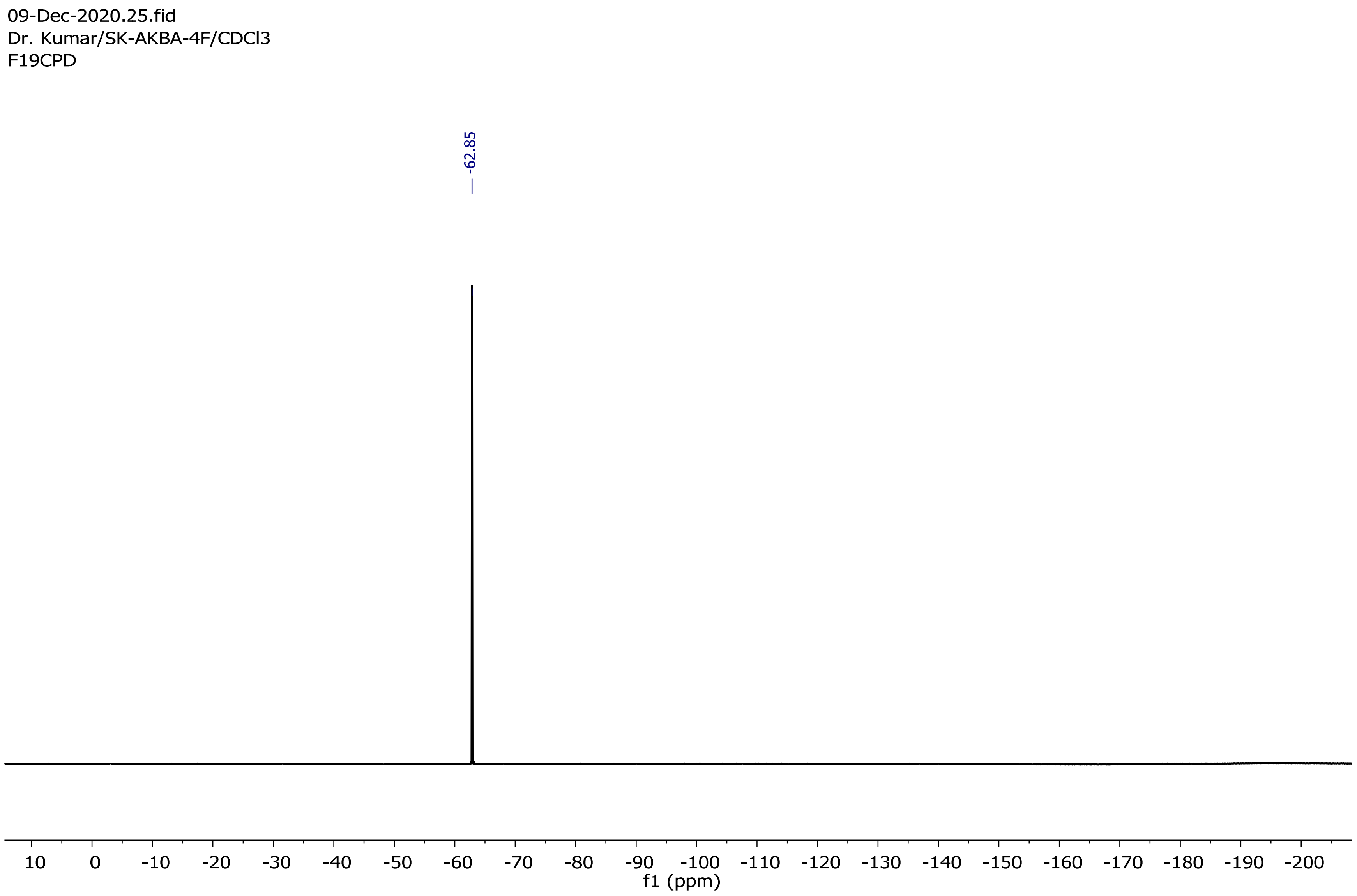

Figure S25: ^{19}F-NMR spectrum (564 MHz, $CDCl_3$) of compound **6f**

**Sample Name**	4F	**Position**	Vial 45	**Instrument Name**	Instrument 1
**User Name**		**Inj Vol**	2	**InjPosition**	
**Sample Type**	Sample	**IRM Calibration Status**	Success	**Data Filename**	4F_POS_01.d
**ACQ Method**	POSITIVE ION METHOD MS.m	**Comment**	SK	**Acquired Time**	20-Nov-20 6:44:00 PM

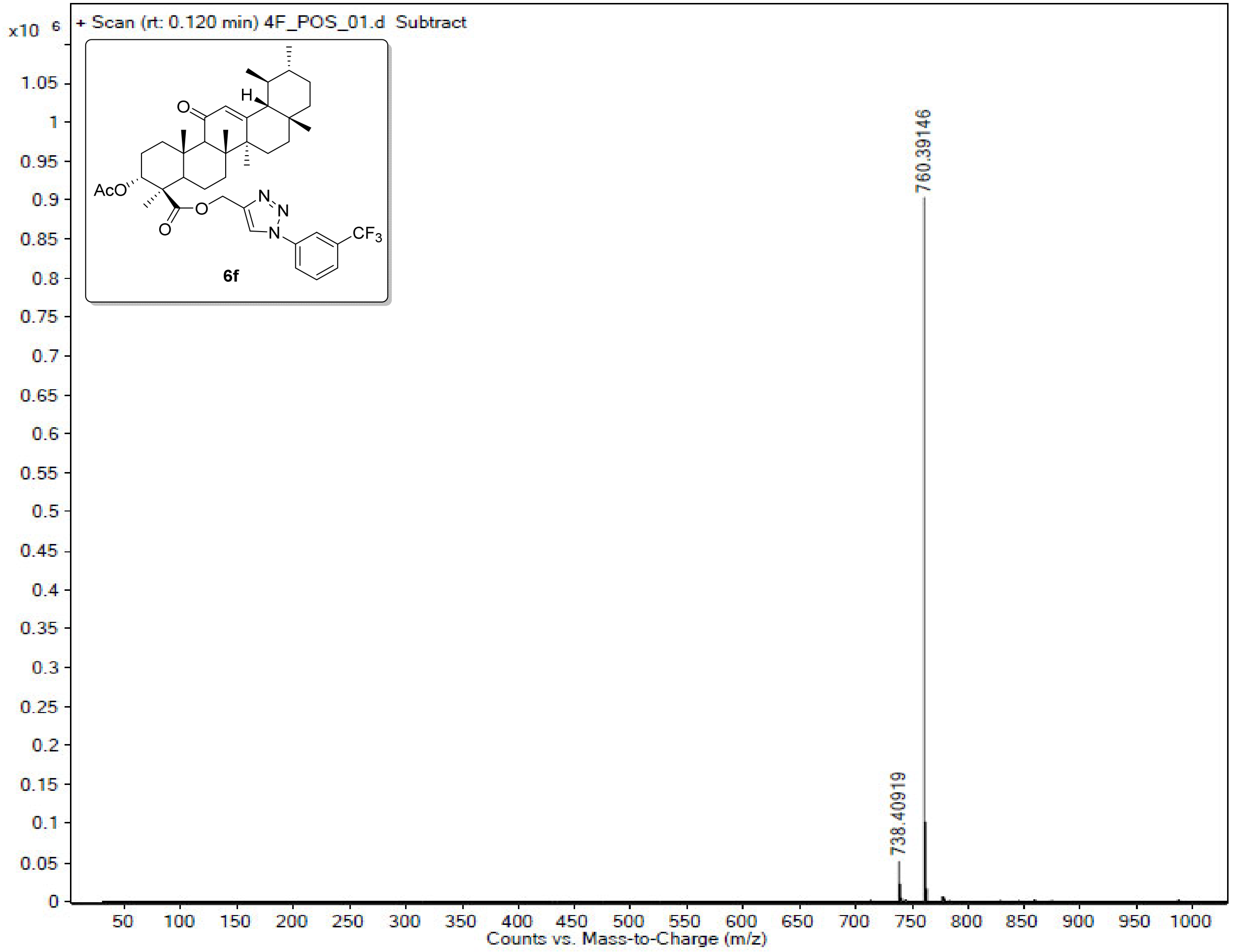

Figure S26: HRMS spectrum of compound **6f**

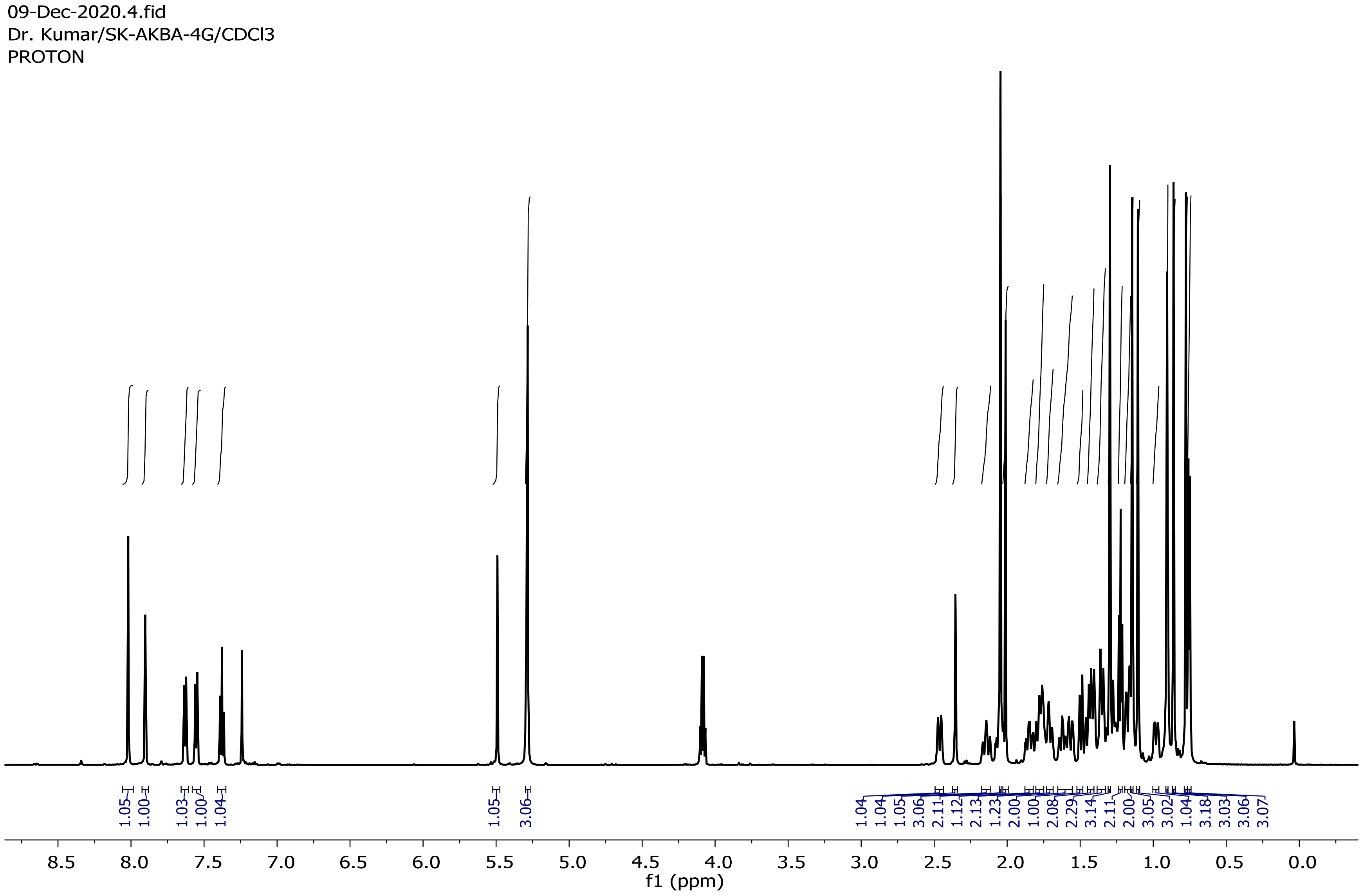

Figure S27: ^{1}H-NMR spectrum (600 MHz, $CDCl_3$) of compound **6g**

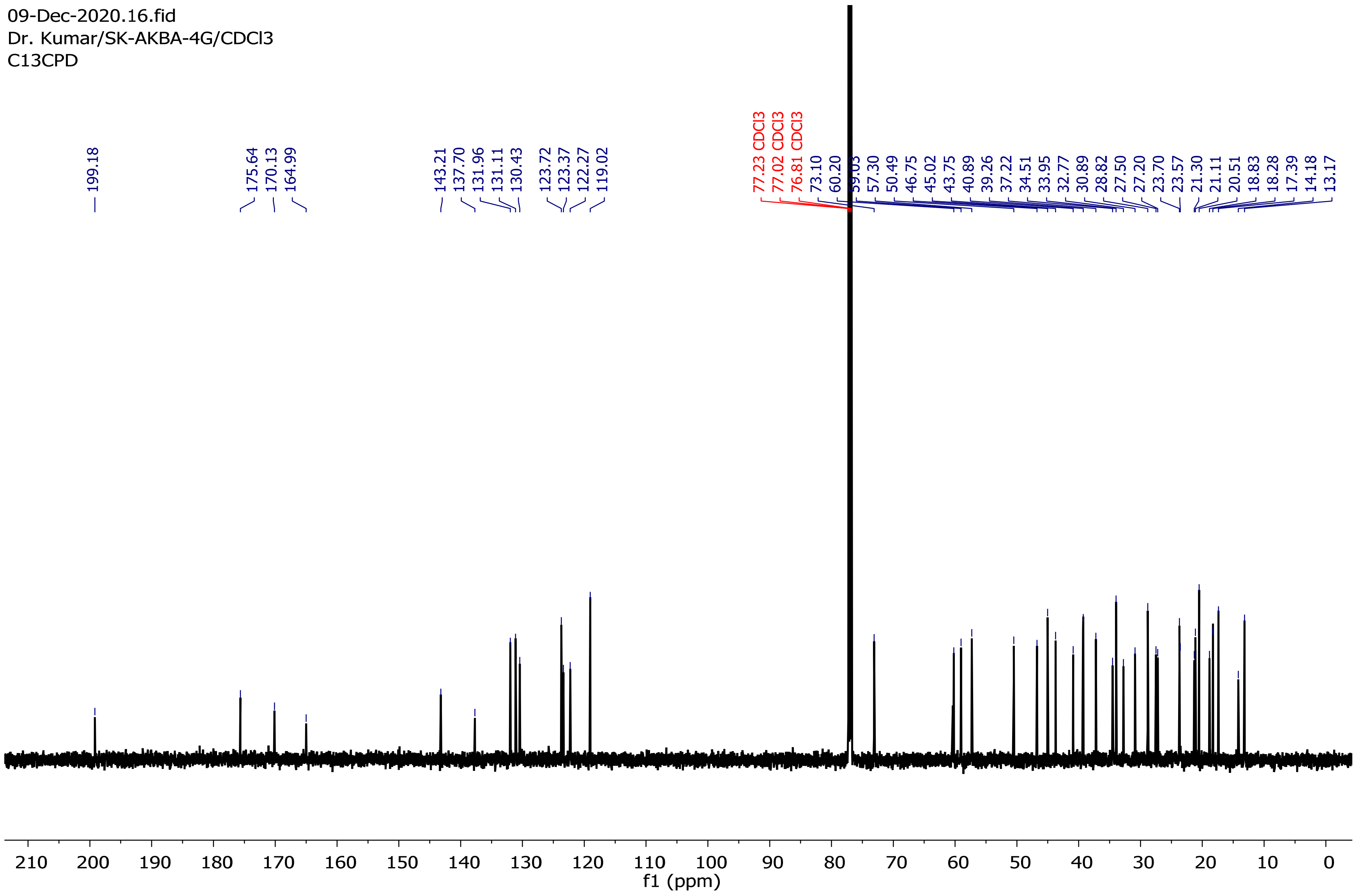

Figure S28: ^{13}C-NMR spectrum (150 MHz, $CDCl_3$) of compound **6g**

**Sample Name**	4G	**Position**	Vial 46	**Instrument Name**	Instrument 1
**User Name**		**Inj Vol**	2	**InjPosition**	
**Sample Type**	Sample	**IRM Calibration Status**	Success	**Data Filename**	4G_POS_01.d
**ACQ Method**	POSITIVE ION METHOD MS.m	**Comment**	SK	**Acquired Time**	20-Nov-20 6:49:35 PM

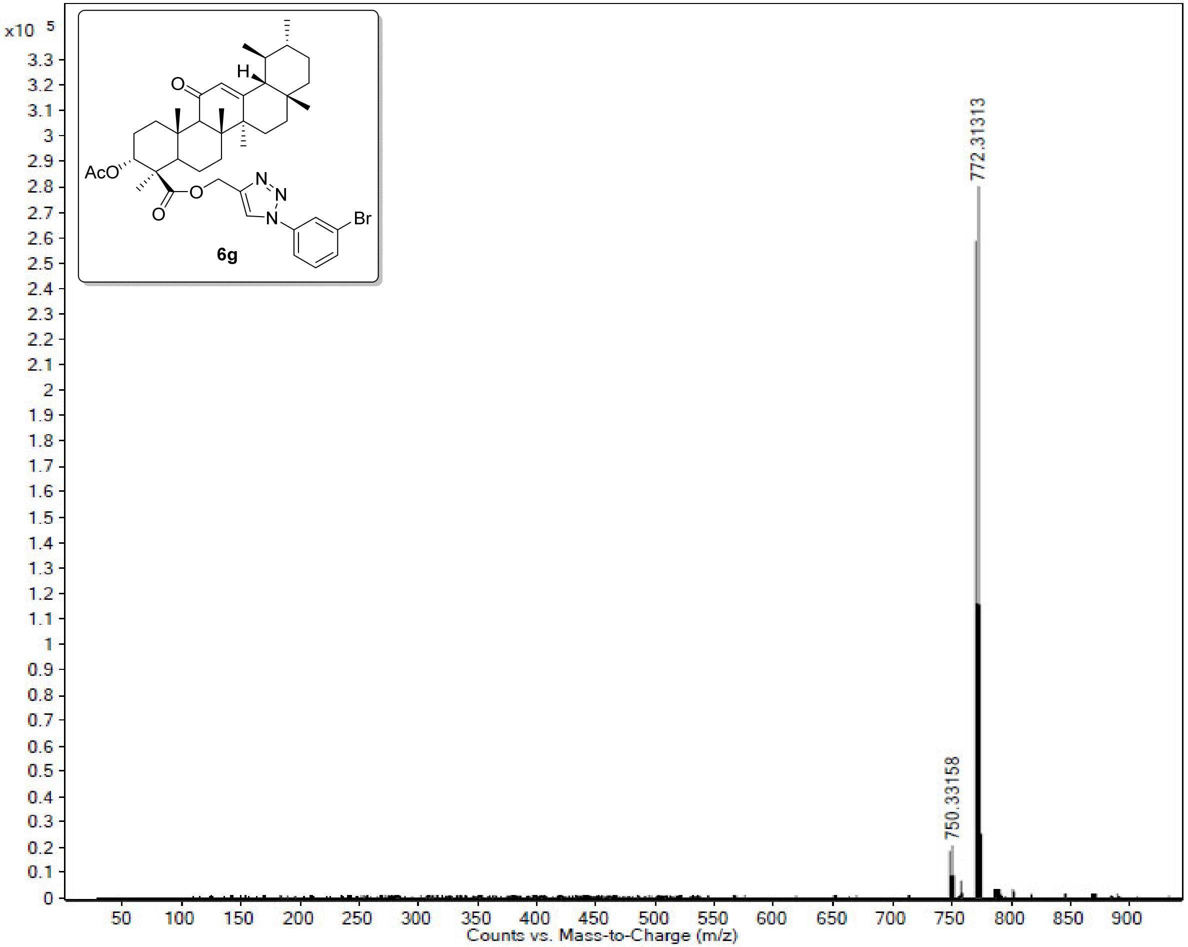

Figure S29: HRMS spectrum of compound **6g**

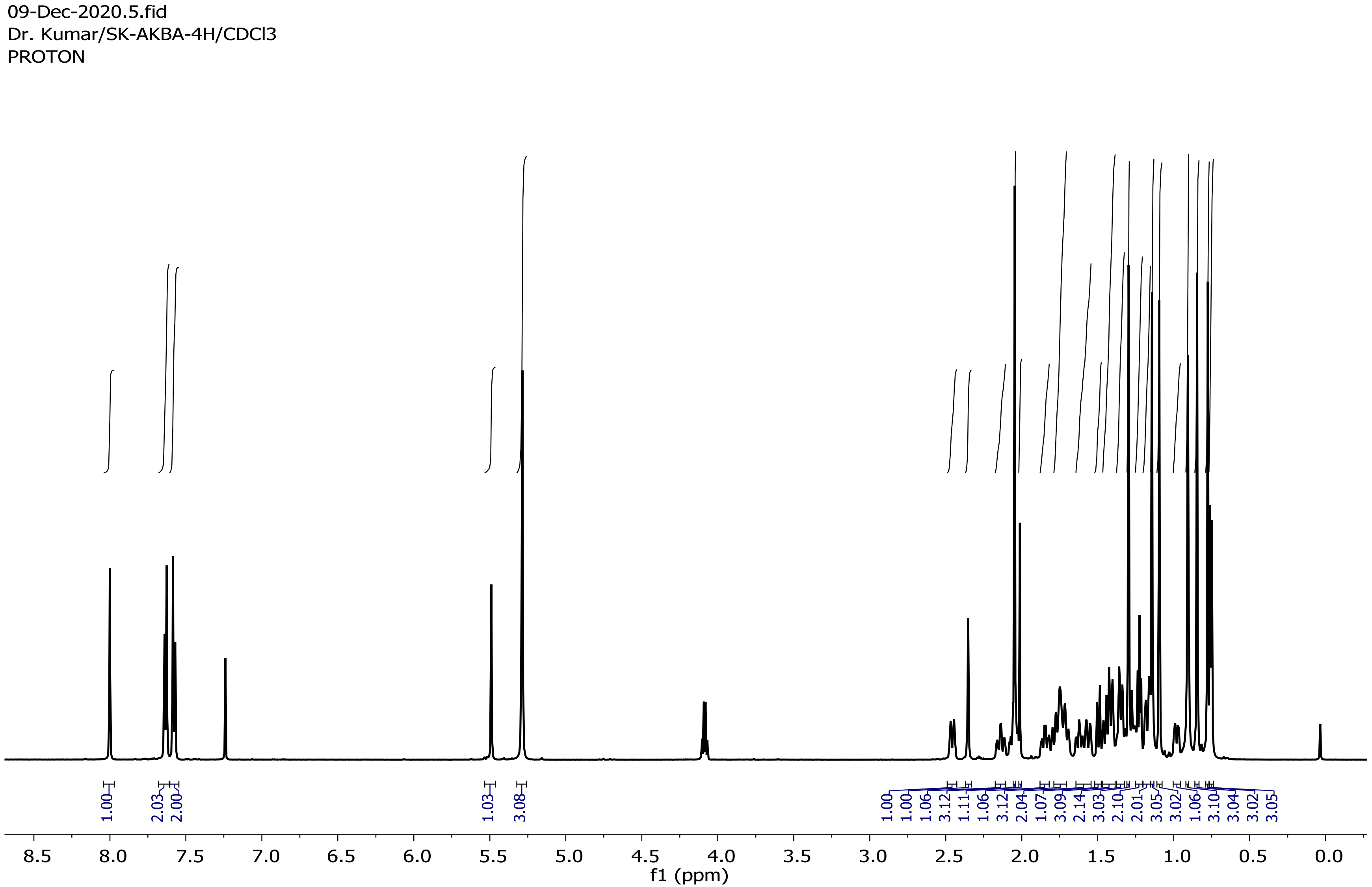

Figure S30: ^{1}H-NMR spectrum (600 MHz, $CDCl_3$) of compound **6h**

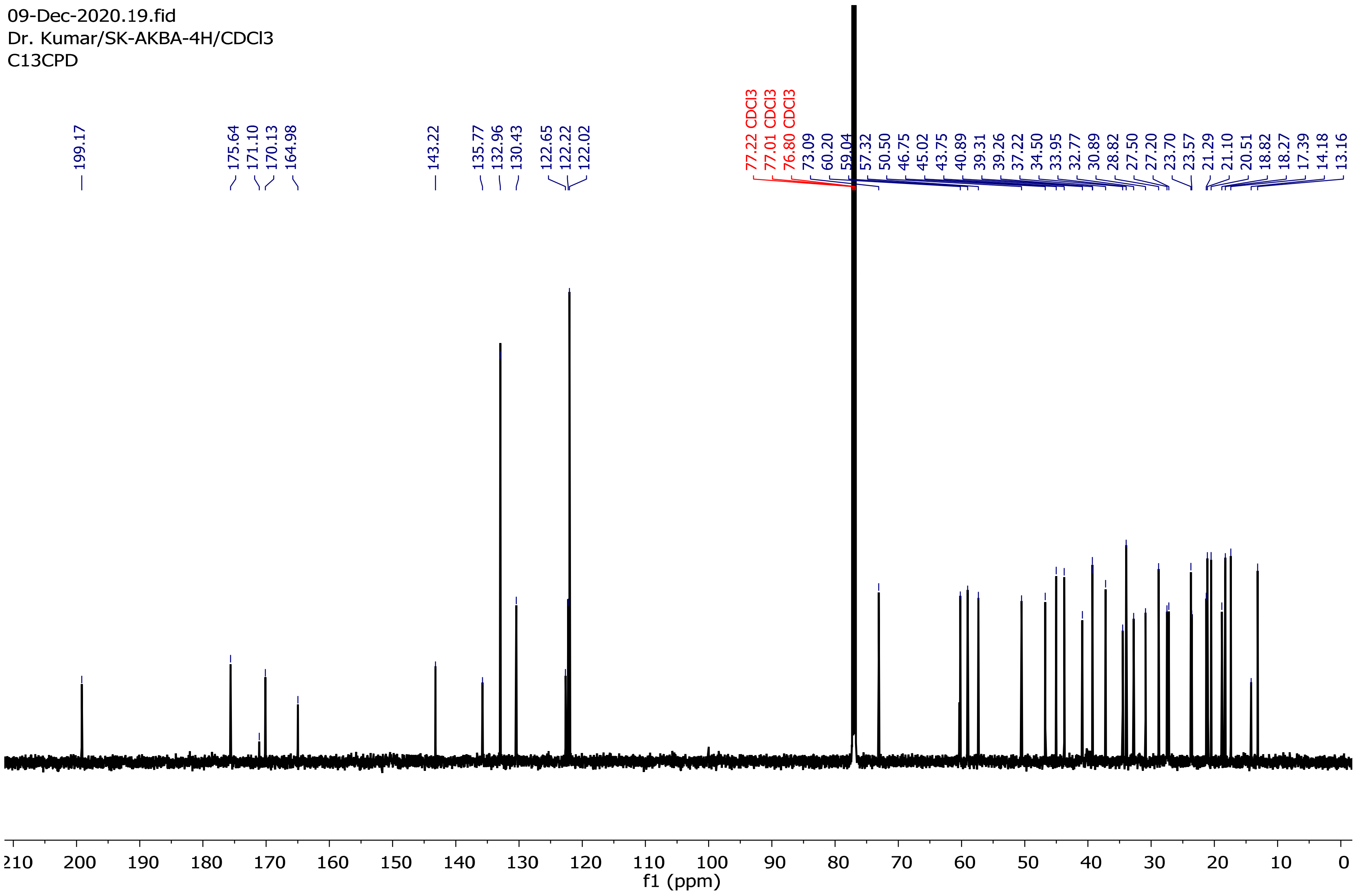

Figure S31: ^{13}C-NMR spectrum (150 MHz, $CDCl_3$) of compound **6h**

**Sample Name**	4H	**Position**	Vial 47	**Instrument Name**	Instrument 1
**User Name**		**Inj Vol**	2	**InjPosition**	
**Sample Type**	Sample	**IRM Calibration Status**	Success	**Data Filename**	4H_POS_02.d
**ACQ Method**	POSITIVE ION METHOD MS.m	**Comment**	SK	**Acquired Time**	20-Nov-20 6:55:10 PM

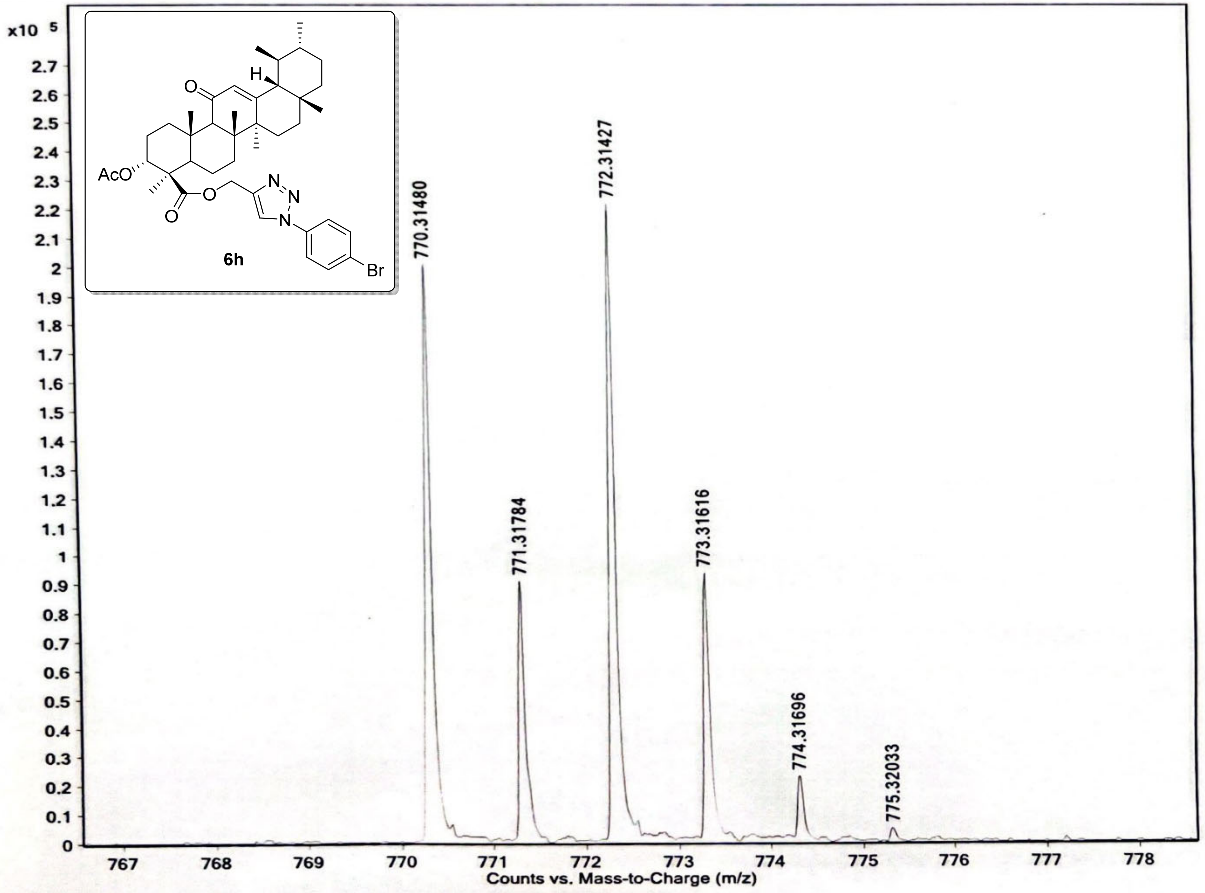

Figure S32: HRMS spectrum of compound **6h**

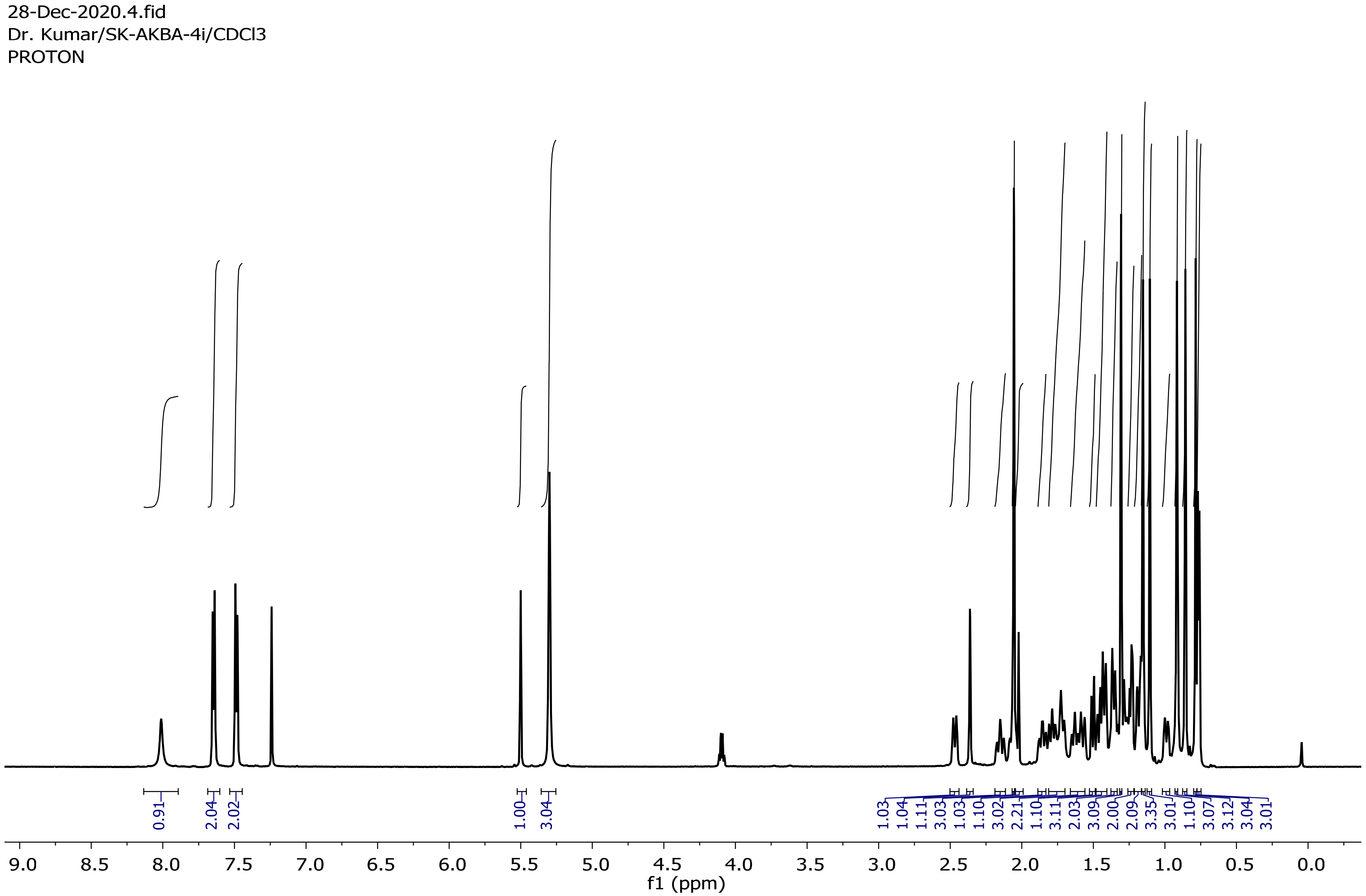

Figure S33: ^{1}H-NMR spectrum (600 MHz, $CDCl_3$) of compound **6i**

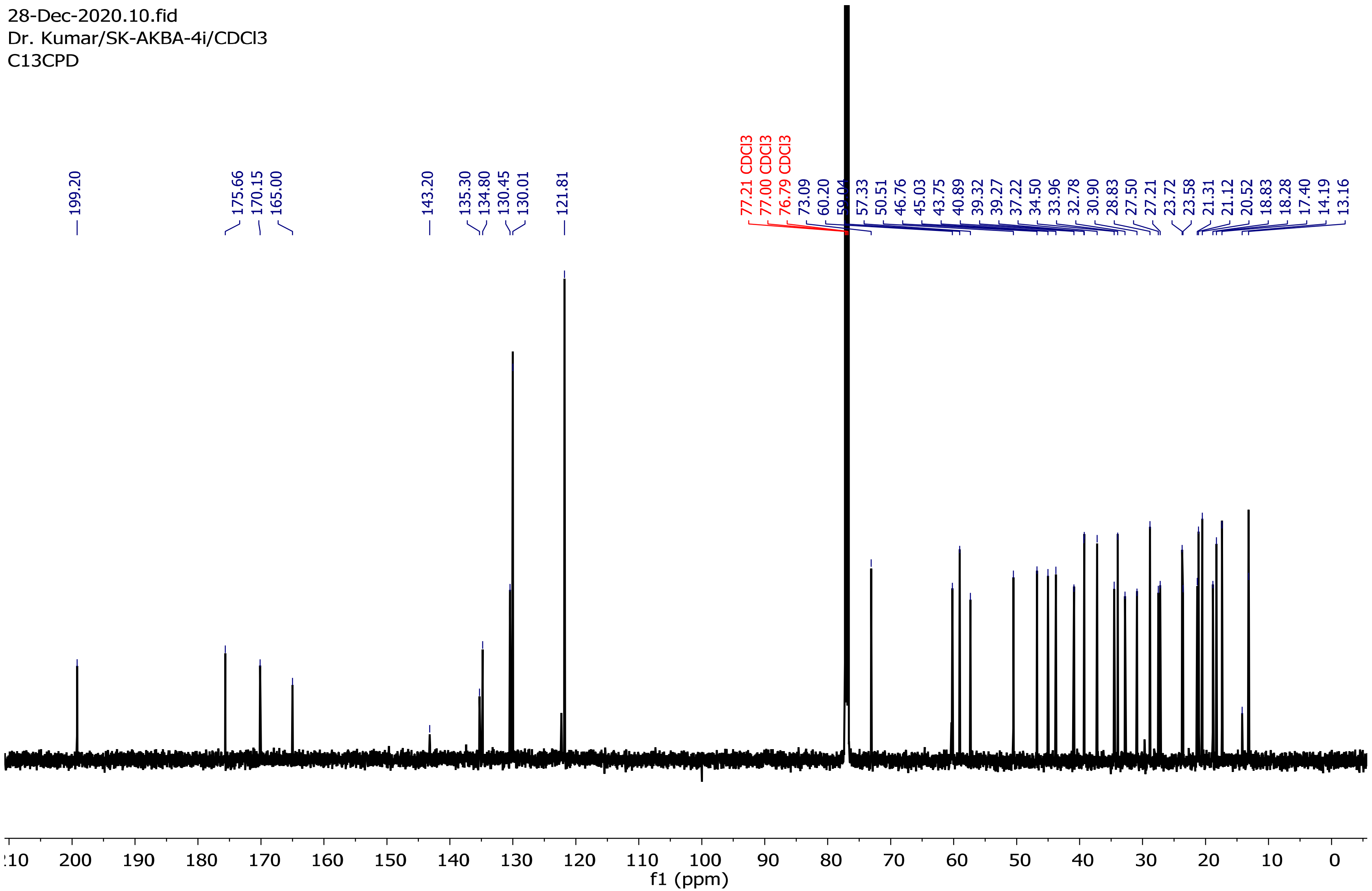

Figure S34: ^{13}C-NMR spectrum (150 MHz, $CDCl_3$) of compound **6i**

**Sample Name**	SK-AKBA-4I	**Position**	Vial 1	**Instrument Name**	Instrument 1
**User Name**		**Inj Vol**	2	**InjPosition**	
**Sample Type**	Sample	**IRM Calibration Status**	Success	**Data Filename**	SK-AKBA-4I_POS_01.d
**ACQ Method**	POSITIVE ION METHOD MS.m	**Comment**	SK	**Acquired Time**	28-Dec-20 2:32:25 PM

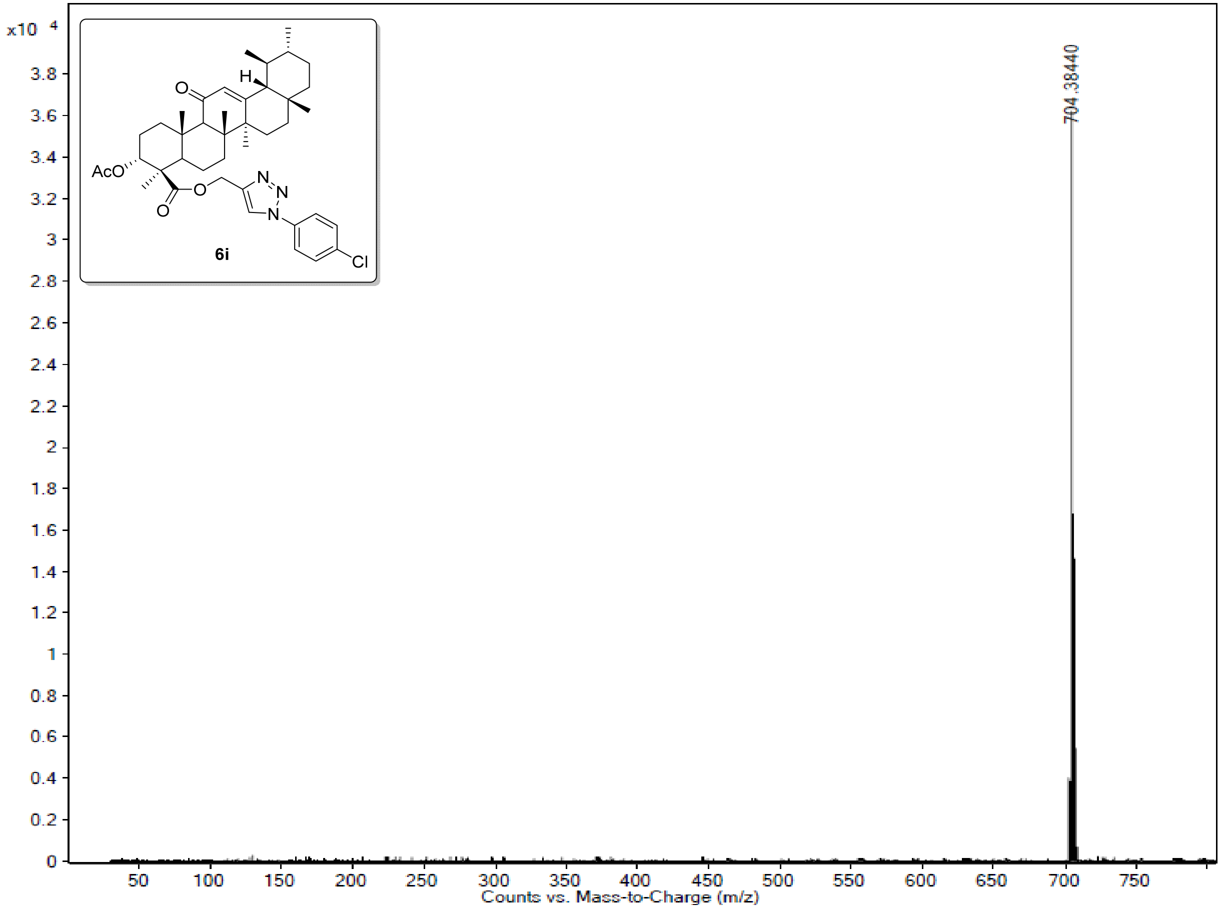

Figure S35: HRMS spectrum of compound **6i**

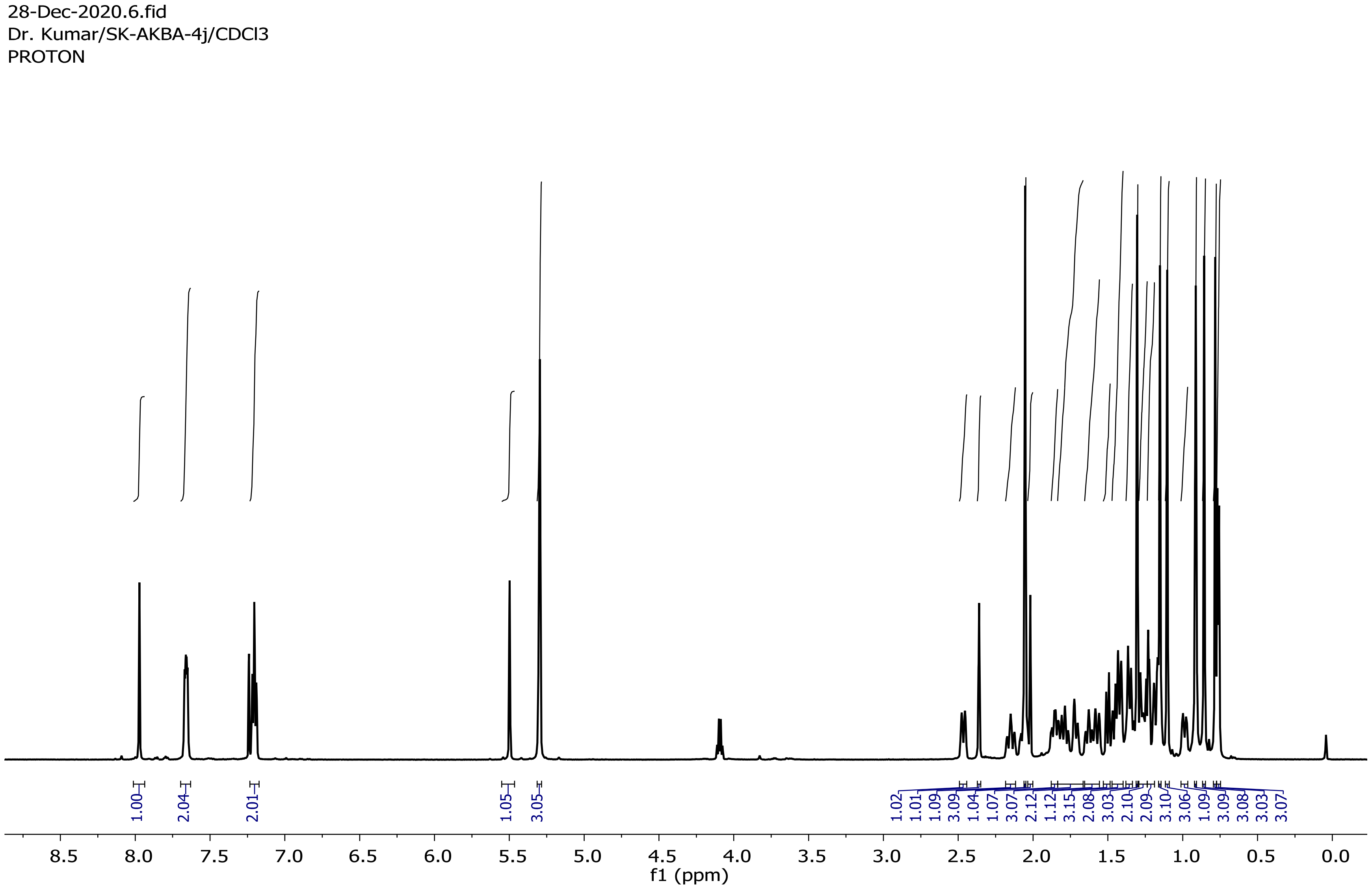

Figure S36: ^{1}H-NMR spectrum (600 MHz, $CDCl_3$) of compound **6j**

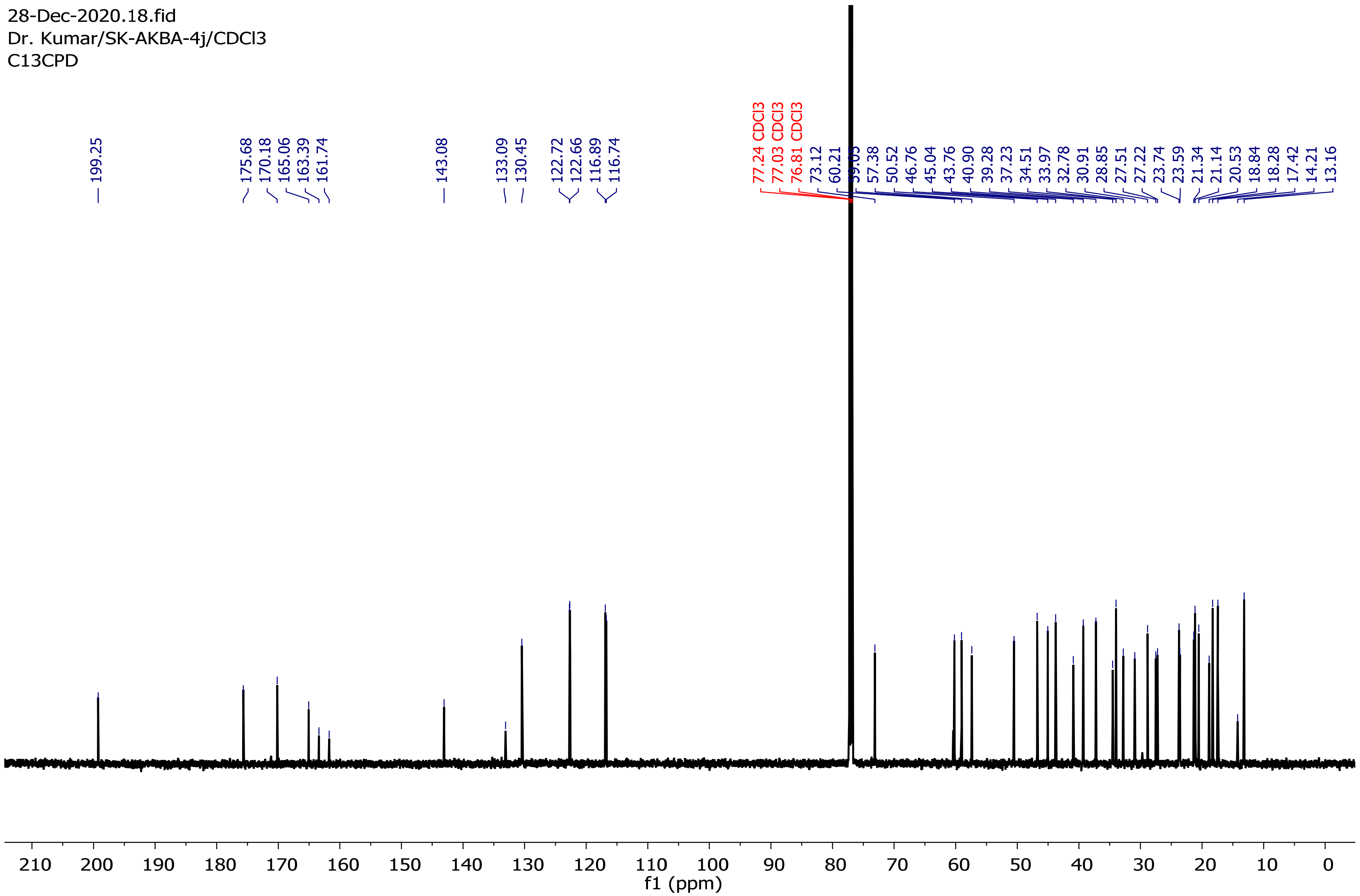

Figure S37: ^{13}C-NMR spectrum (150 MHz, $CDCl_3$) of compound **6j**

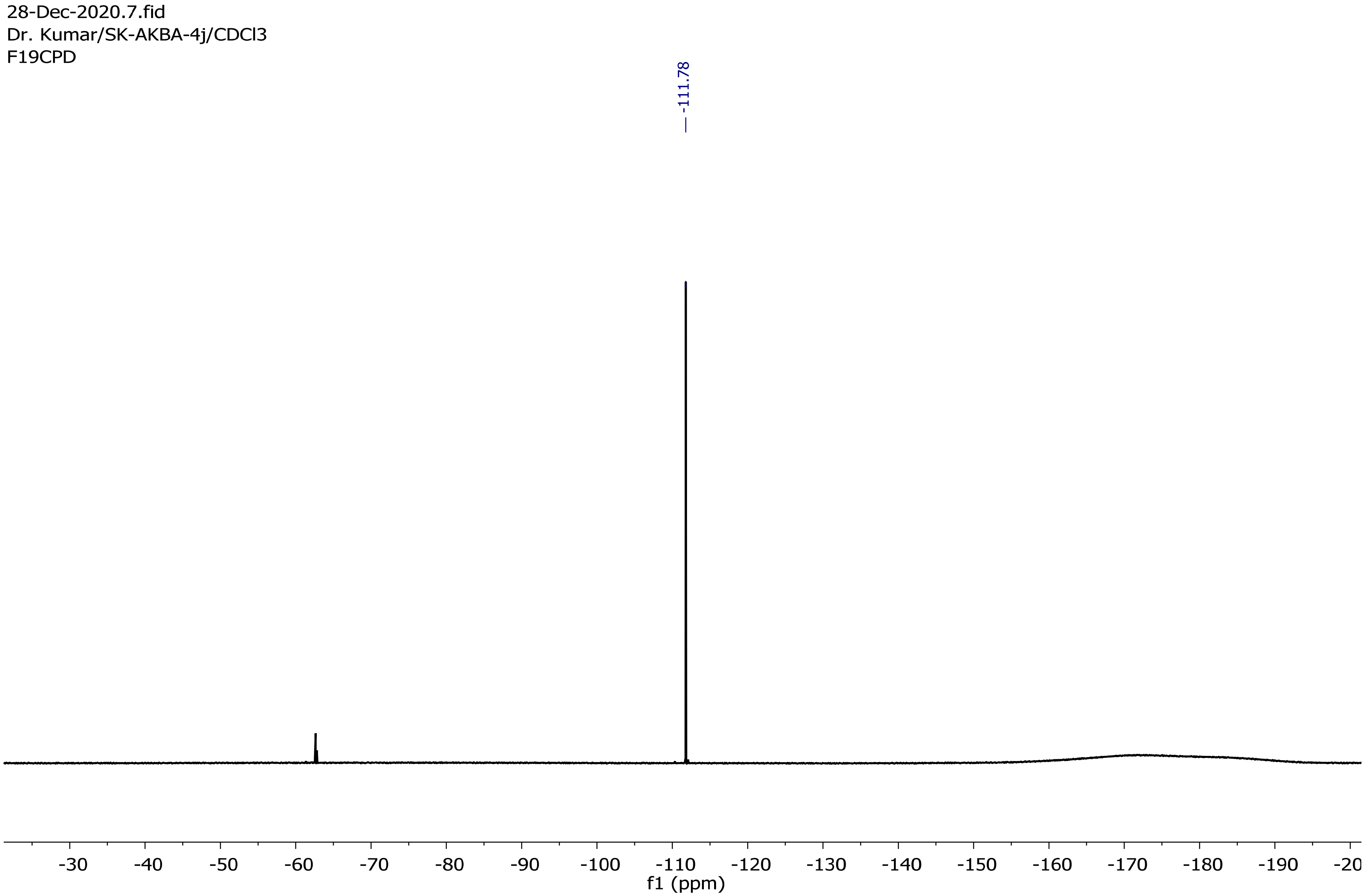

Figure S38: ^{19}F-NMR spectrum (564 MHz, $CDCl_3$) of compound **6j**

**Sample Name**	SK-AKBA-4J	**Position**	Vial 2	**Instrument Name**	Instrument 1
**User Name**		**Inj Vol**	2	**InjPosition**	
**Sample Type**	Sample	**IRM Calibration Status**	Success	**Data Filename**	SK-AKBA-4J_POS_01.d
**ACQ Method**	POSITIVE ION METHOD MS.m	**Comment**	SK	**Acquired Time**	28-Dec-20 2:38:01 PM

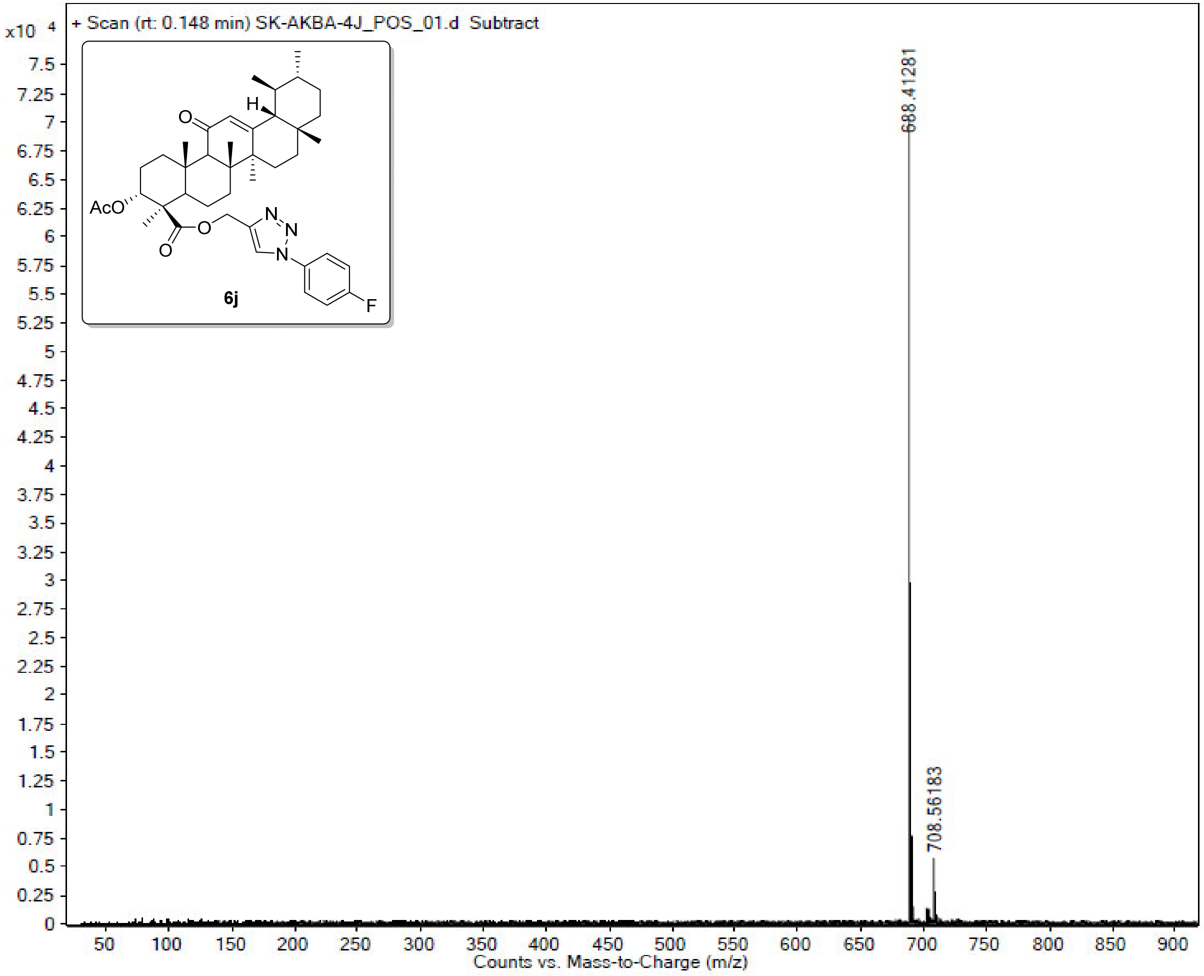

Figure S39: HRMS spectrum of compound **6j**

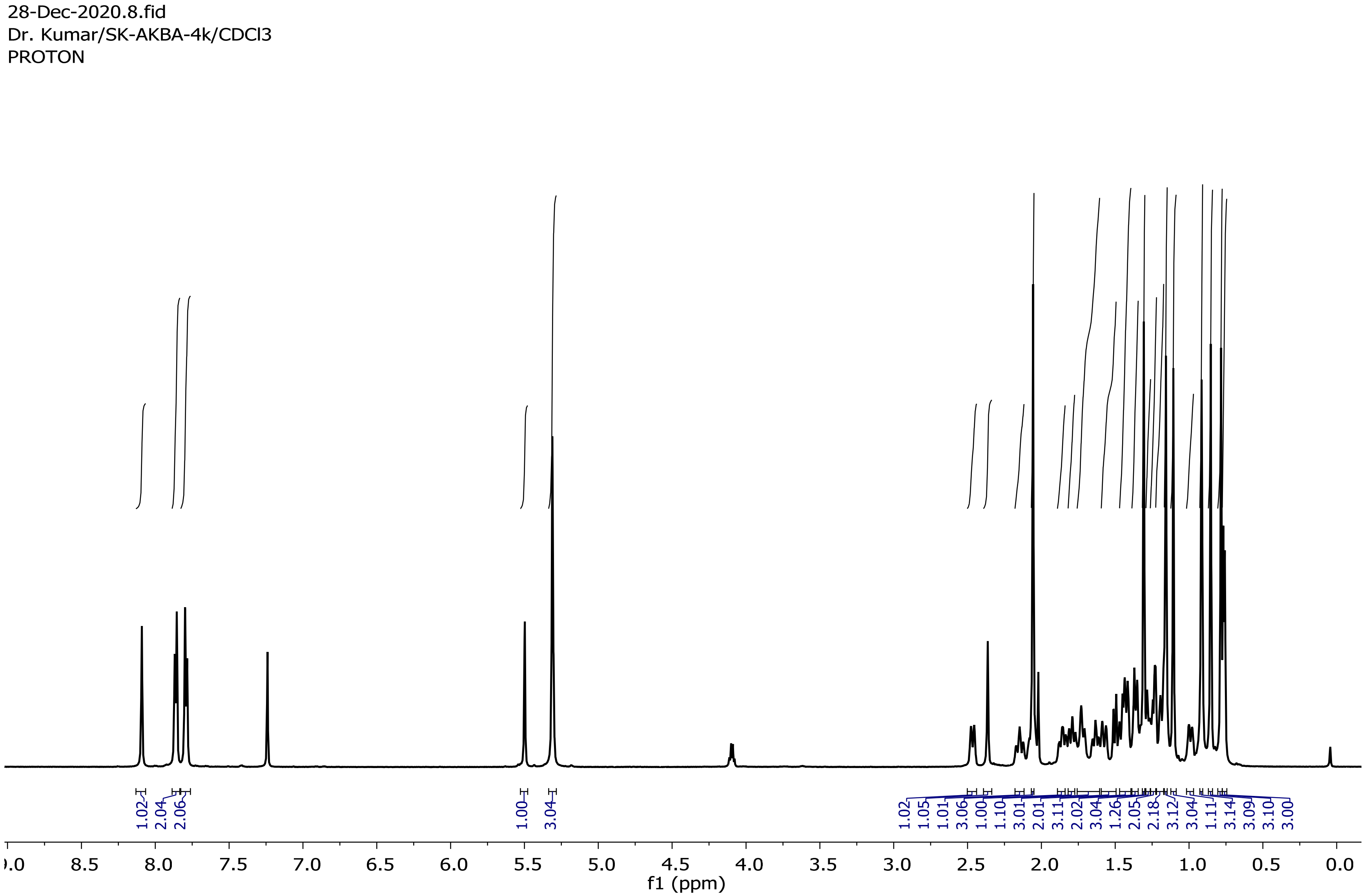

Figure S40: ^{1}H-NMR spectrum (600 MHz, $CDCl_3$) of compound **6k**

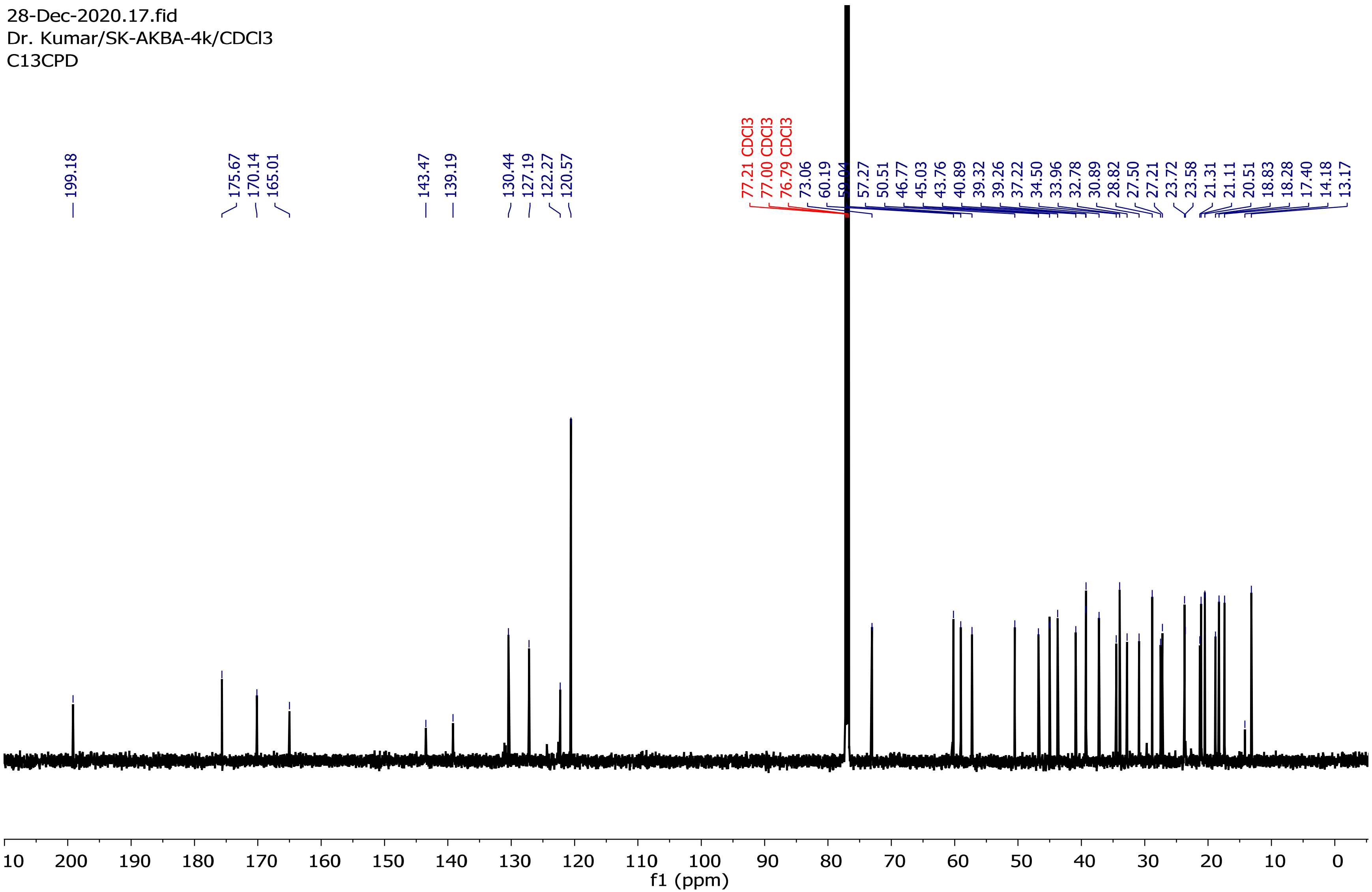

Figure S41: ^{13}C-NMR spectrum (150 MHz, $CDCl_3$) of compound **6k**

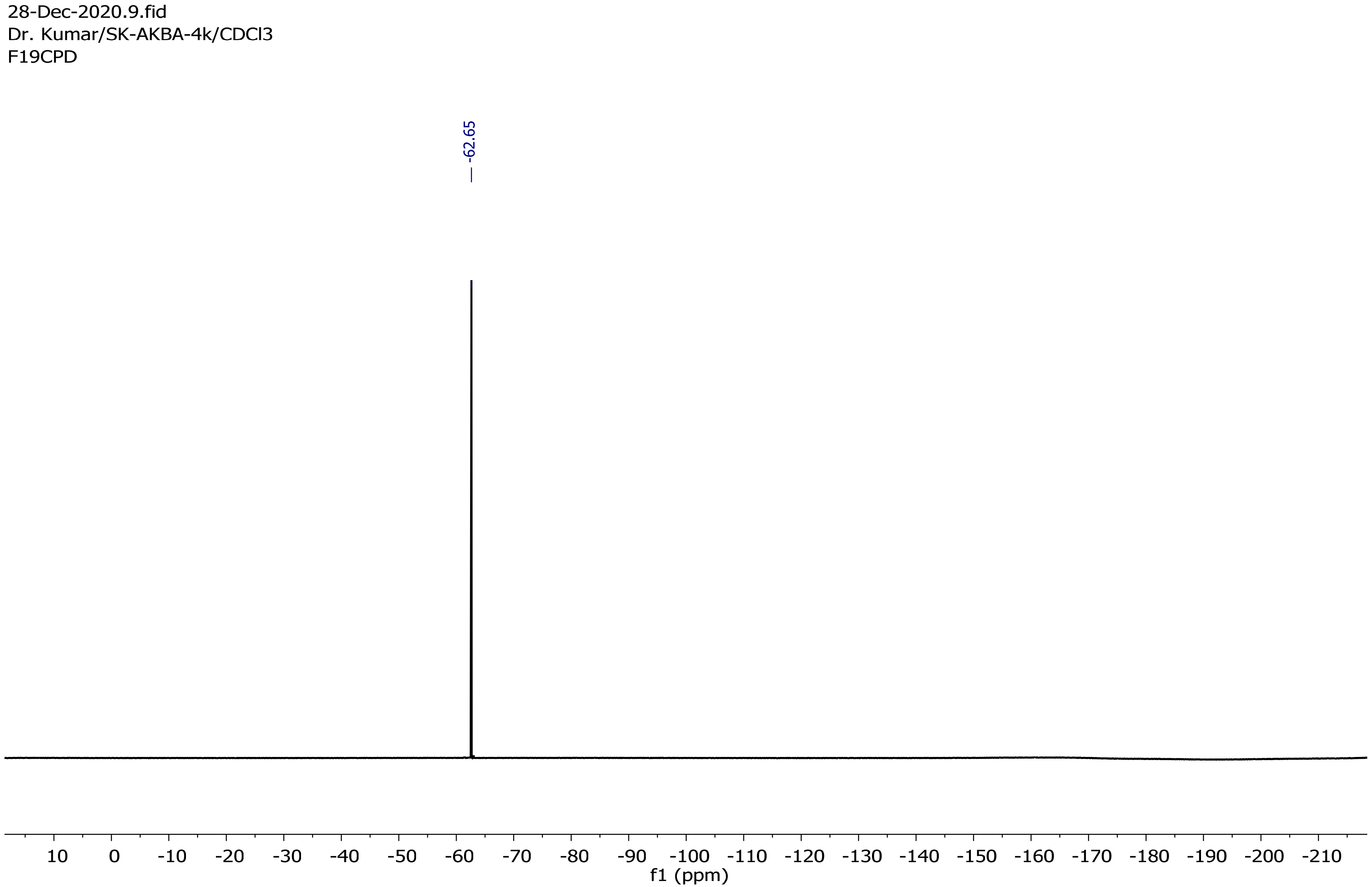

Figure S42: ^{19}F-NMR spectrum (564 MHz, $CDCl_3$) of compound **6k**

**Sample Name**	SK-AKBA-4K	**Position**	Vial 3	**Instrument Name**	Instrument 1
**User Name**		**Inj Vol**	2	**InjPosition**	
**Sample Type**	Sample	**IRM Calibration Status**	Success	**Data Filename**	SK-AKBA-4K_POS_01.d
**ACQ Method**	POSITIVE ION METHOD MS.m	**Comment**	SK	**Acquired Time**	28-Dec-20 2:43:36 PM

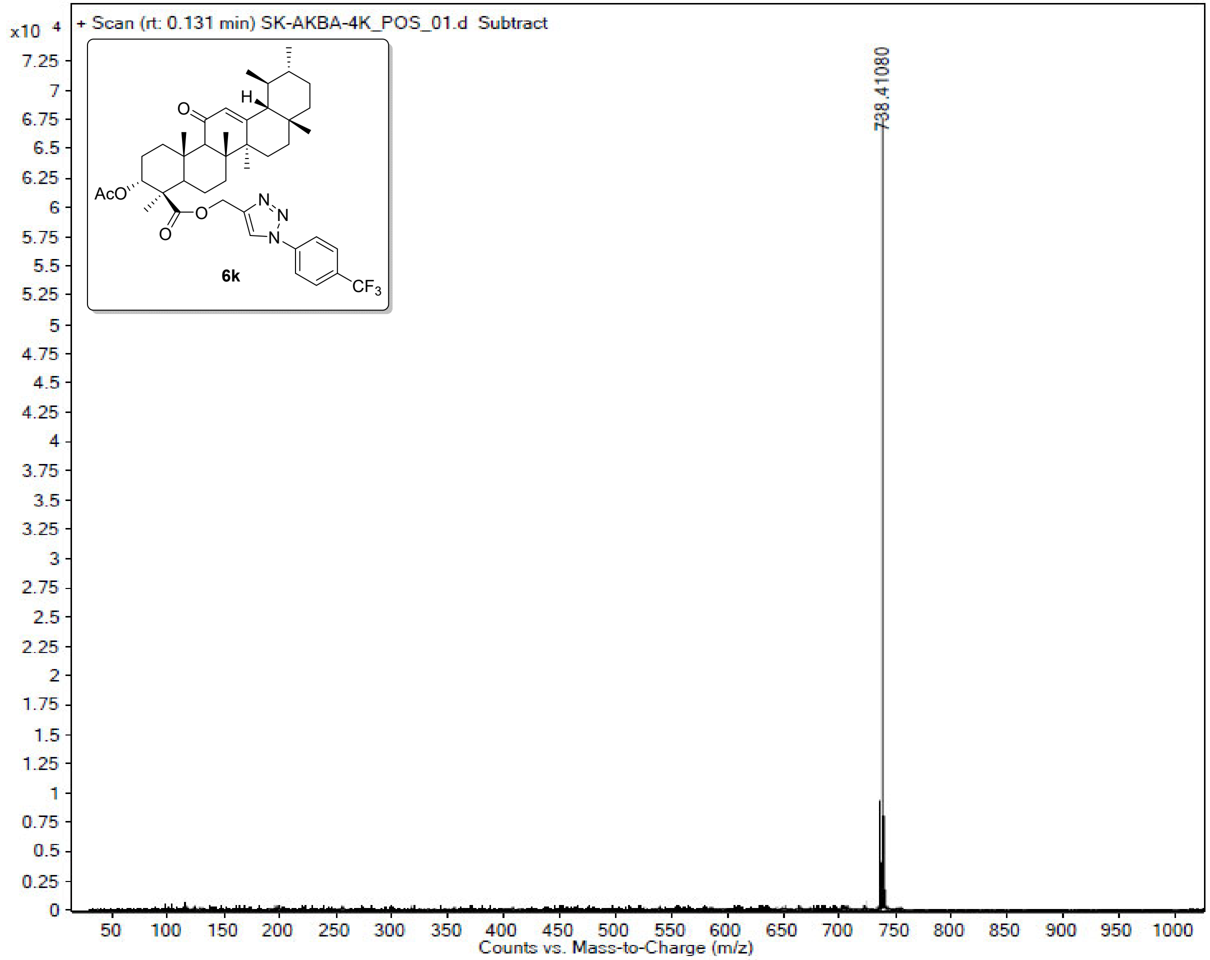

Figure S43: HRMS spectrum of compound **6k**

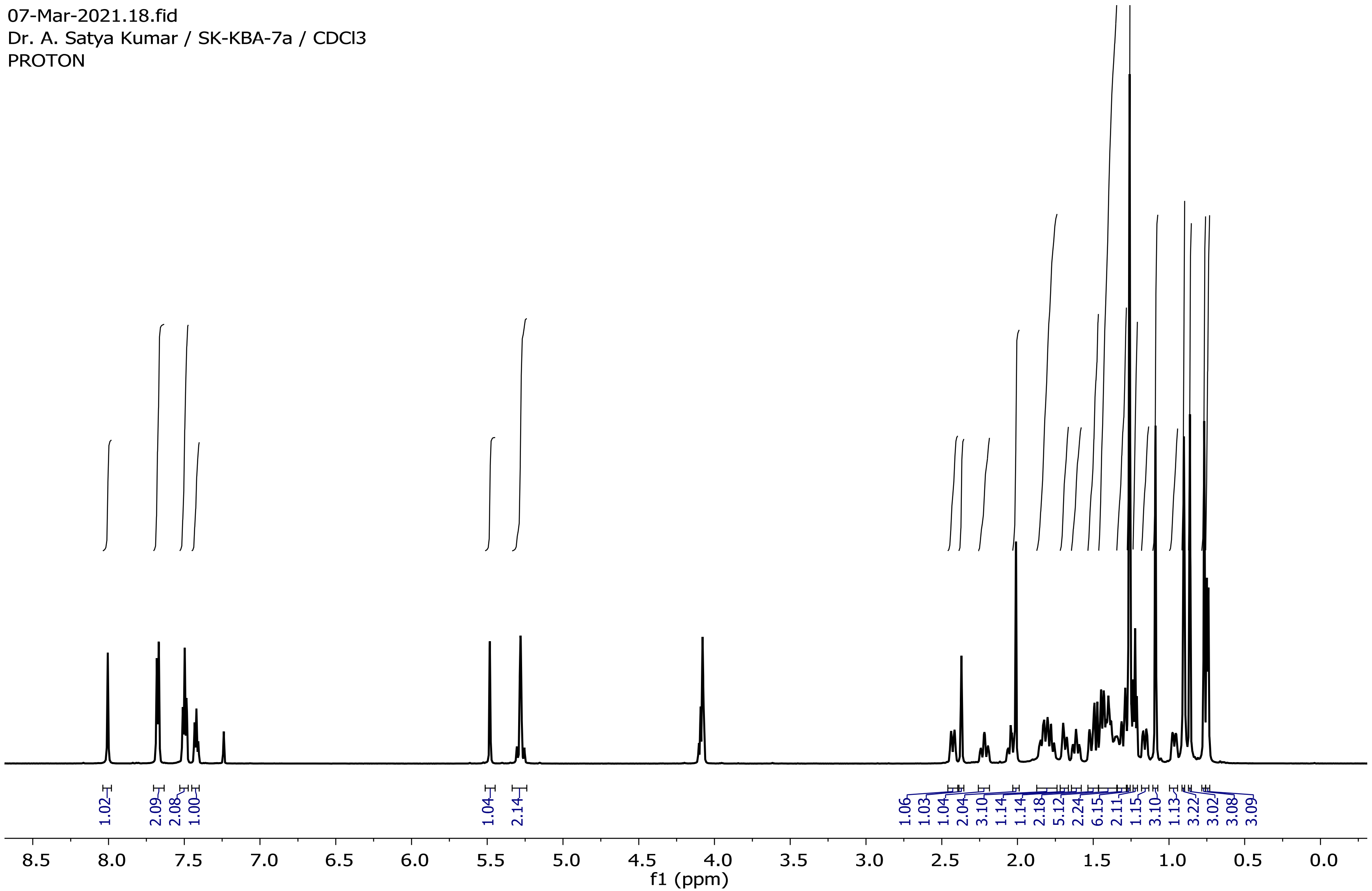

Figure S44: ^{1}H-NMR spectrum (600 MHz, $CDCl_3$) of compound **7a**

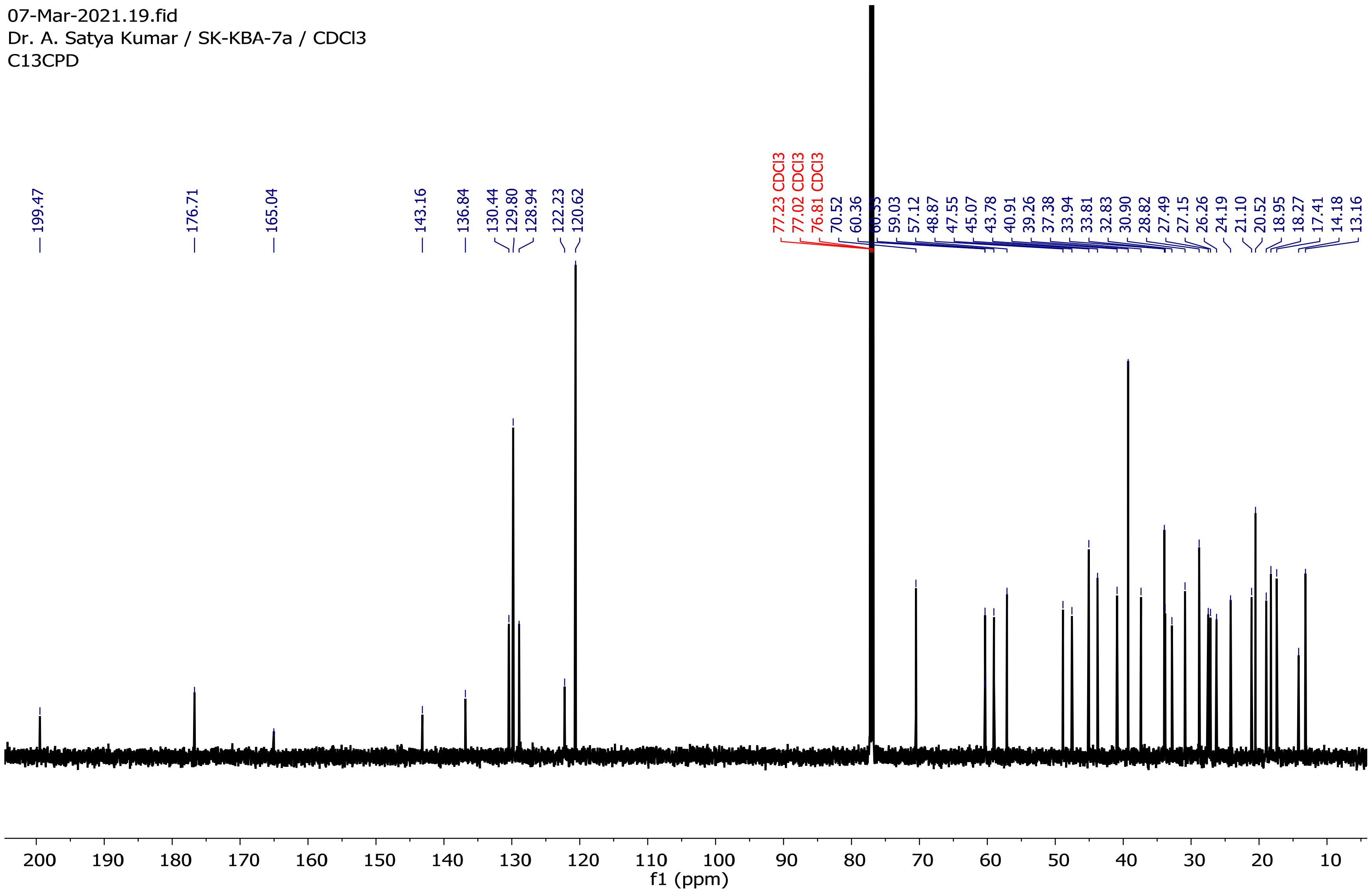

Figure S45: ^{13}C-NMR spectrum (150 MHz, $CDCl_3$) of compound **7a**

**Sample Name**	SK-KBA-7A	**Position**	Vial 41	**Instrument Name**	Instrument 1
**User Name**		**Inj Vol**	2	**InjPosition**	
**Sample Type**	Sample	**IRM Calibration Status**	Success	**Data Filename**	SK-KBA-7A.d
**ACQ Method**	POSITIVE ION METHOD MS.m	**Comment**	PARTHA	**Acquired Time**	17-Mar-21 12:09:31 PM

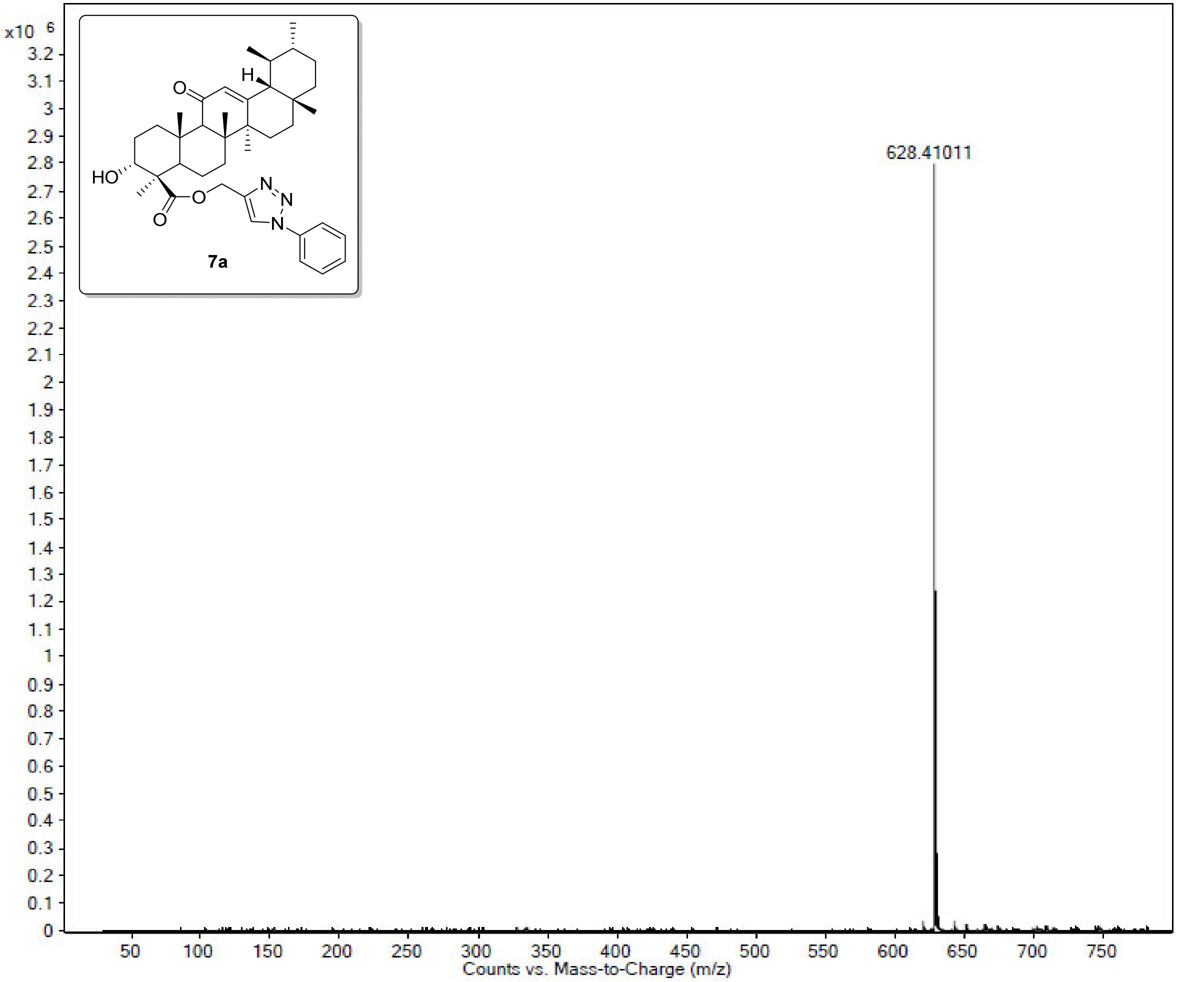

Figure S46: HRMS spectrum of compound **7a**

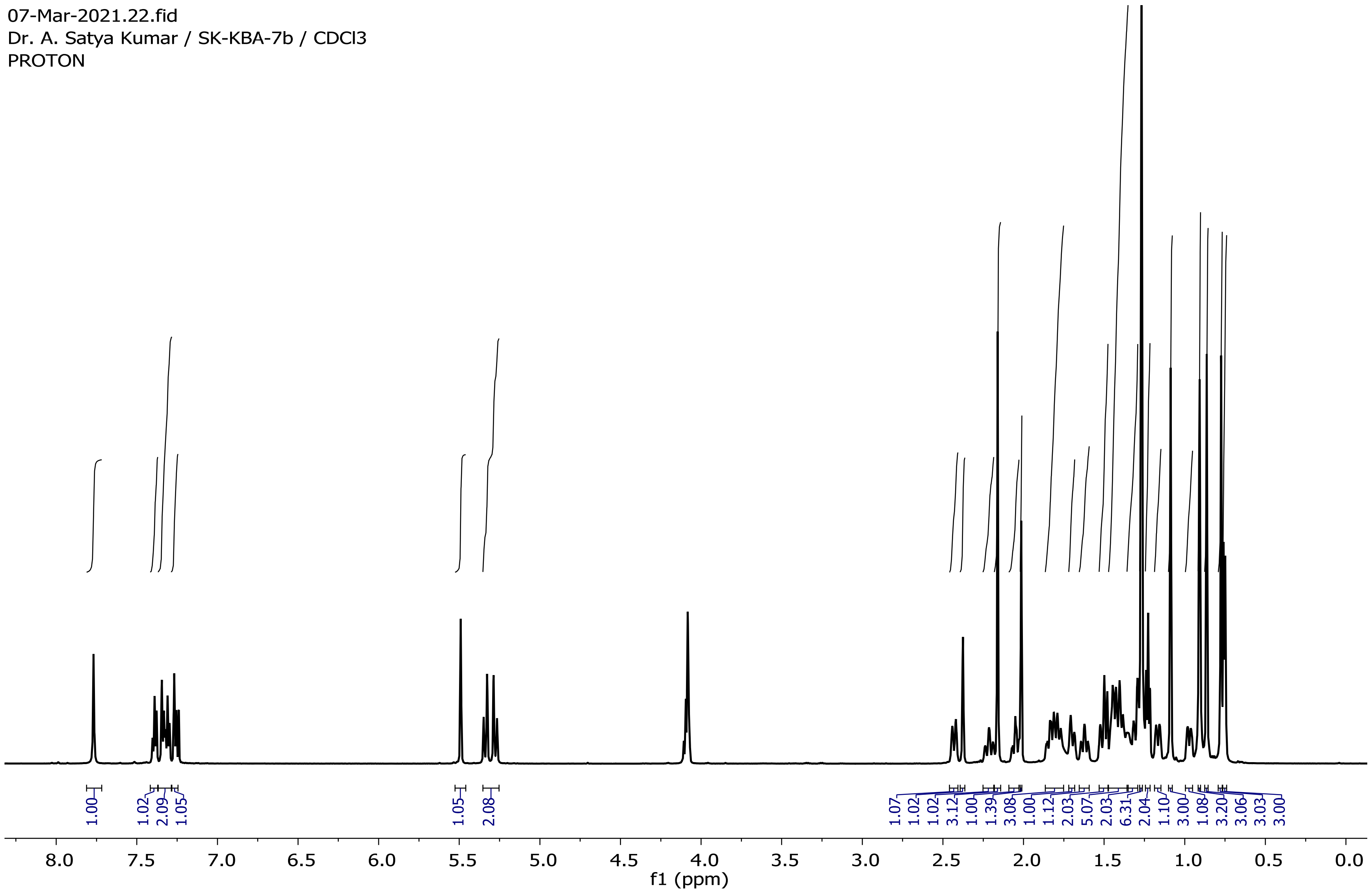

Figure S47: ^{1}H-NMR spectrum (600 MHz, $CDCl_3$) of compound **7b**

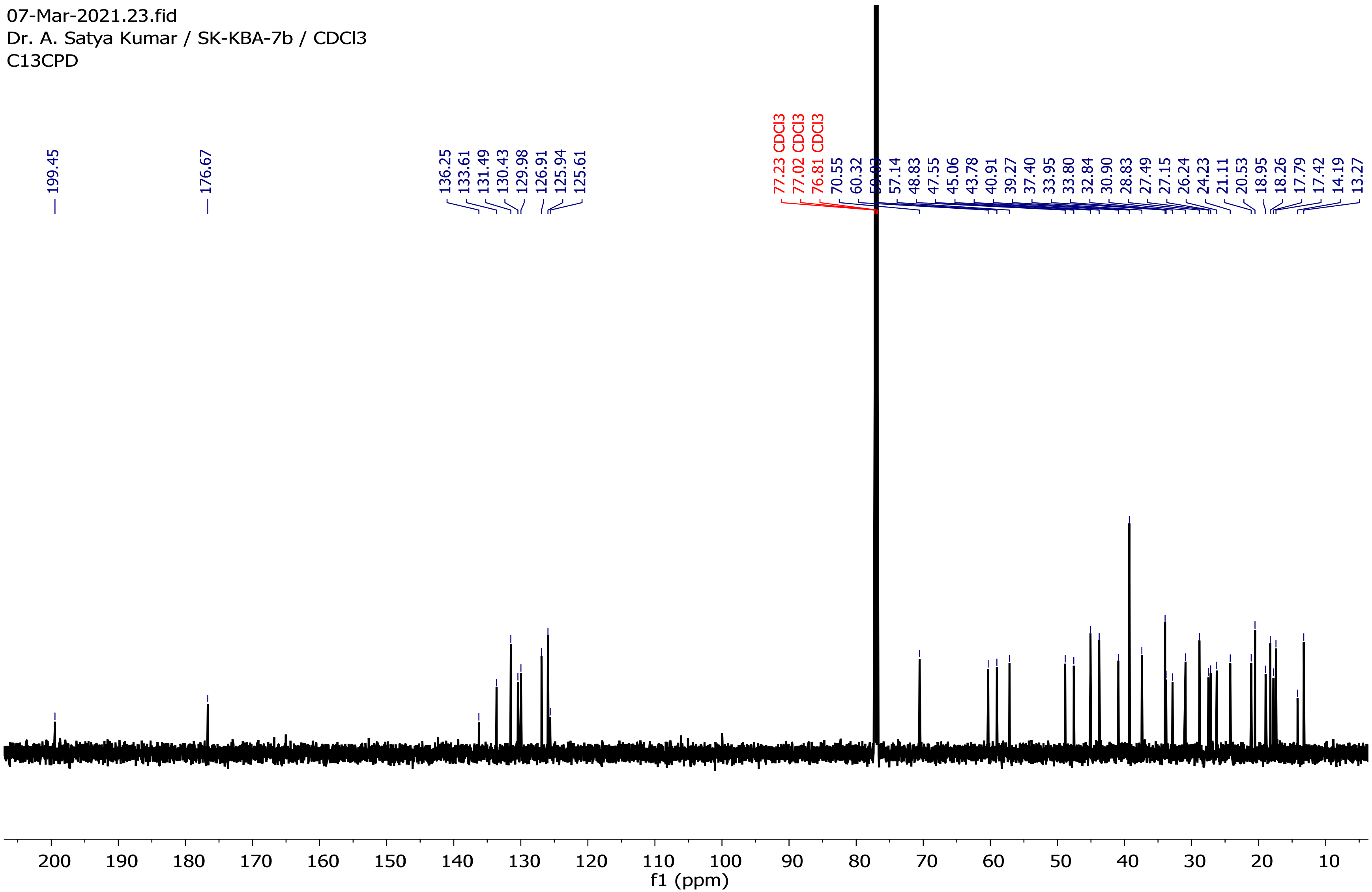

Figure S48: ^{13}C-NMR spectrum (150 MHz, $CDCl_3$) of compound **7b**

**Sample Name**	SK-KBA-7B	**Position**	Vial 42	**Instrument Name**	Instrument 1
**User Name**		**Inj Vol**	2	**InjPosition**	
**Sample Type**	Sample	**IRM Calibration Status**	Success	**Data Filename**	SK-KBA-7B.d
**ACQ Method**	POSITIVE ION METHOD MS.m	**Comment**	PARTHA	**Acquired Time**	17-Mar-21 12:18:56 PM

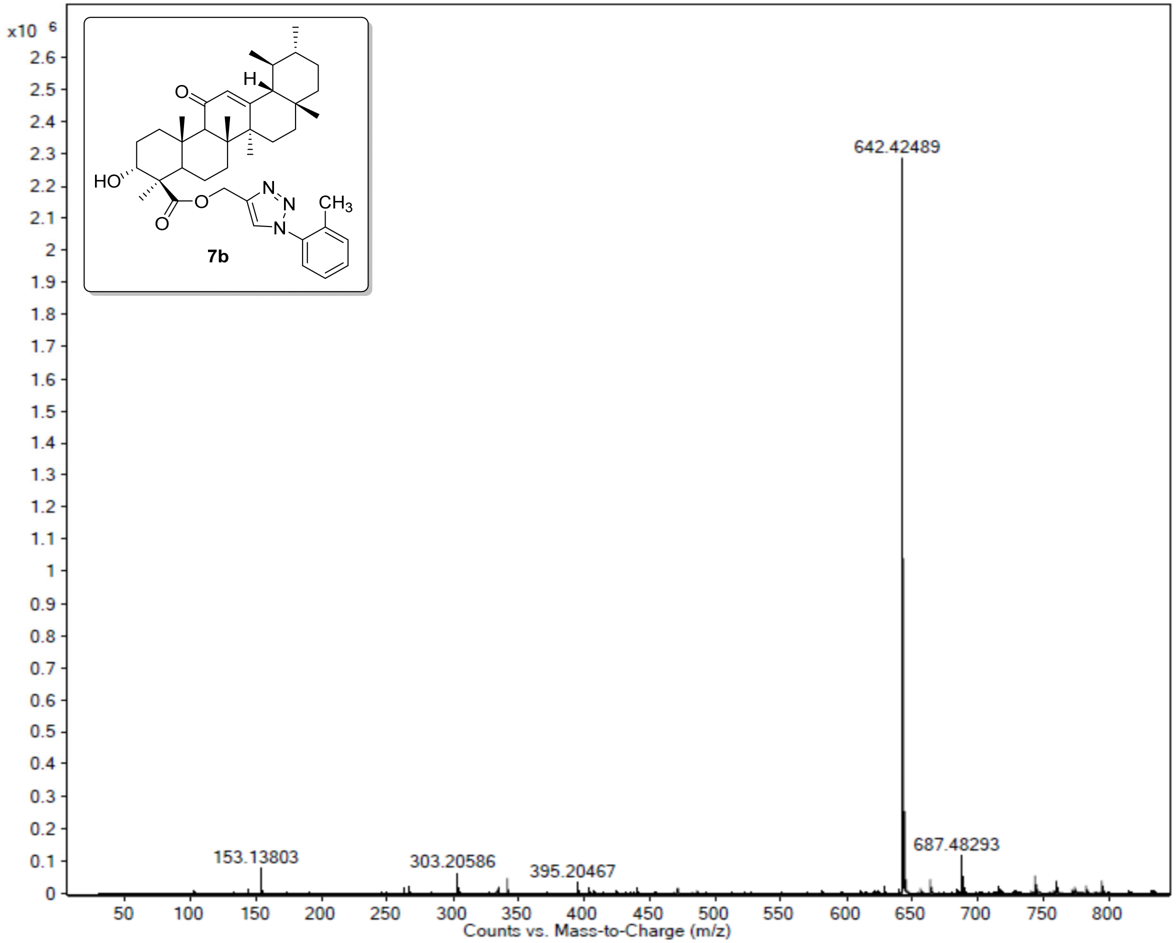

Figure S49: HRMS spectrum of compound **7b**

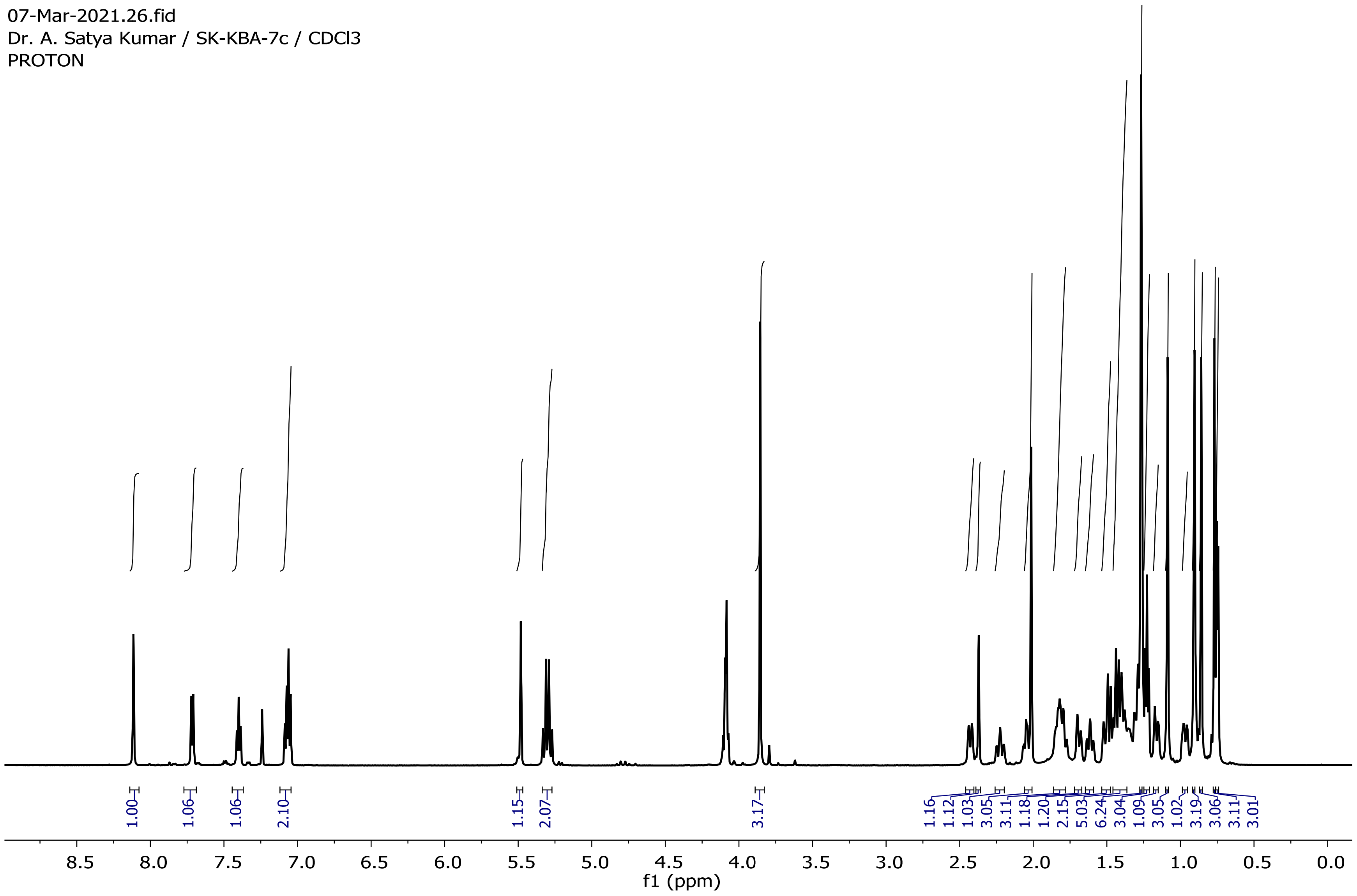

Figure S50: ^{1}H-NMR spectrum (600 MHz, $CDCl_3$) of compound **7c**

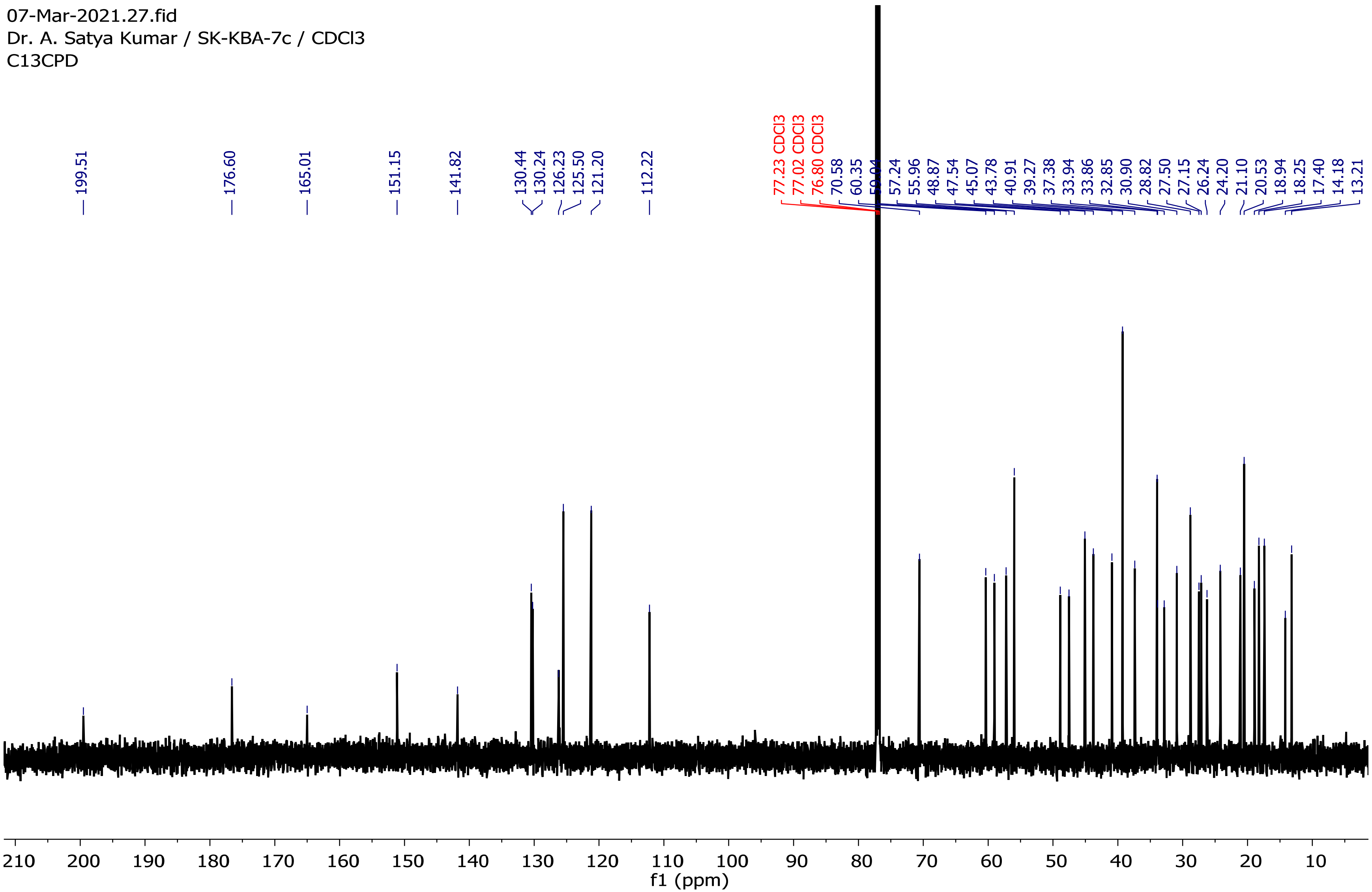

Figure S51: ^{13}C-NMR spectrum (150 MHz, $CDCl_3$) of compound **7c**

**Sample Name**	SK-KBA-7C	**Position**	Vial 43	**Instrument Name**	Instrument 1
**User Name**		**Inj Vol**	2	**InjPosition**	
**Sample Type**	Sample	**IRM Calibration Status**	Success	**Data Filename**	SK-KBA-7C.d
**ACQ Method**	POSITIVE ION METHOD MS.m	**Comment**	PARTHA	**Acquired Time**	17-Mar-21 12:24:32 PM

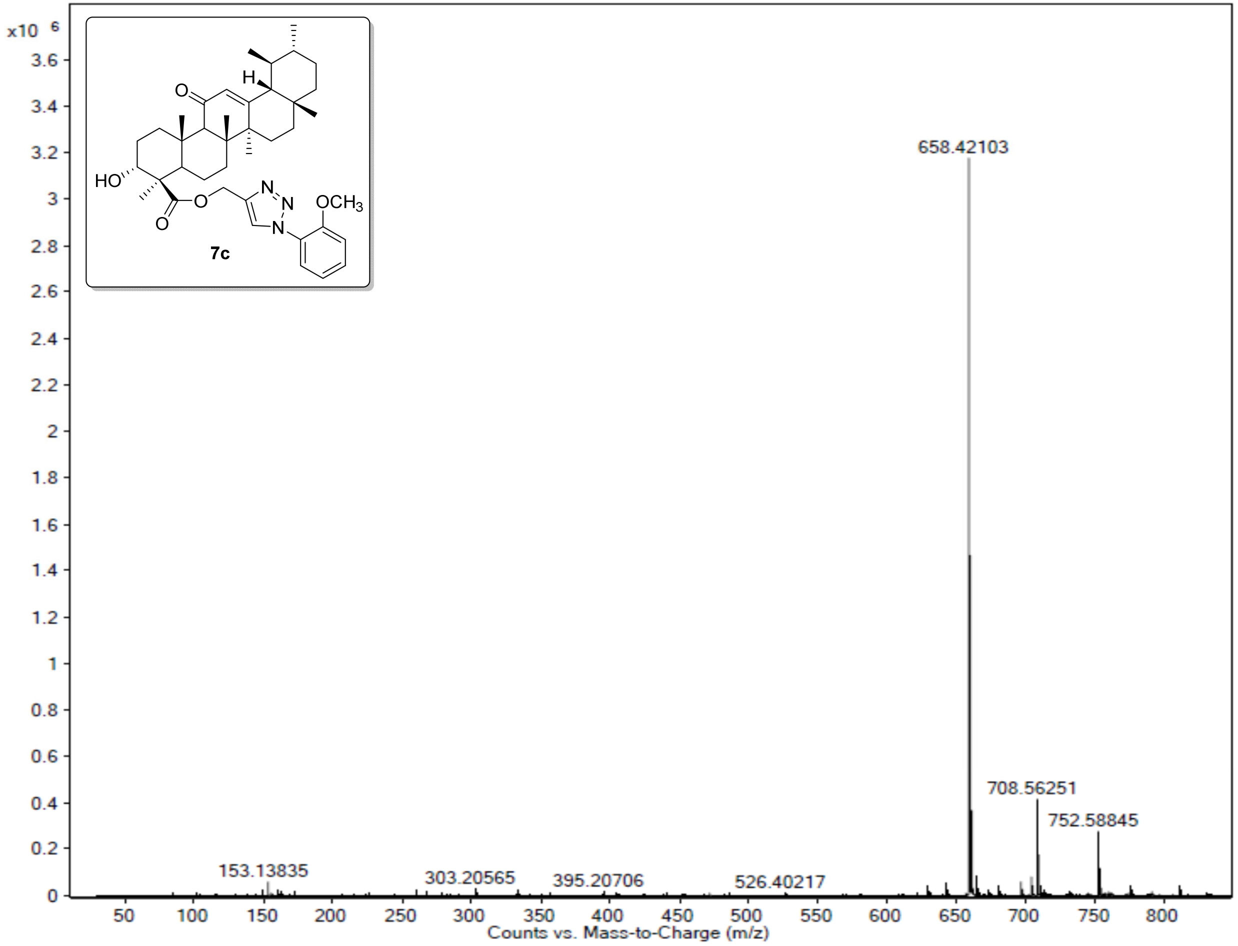

Figure S52: HRMS spectrum of compound **7c**

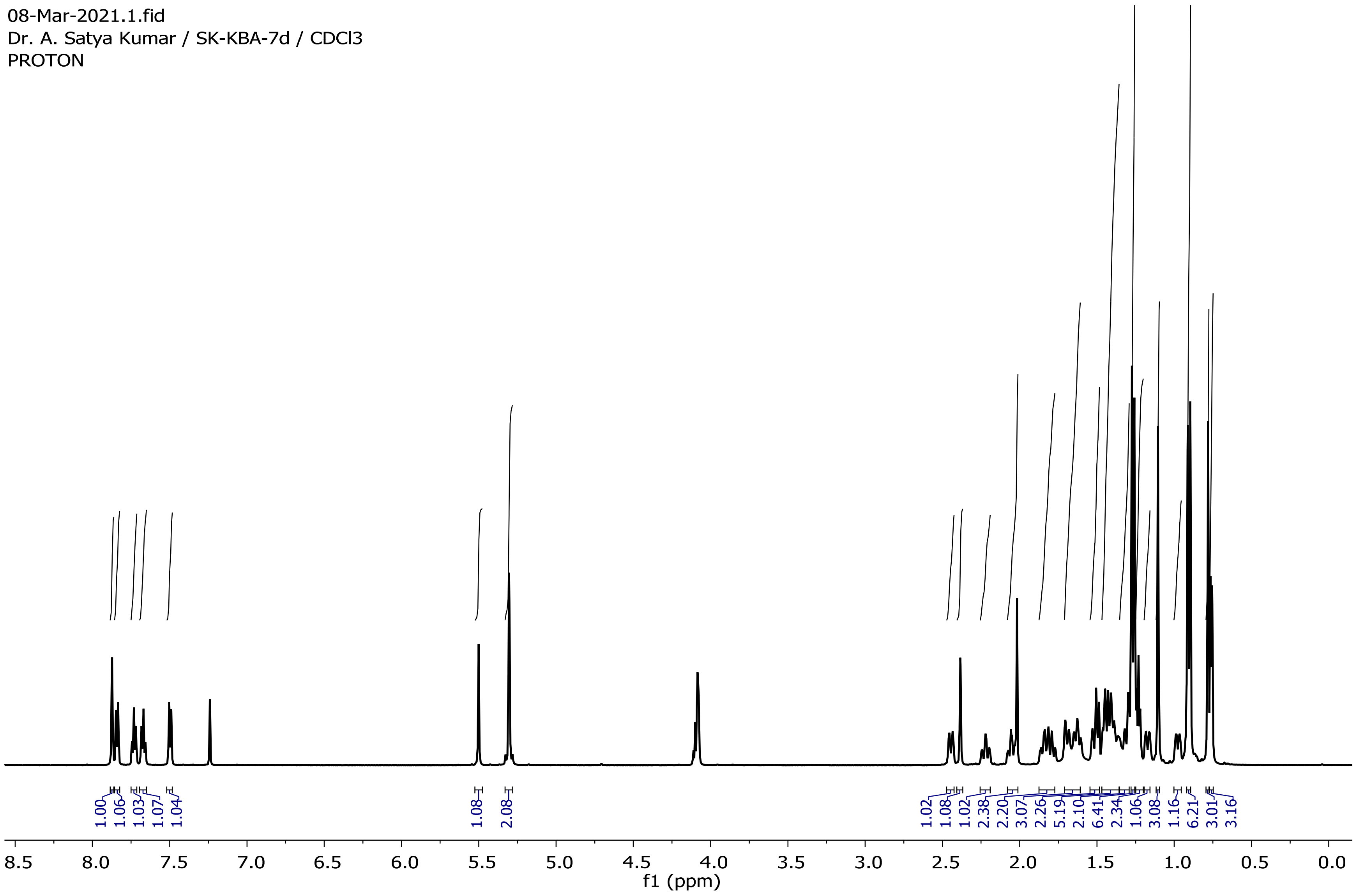

Figure S53: ^{1}H-NMR spectrum (600 MHz, $CDCl_3$) of compound **7d**

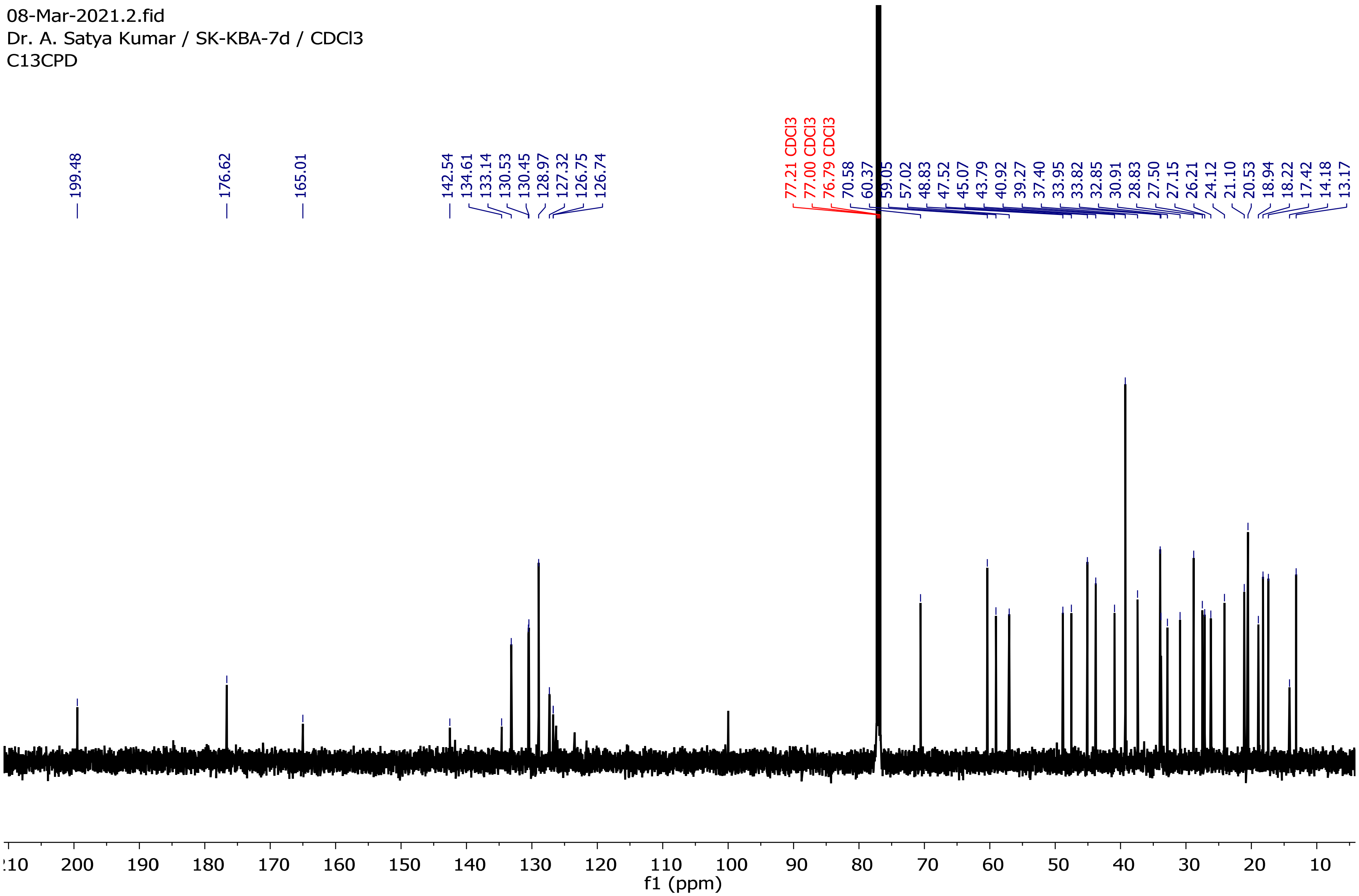

Figure S54: ^{13}C-NMR spectrum (150 MHz, $CDCl_3$) of compound **7d**

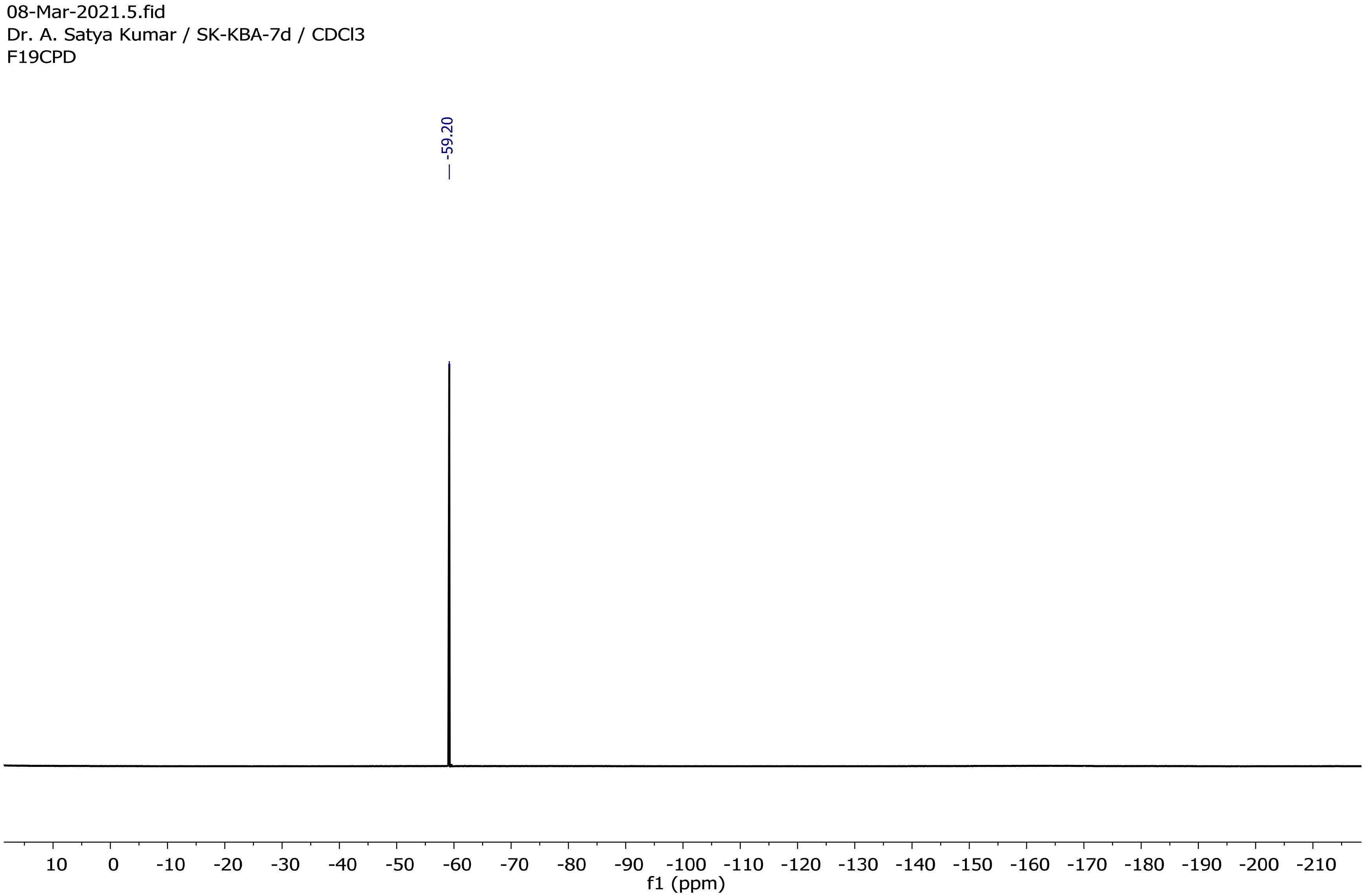

Figure S55: ${}^{19}F$-NMR spectrum (564 MHz, $CDCl_3$) of compound **7d**

**Sample Name**	SK-KBA-7D	**Position**	Vial 44	**Instrument Name**	Instrument 1
**User Name**		**Inj Vol**	2	**InjPosition**	
**Sample Type**	Sample	**IRM Calibration Status**	Success	**Data Filename**	SK-KBA-7D.d
**ACQ Method**	POSITIVE ION METHOD MS.m	**Comment**	PARTHA	**Acquired Time**	17-Mar-21 12:30:19 PM

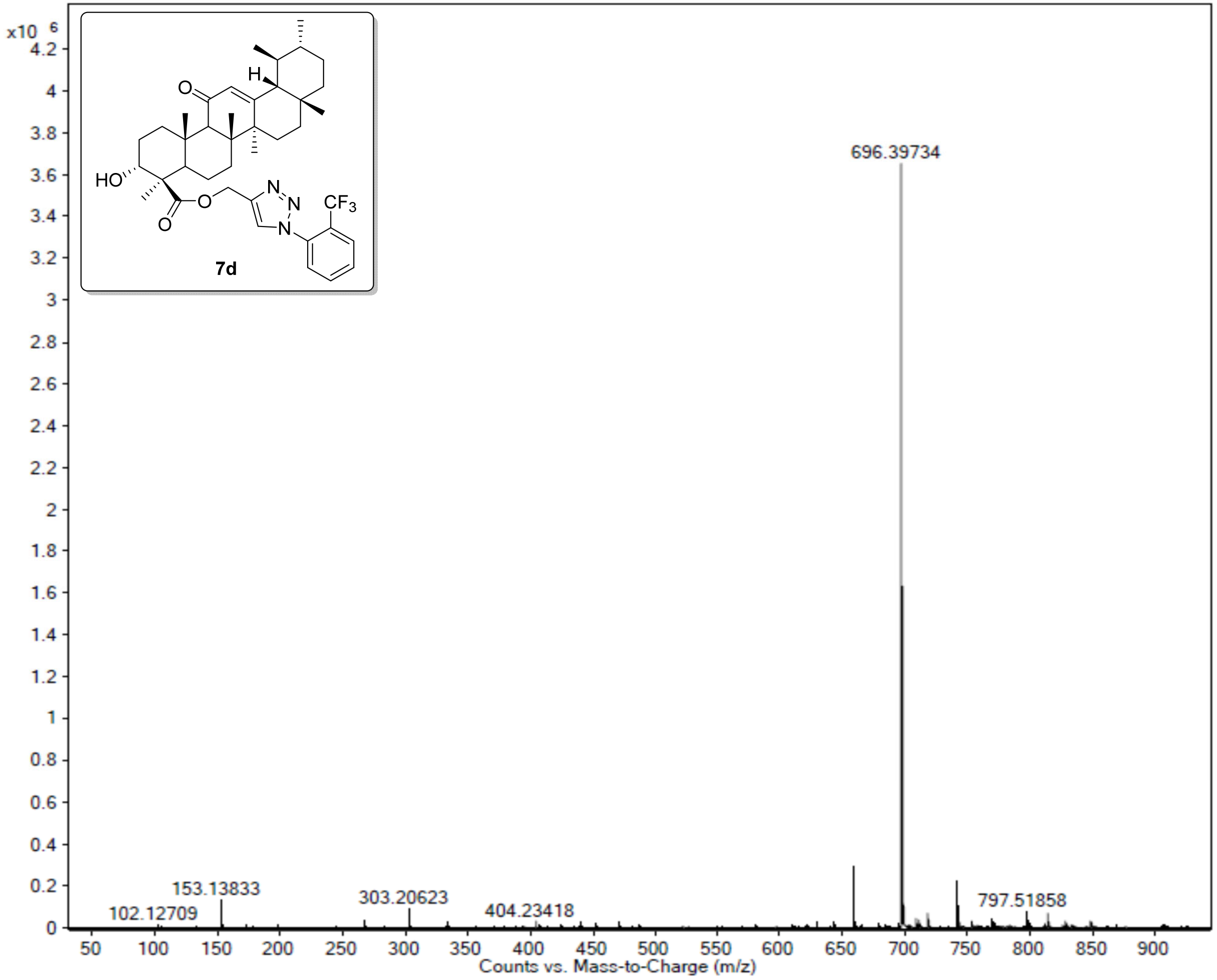

Figure S56: HRMS spectrum of compound **7d**

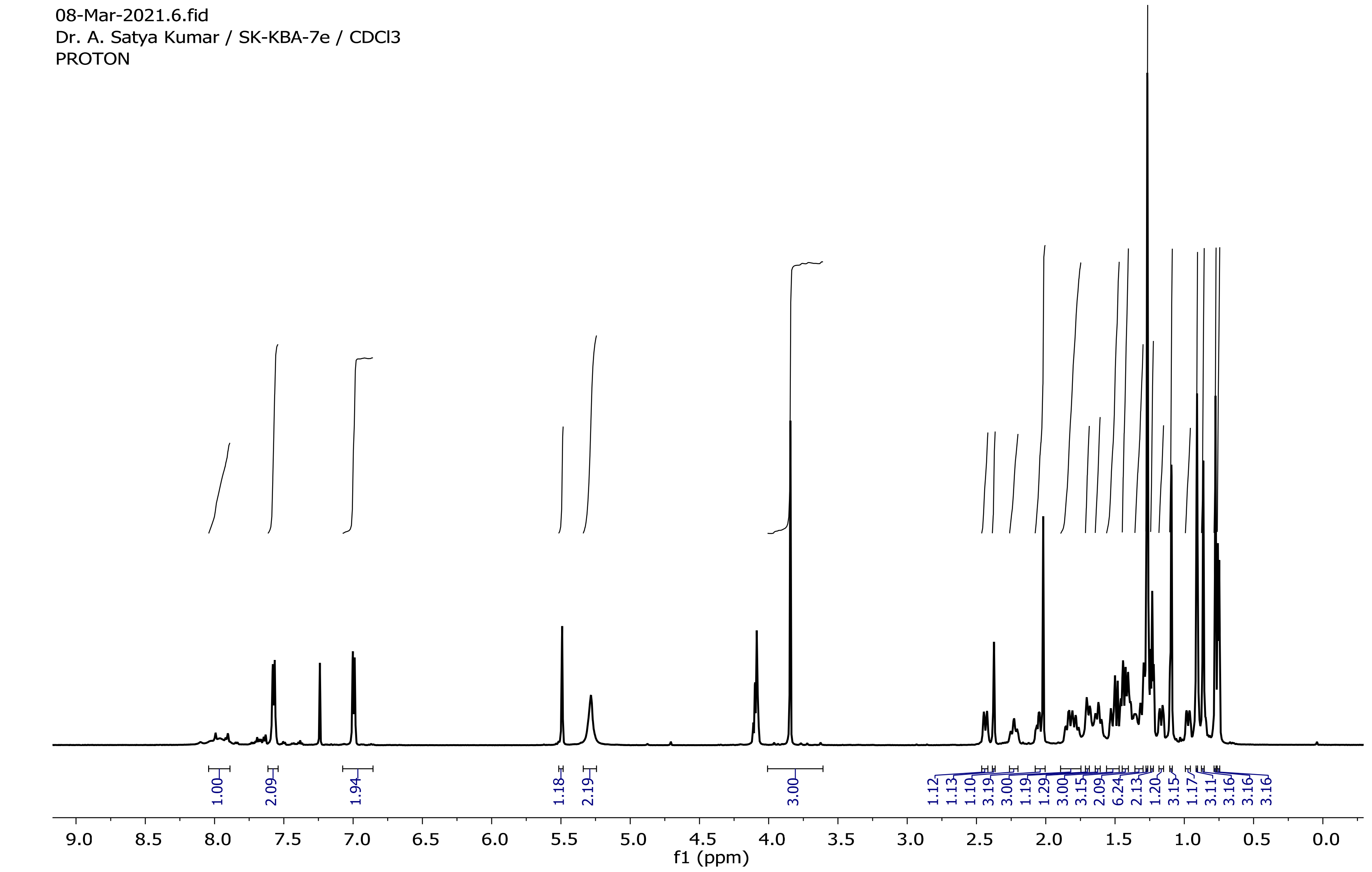

Figure S57: ^{1}H-NMR spectrum (600 MHz, $CDCl_3$) of compound **7e**

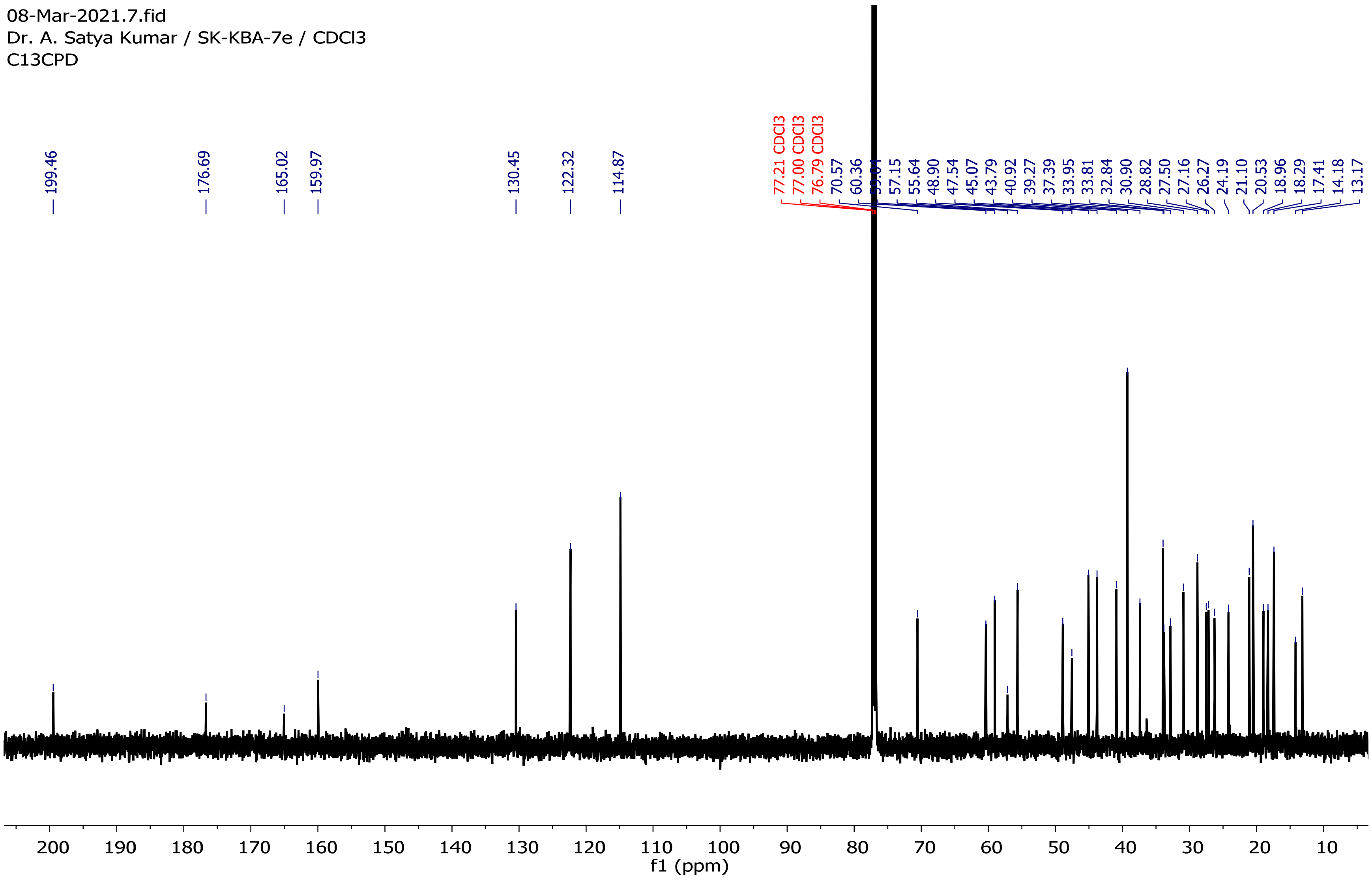

Figure S58: ^{13}C-NMR spectrum (150 MHz, $CDCl_3$) of compound **7e**

**Sample Name**	SK-KBA-7E	**Position**	Vial 45	**Instrument Name**	Instrument 1
**User Name**		**Inj Vol**	2	**InjPosition**	
**Sample Type**	Sample	**IRM Calibration Status**	Success	**Data Filename**	SK-KBA-7E.d
**ACQ Method**	POSITIVE ION METHOD MS.m	**Comment**	PARTHA	**Acquired Time**	17-Mar-21 12:36:03 PM

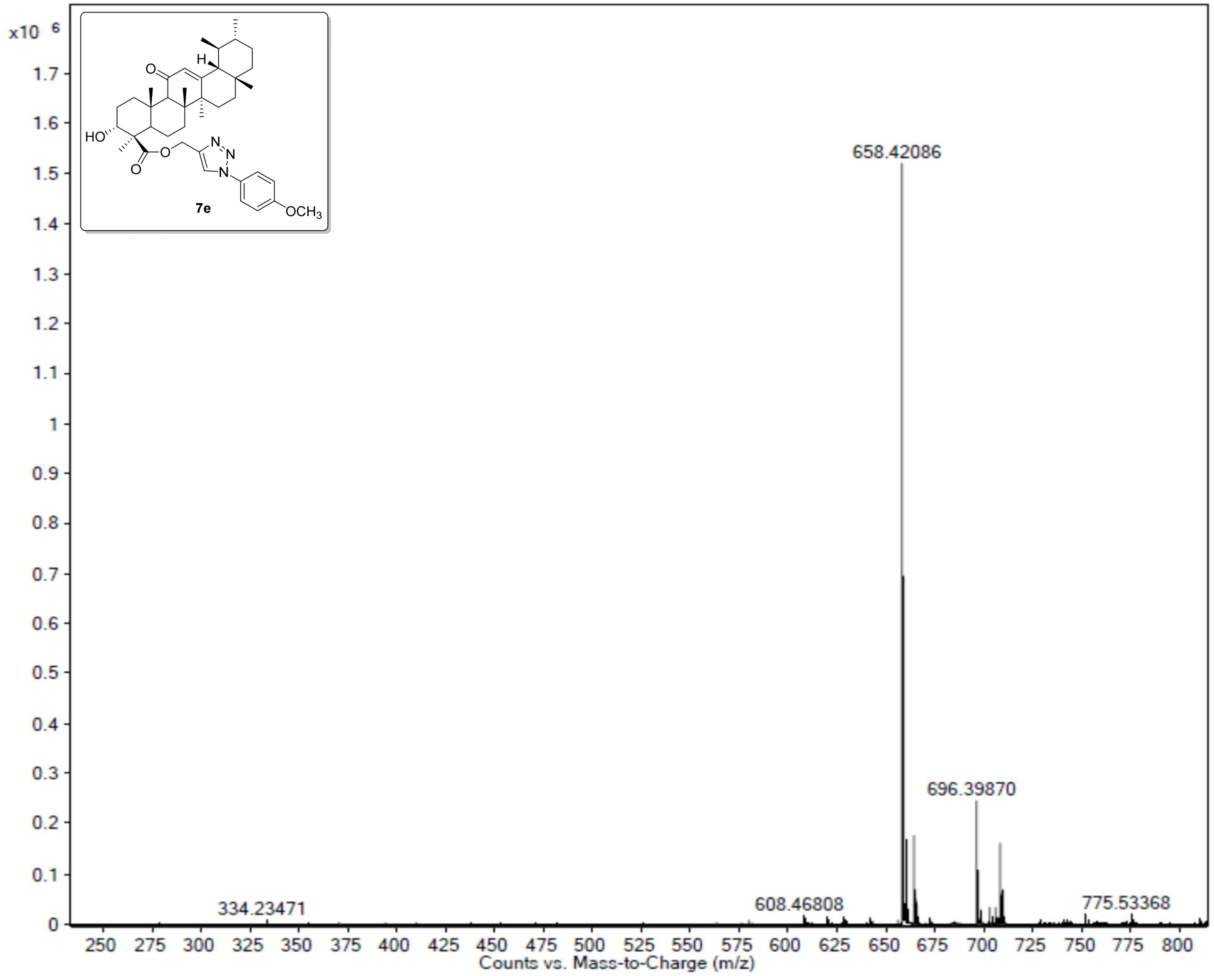

Figure S59: HRMS spectrum of compound **7e**

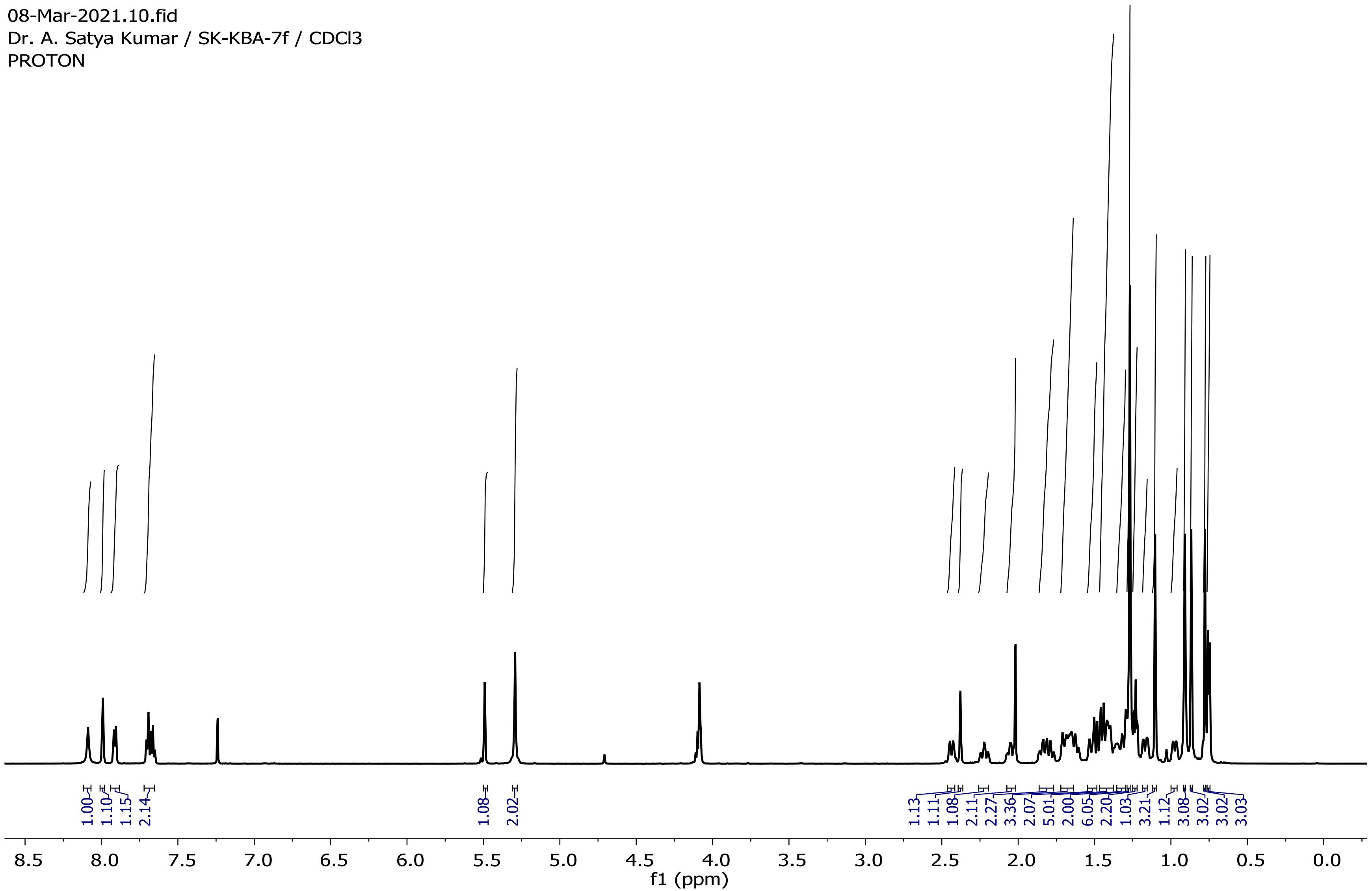

Figure S60: ^{1}H-NMR spectrum (600 MHz, $CDCl_3$) of compound **7f**

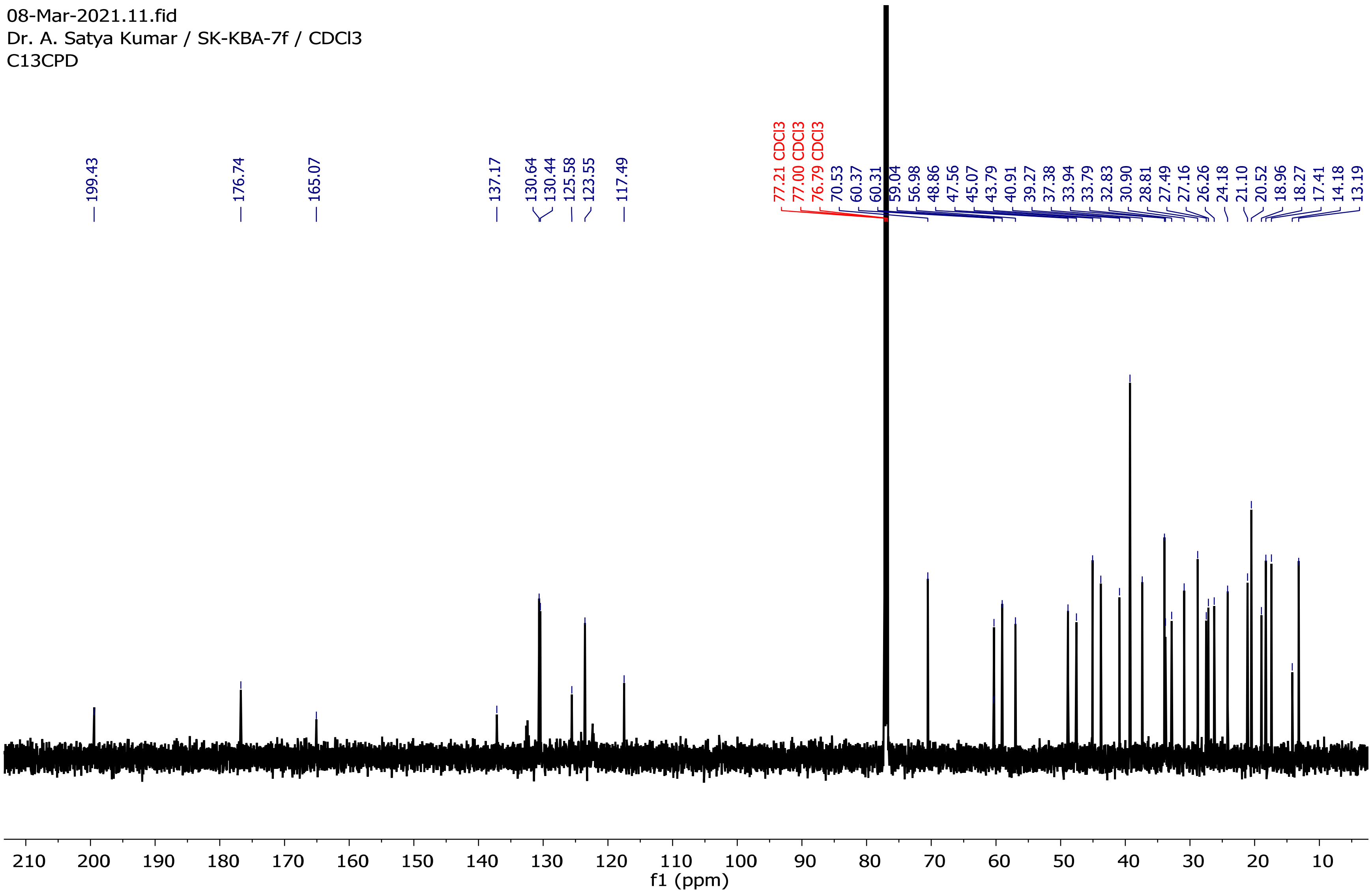

Figure S61: ^{13}C-NMR spectrum (150 MHz, $CDCl_3$) of compound **7f**

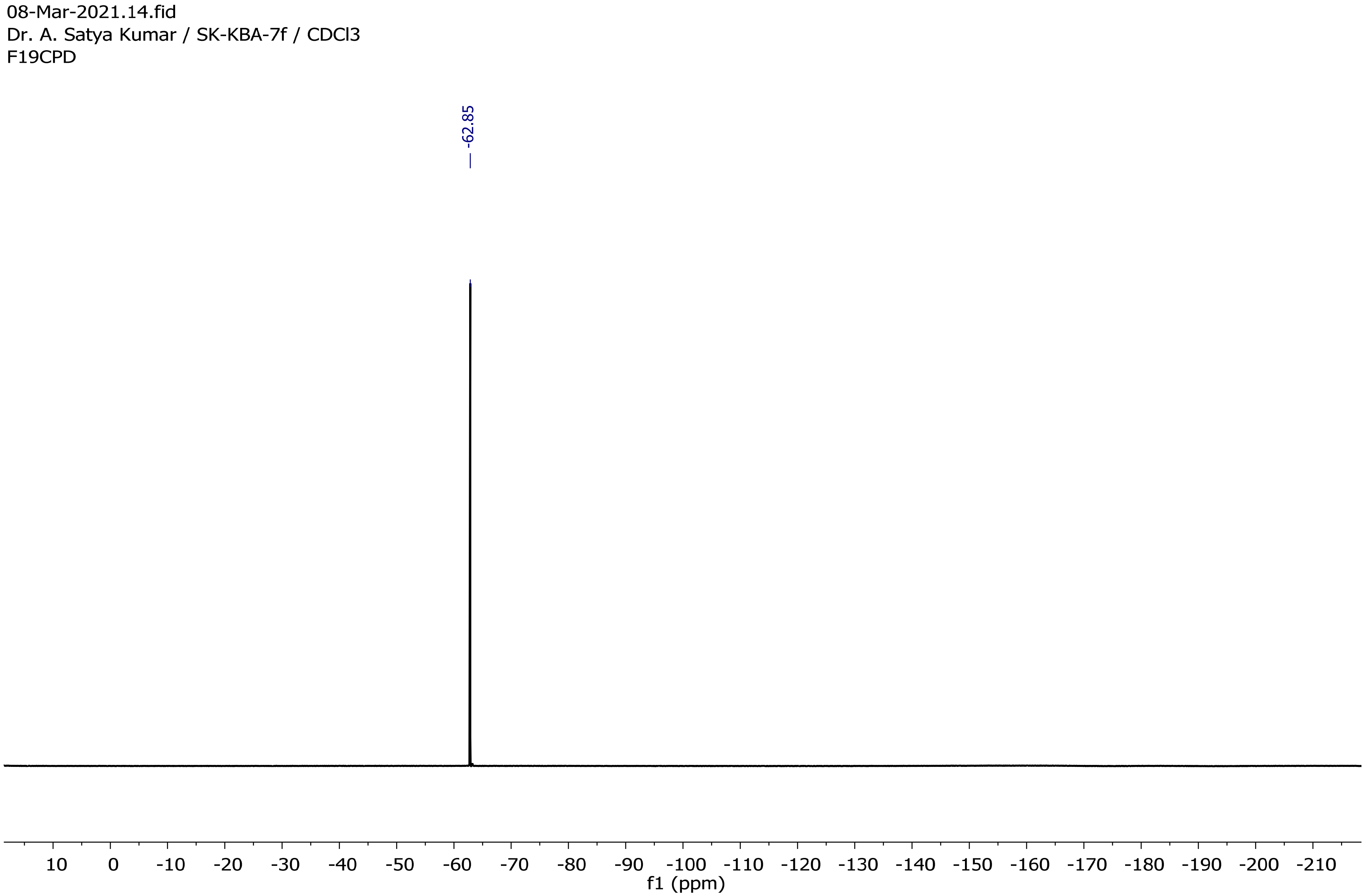

Figure S62: ^{19}F-NMR spectrum (564 MHz, $CDCl_3$) of compound **7f**

**Sample Name**	SK-KBA-7F	**Position**	Vial 46	**Instrument Name**	Instrument 1
**User Name**		**Inj Vol**	2	**InjPosition**	
**Sample Type**	Sample	**IRM Calibration Status**	Success	**Data Filename**	SK-KBA-7F.d
**ACQ Method**	POSITIVE ION METHOD MS.m	**Comment**	PARTHA	**Acquired Time**	17-Mar-21 12:41:31 PM

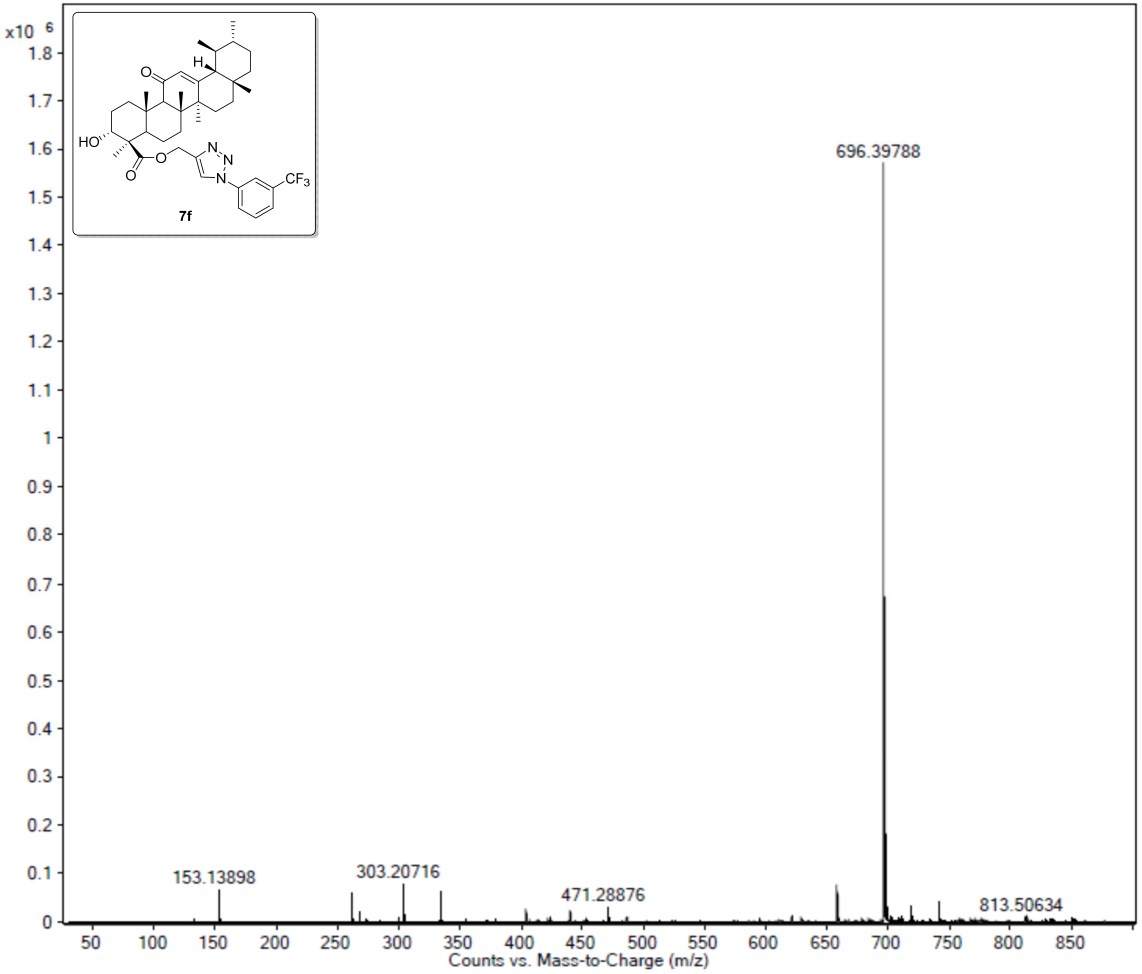

Figure S63: HRMS spectrum of compound **7f**

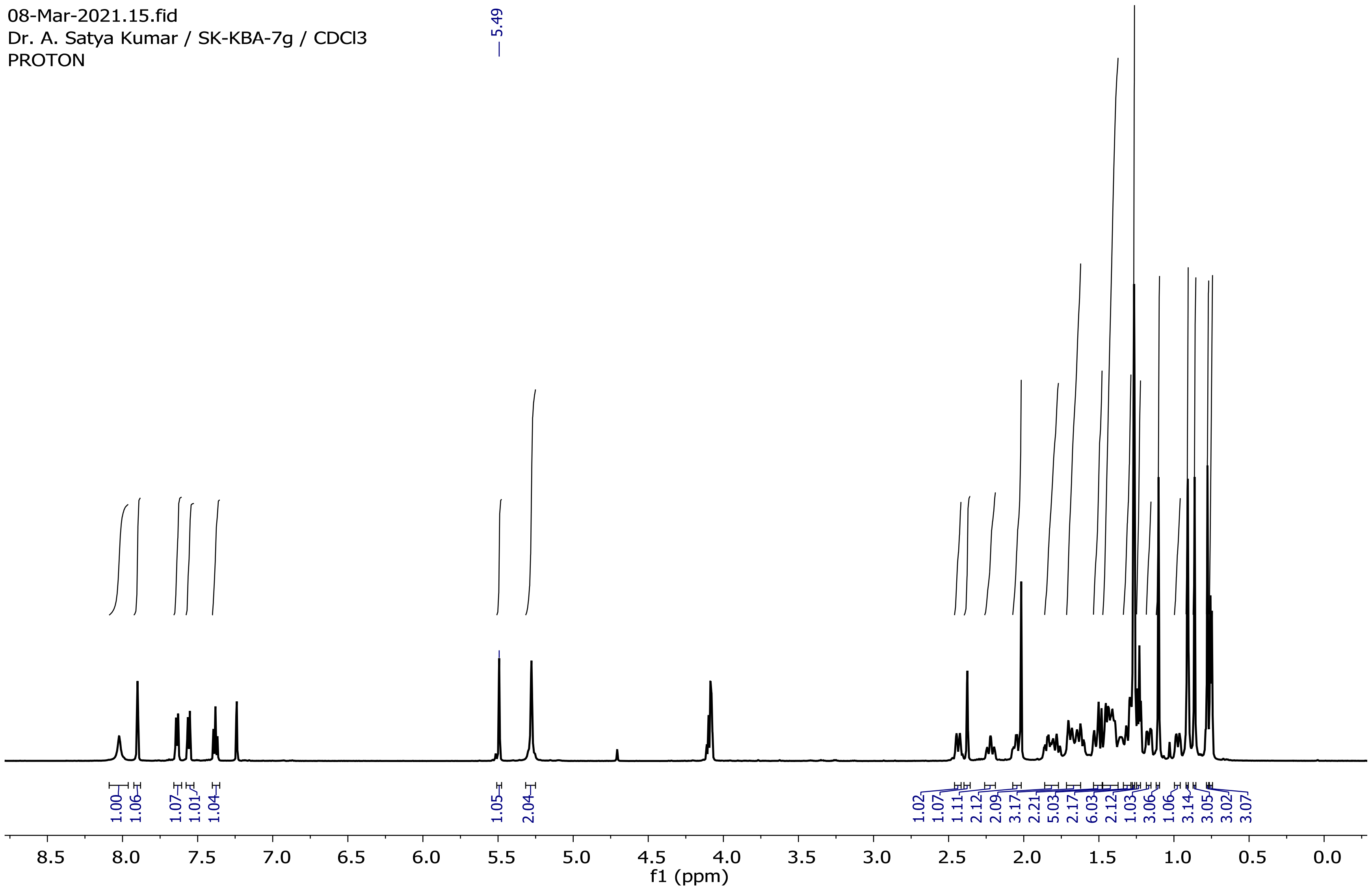

Figure S64: ^{1}H-NMR spectrum (600 MHz, $CDCl_3$) of compound **7g**

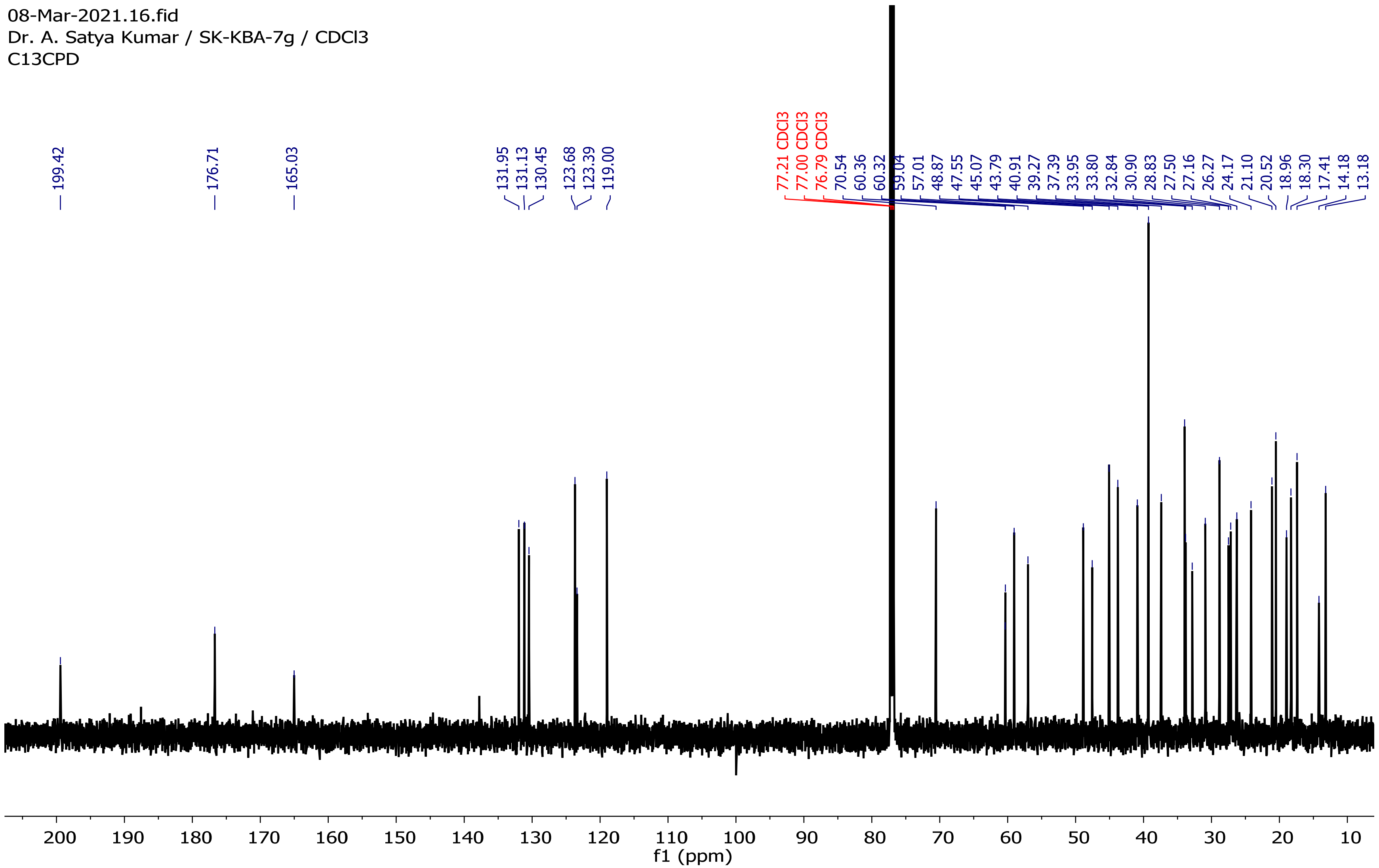

Figure S65: ^{13}C-NMR spectrum (150 MHz, $CDCl_3$) of compound **7g**

**Sample Name**	SK-KBA-7G	**Position**	Vial 47	**Instrument Name**	Instrument 1
**User Name**		**Inj Vol**	2	**InjPosition**	
**Sample Type**	Sample	**IRM Calibration Status**	Success	**Data Filename**	SK-KBA-7G.d
**ACQ Method**	POSITIVE ION METHOD MS.m	**Comment**	PARTHA	**Acquired Time**	17-Mar-21 12:47:05 PM

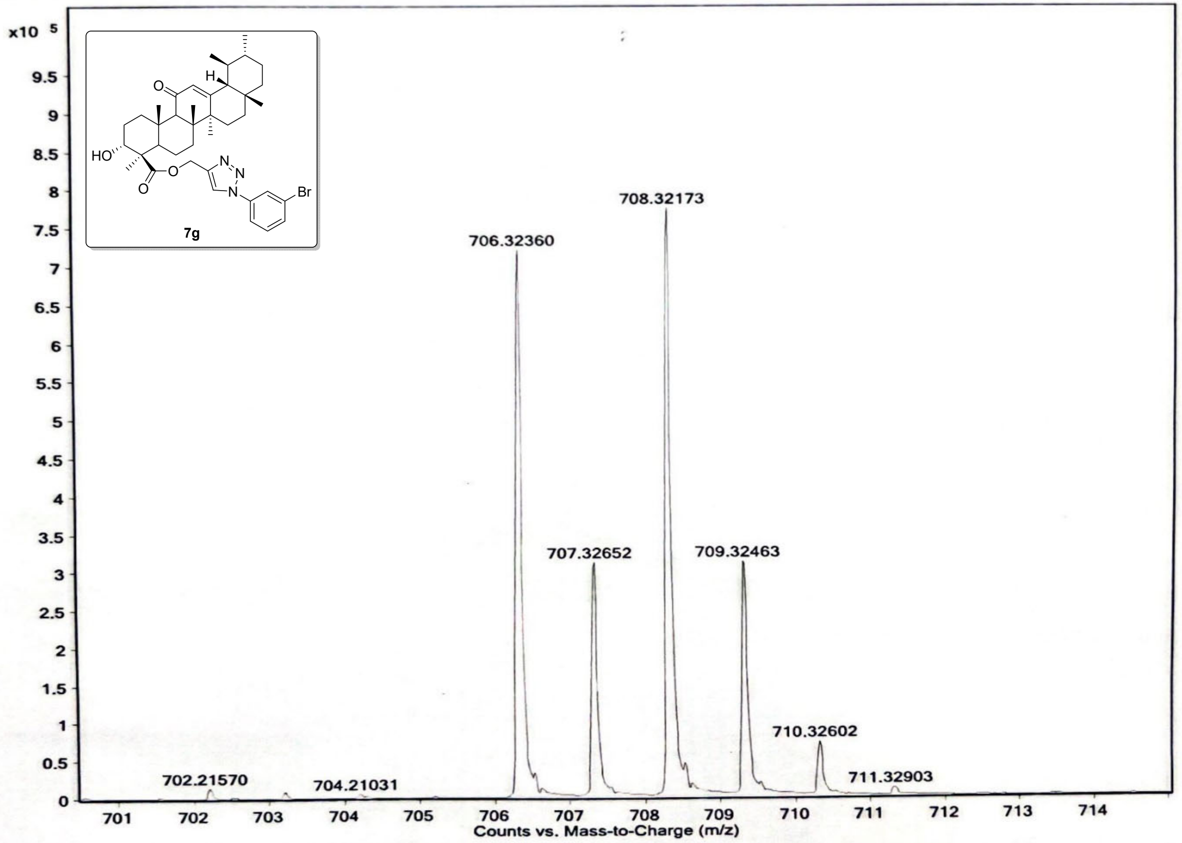

Figure S66: HRMS spectrum of compound **7g**

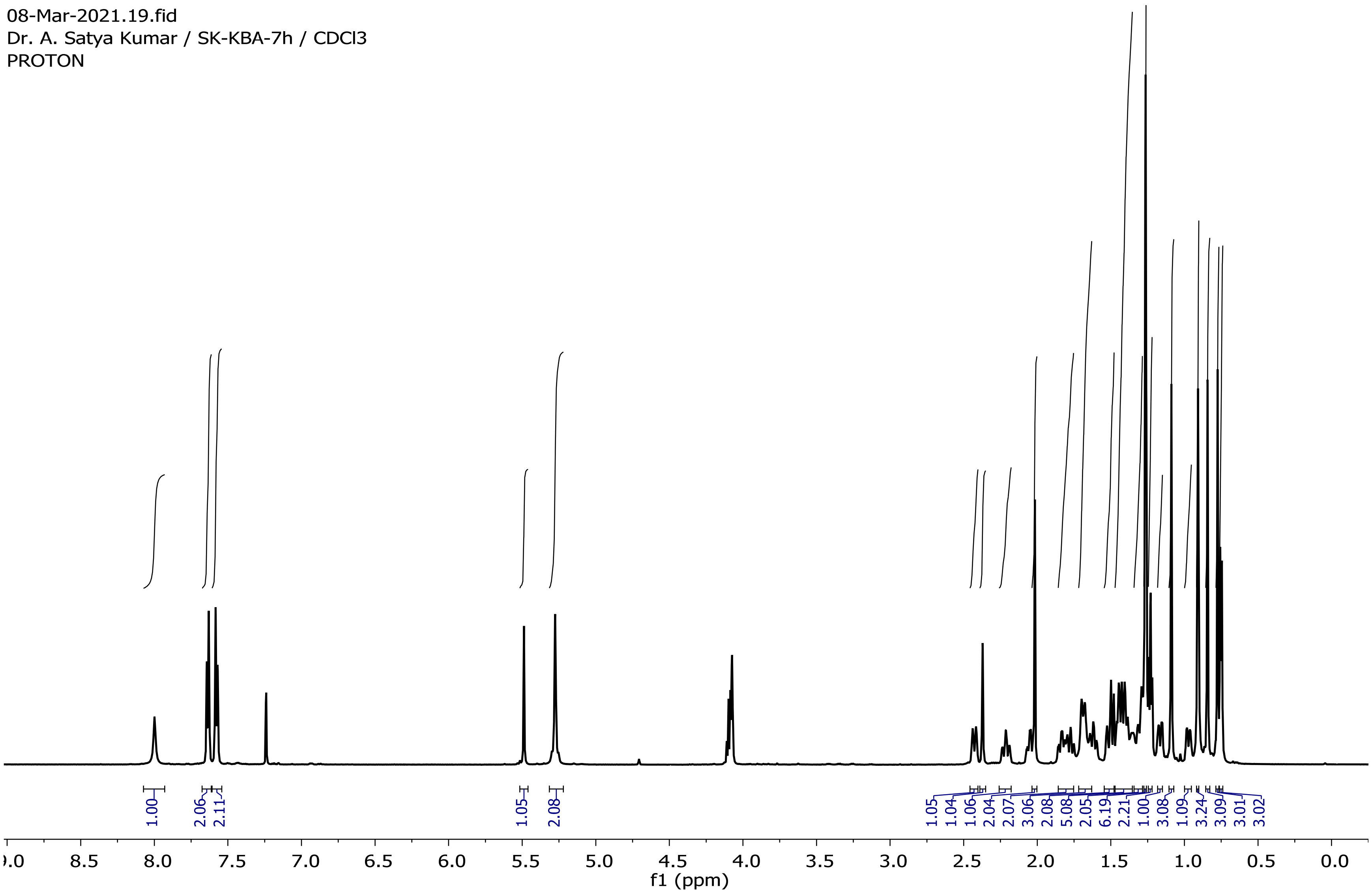

Figure S67: ^{1}H-NMR spectrum (600 MHz, $CDCl_3$) of compound **7h**

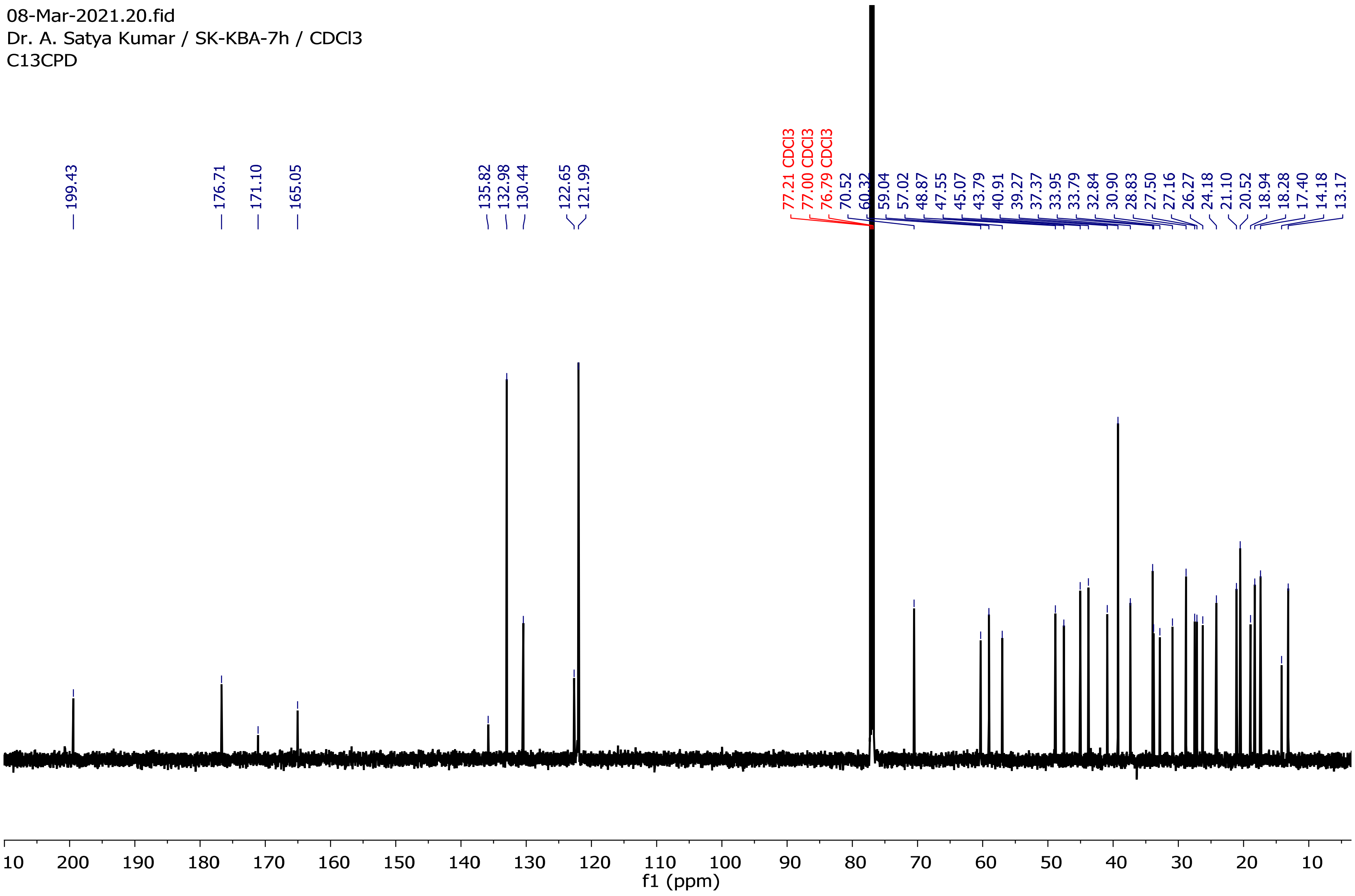

Figure S68: ^{13}C-NMR spectrum (150 MHz, $CDCl_3$) of compound **7h**

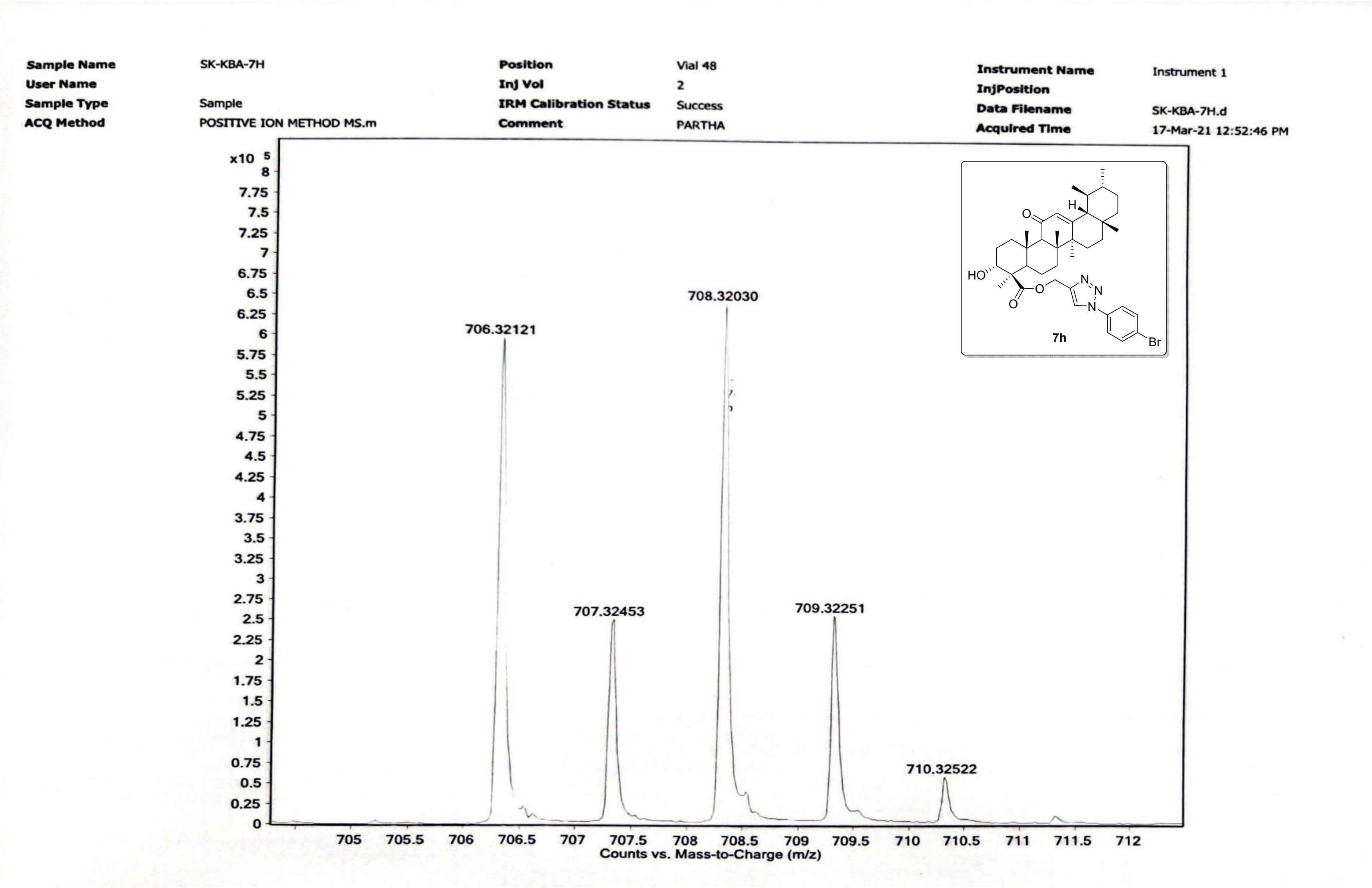

Figure S69: HRMS spectrum of compound **7h**

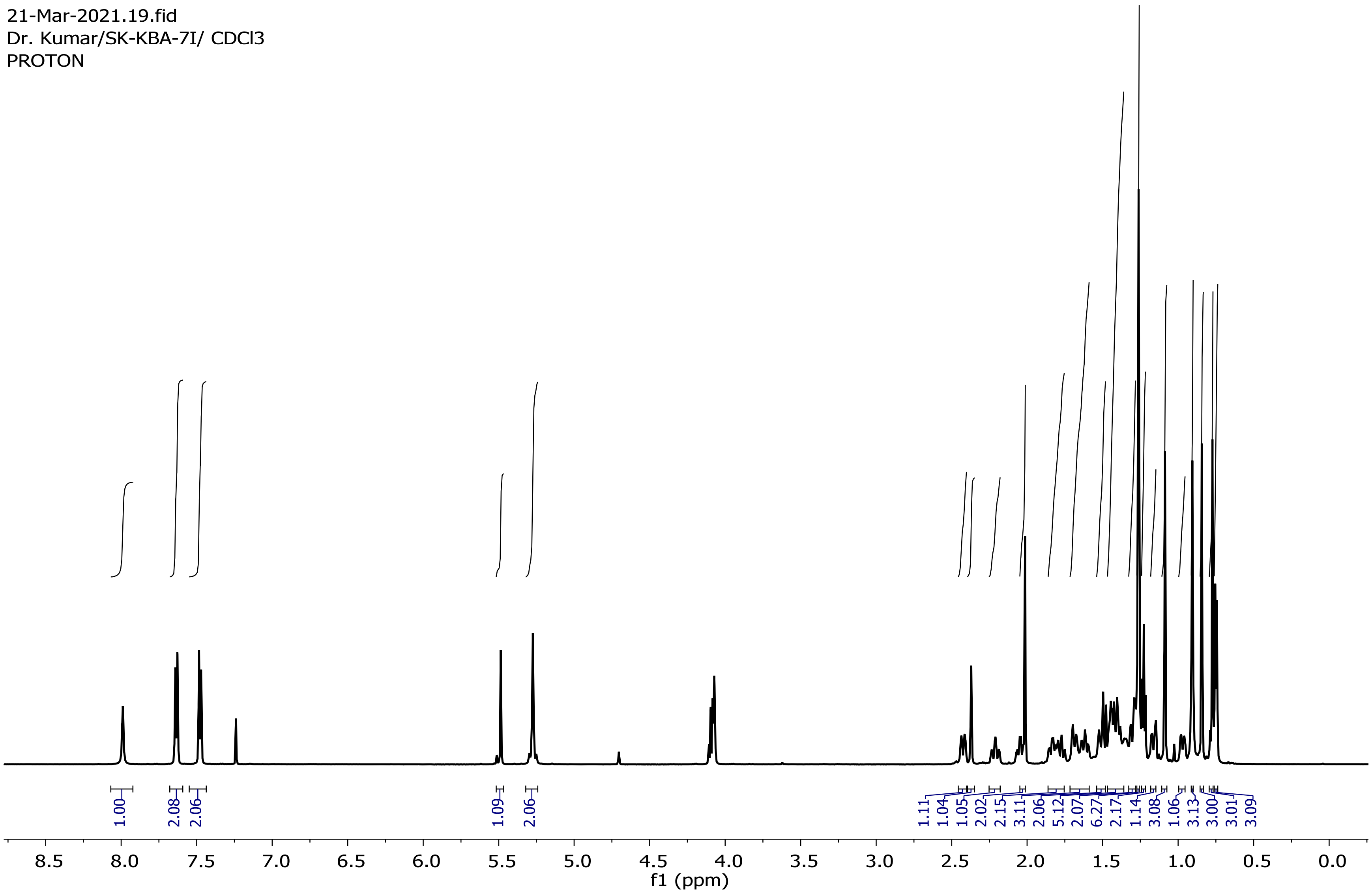

Figure S70: ^{1}H-NMR spectrum (600 MHz, $CDCl_3$) of compound **7i**

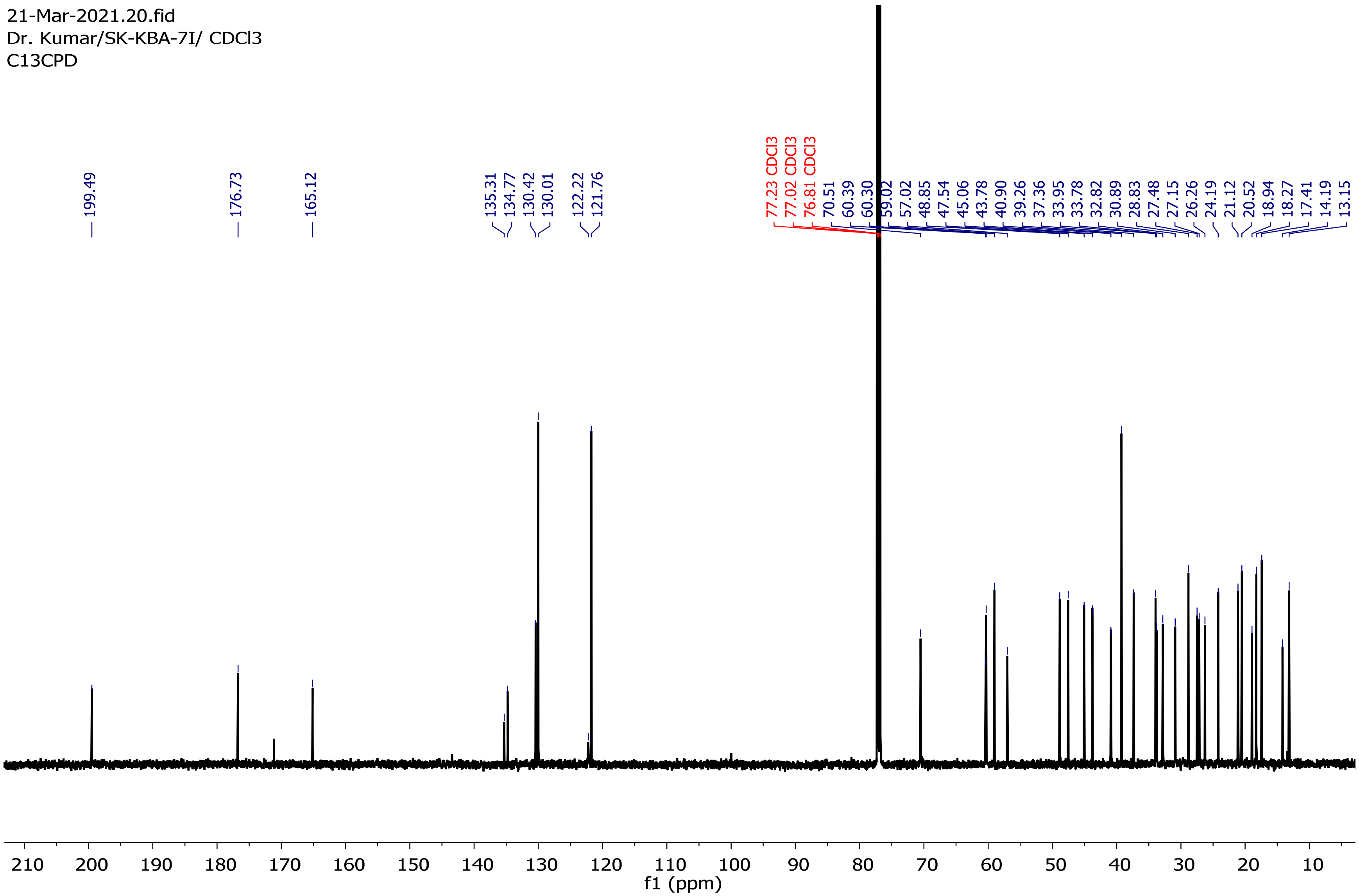

Figure S71: ^{13}C-NMR spectrum (150 MHz, $CDCl_3$) of compound **7i**

**Sample Name**	SK-KBA-7I	**Position**	Vial 34	**Instrument Name**	Instrument 1
**User Name**		**Inj Vol**	2	**InjPosition**	
**Sample Type**	Sample	**IRM Calibration Status**	Success	**Data Filename**	SK-KBA-7I.d
**ACQ Method**	POSITIVE ION METHOD MS.m	**Comment**		**Acquired Time**	22-Mar-21 2:56:25 PM

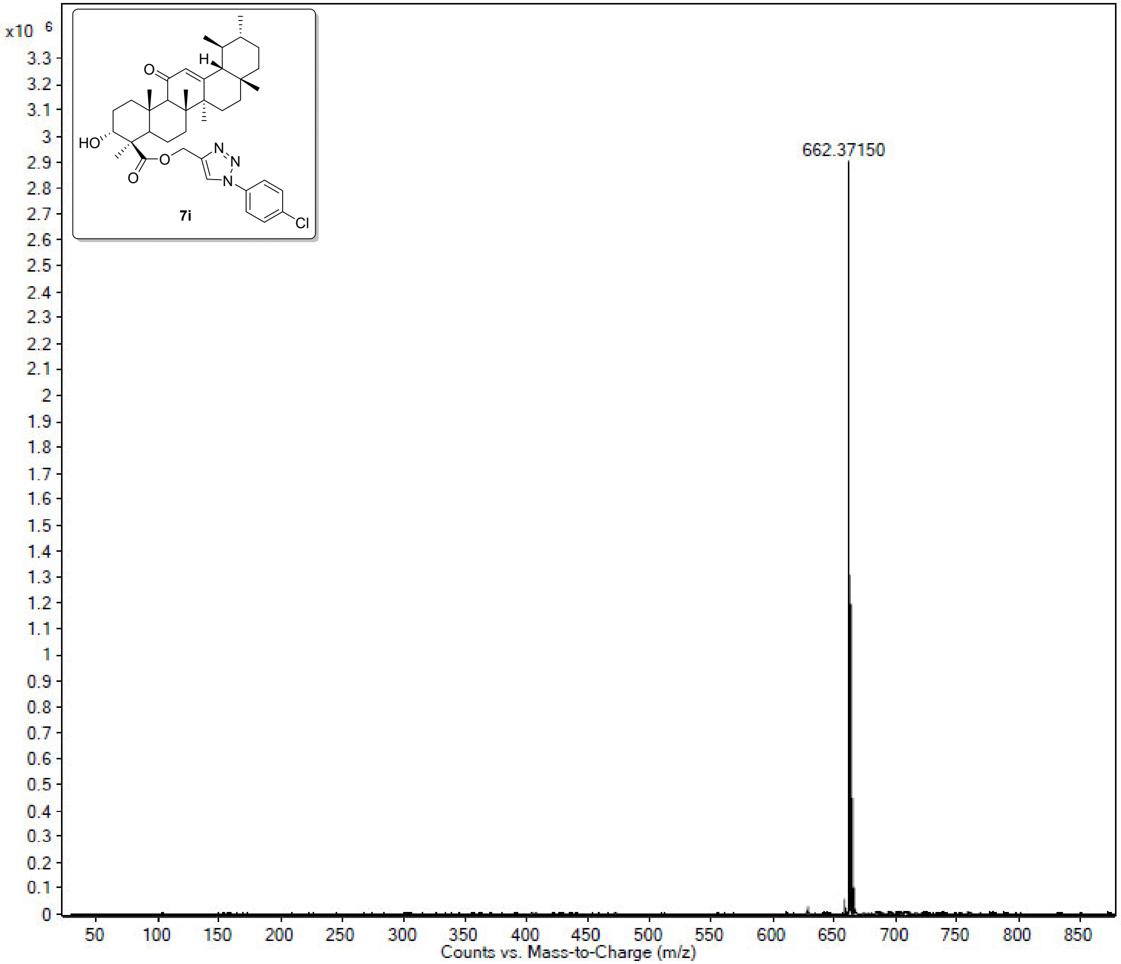

Figure S72: HRMS spectrum of compound **7i**

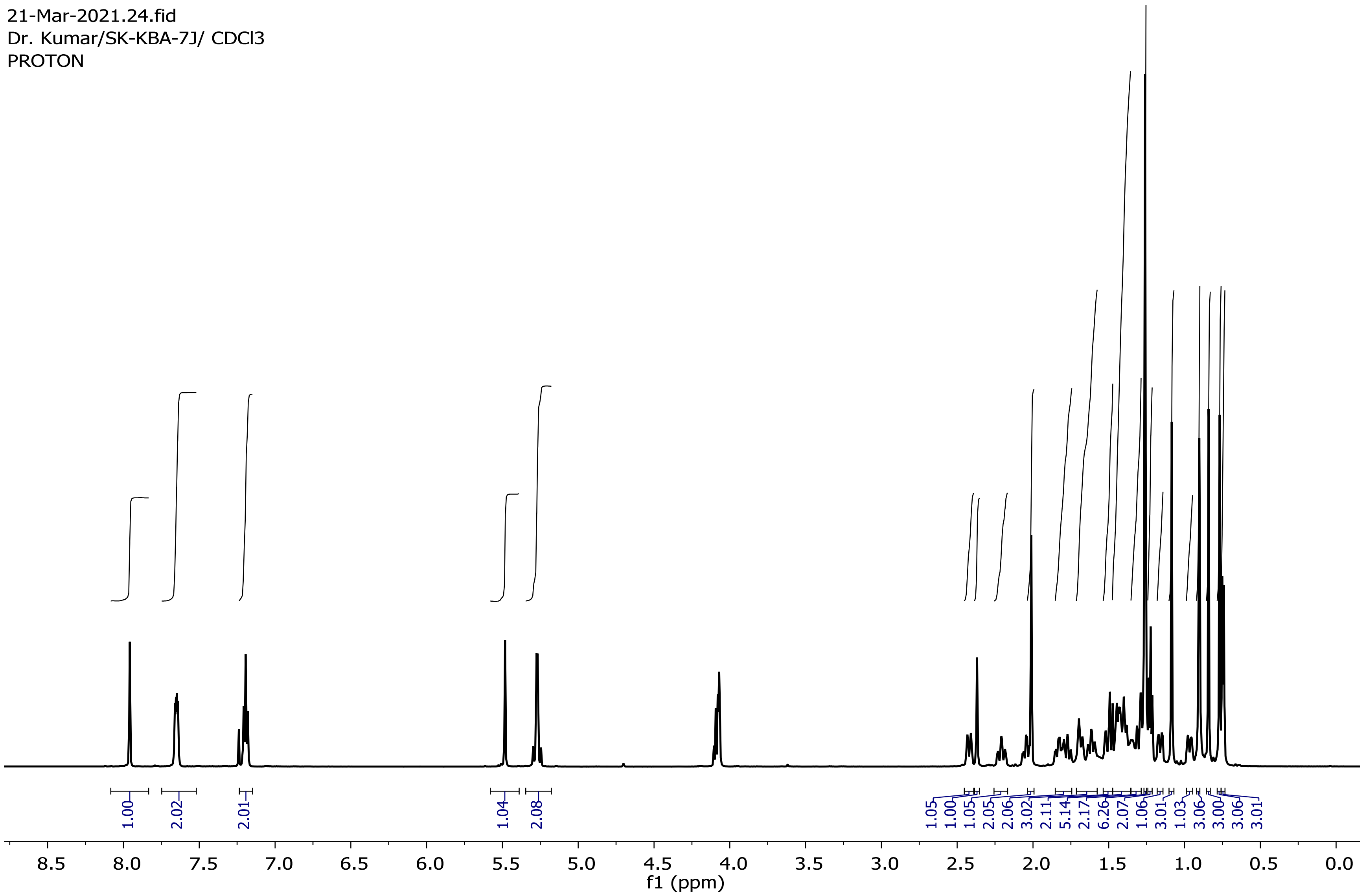

Figure S73: ^{1}H-NMR spectrum (600 MHz, $CDCl_3$) of compound **7j**

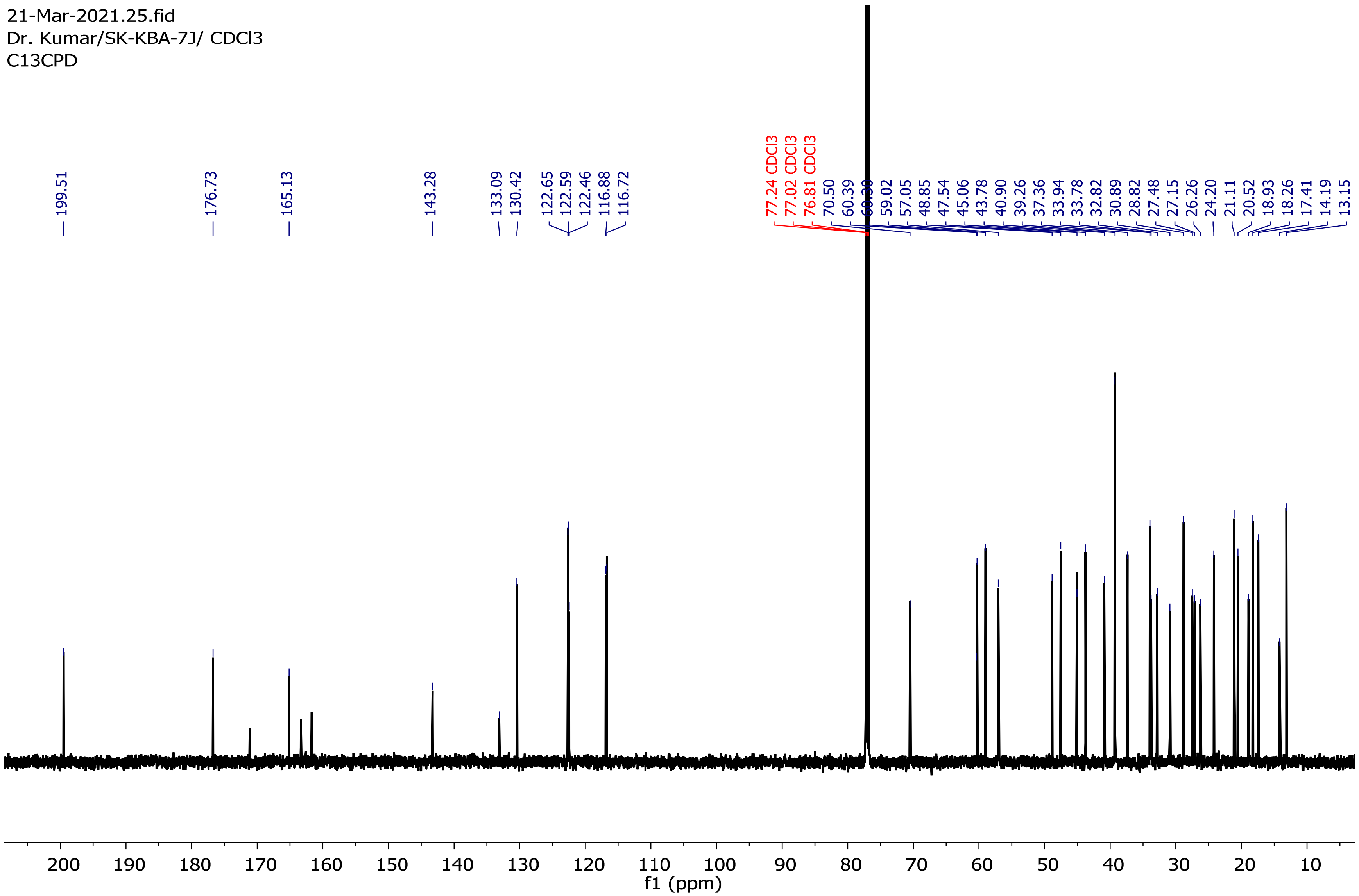

Figure S74: ^{13}C-NMR spectrum (150 MHz, $CDCl_3$) of compound **7j**

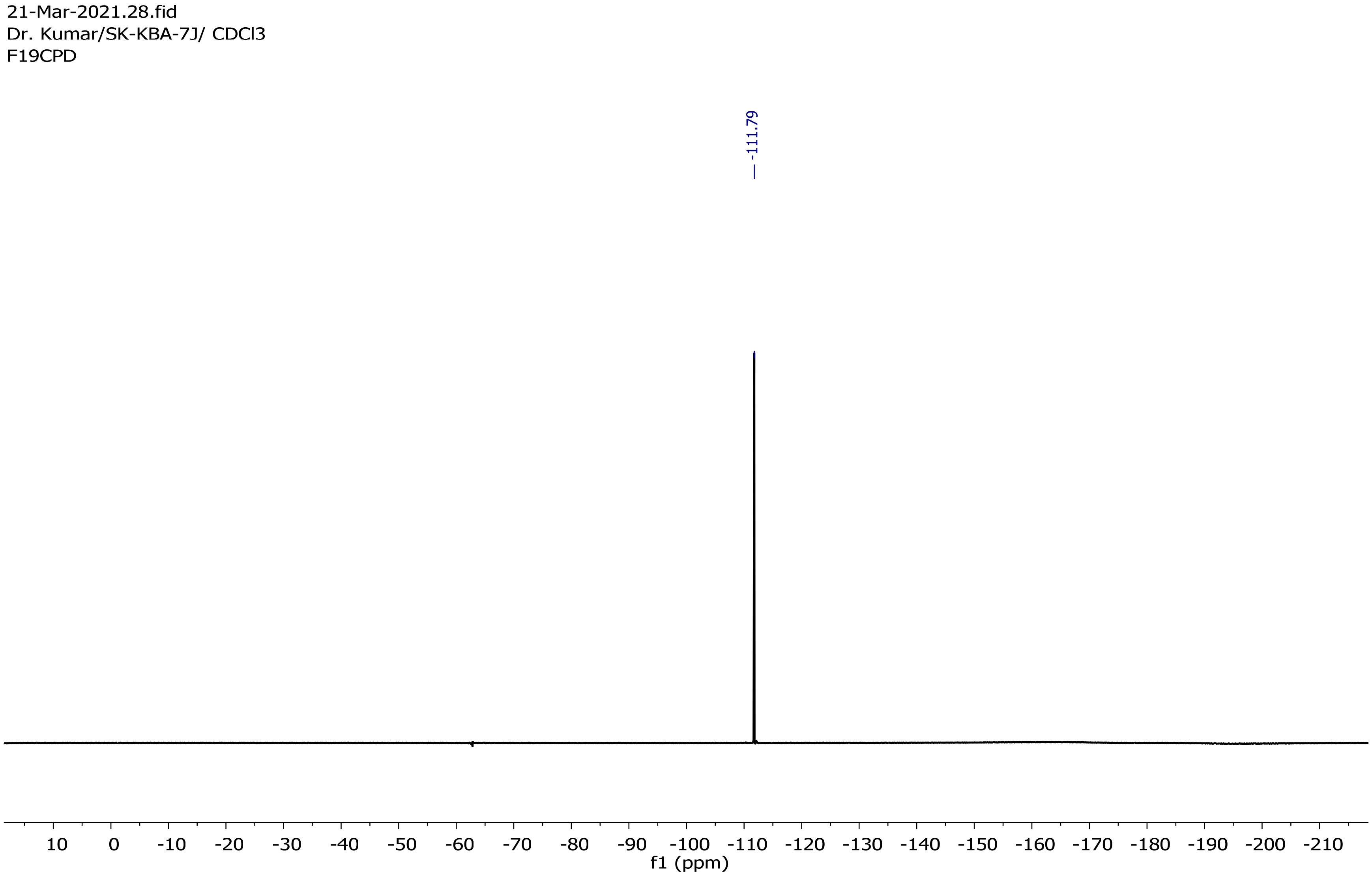

Figure S75: ^{19}F-NMR spectrum (564 MHz, $CDCl_3$) of compound **7j**

**Sample Name**	SK-KBA-7J	**Position**	Vial 35	**Instrument Name**	Instrument 1
**User Name**		**Inj Vol**	2	**InjPosition**	
**Sample Type**	Sample	**IRM Calibration Status**	Success	**Data Filename**	SK-KBA-7J.d
**ACQ Method**	POSITIVE ION METHOD MS.m	**Comment**		**Acquired Time**	22-Mar-21 3:02:06 PM

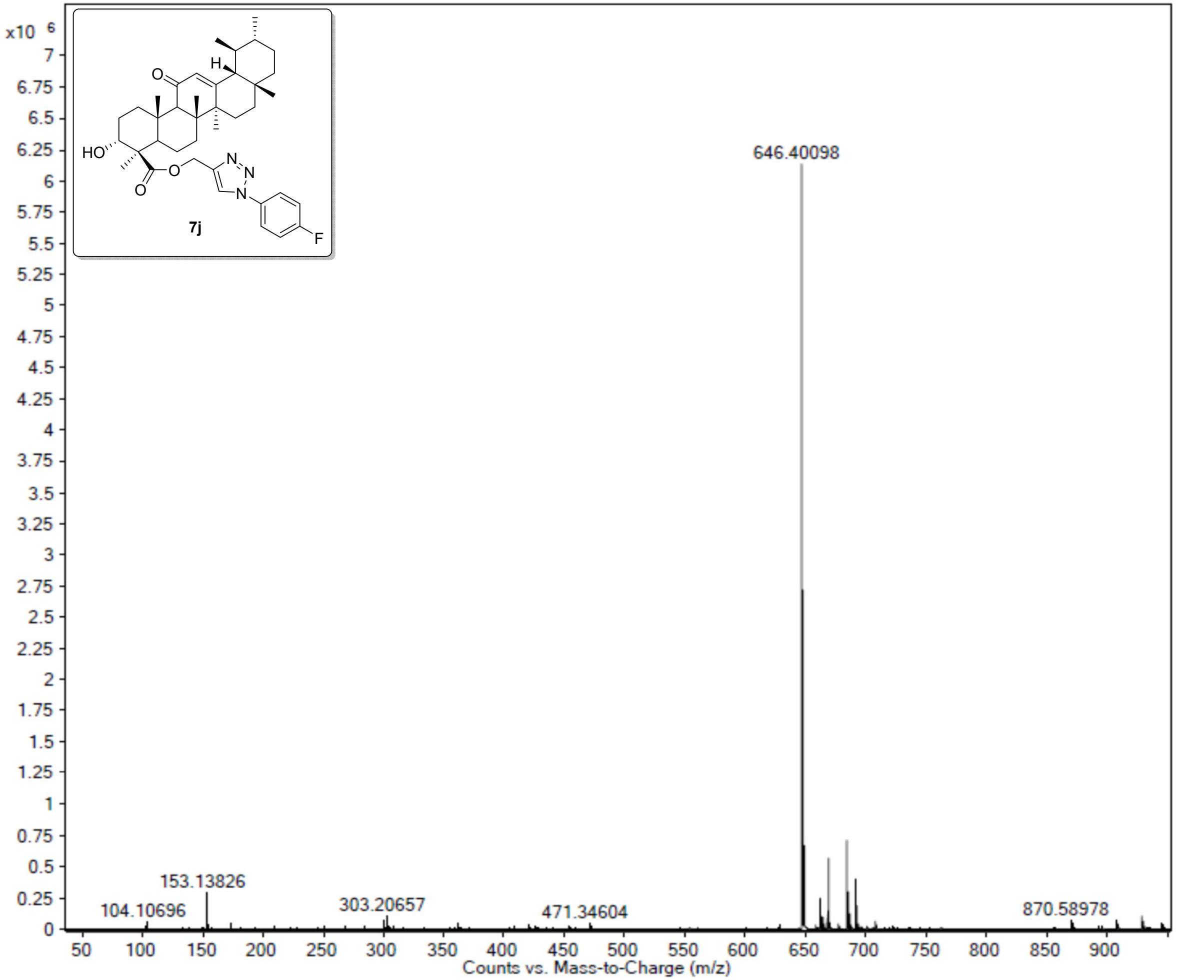

Figure S76: HRMS spectrum of compound **7j**

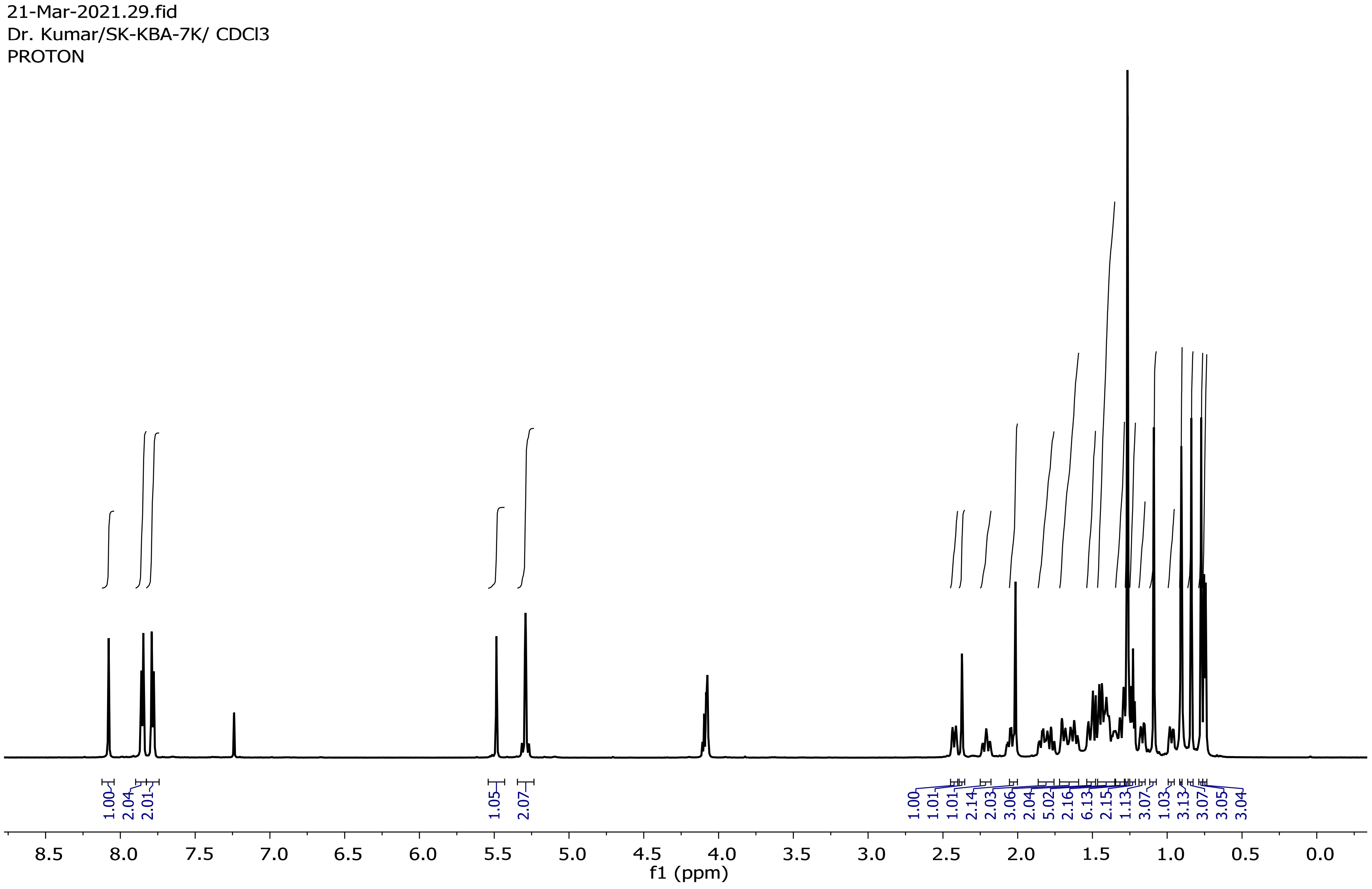

Figure S77: ^{1}H-NMR spectrum (600 MHz, $CDCl_3$) of compound **7k**

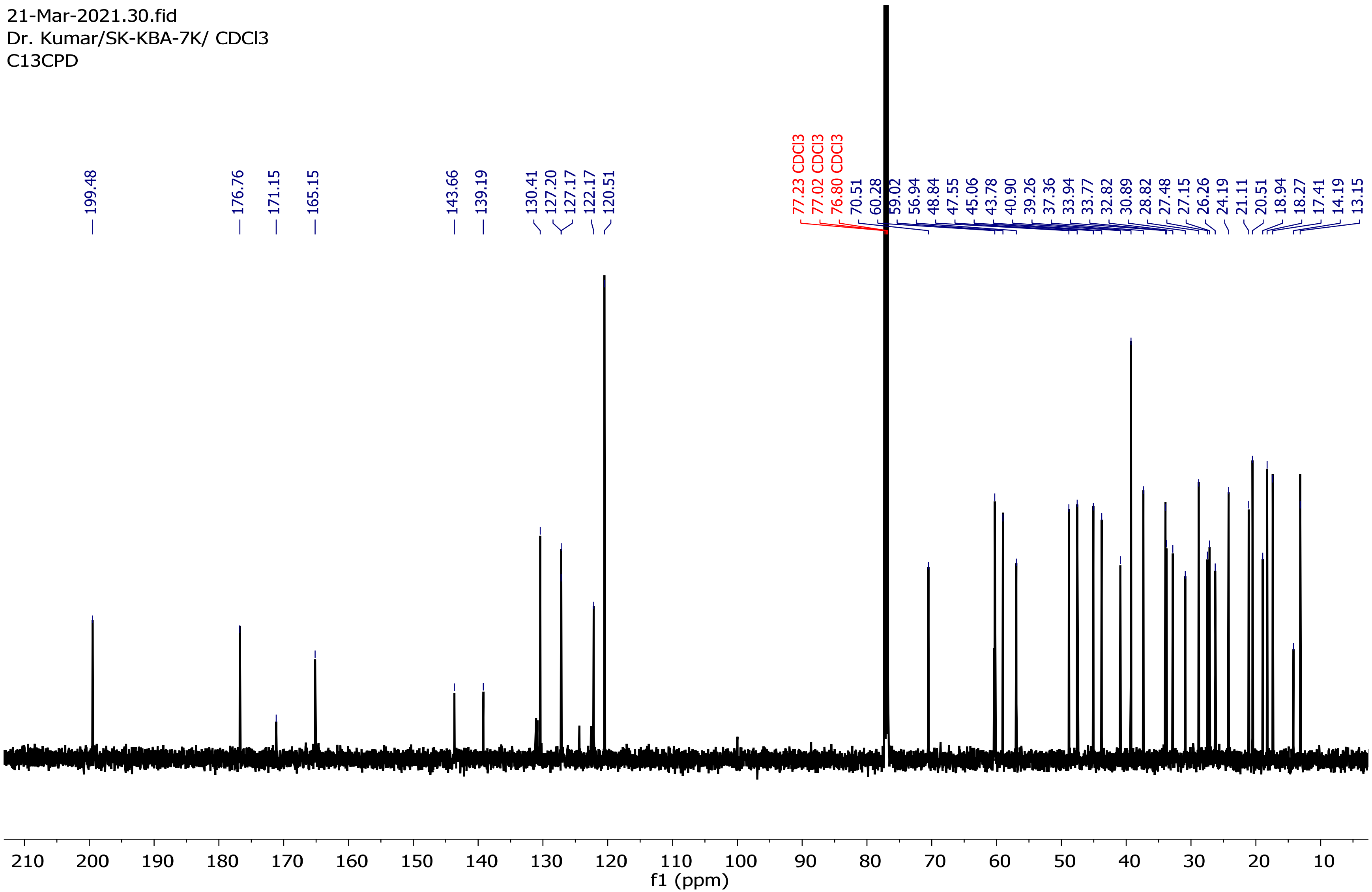

Figure S78: ^{13}C-NMR spectrum (150 MHz, $CDCl_3$) of compound **7k**

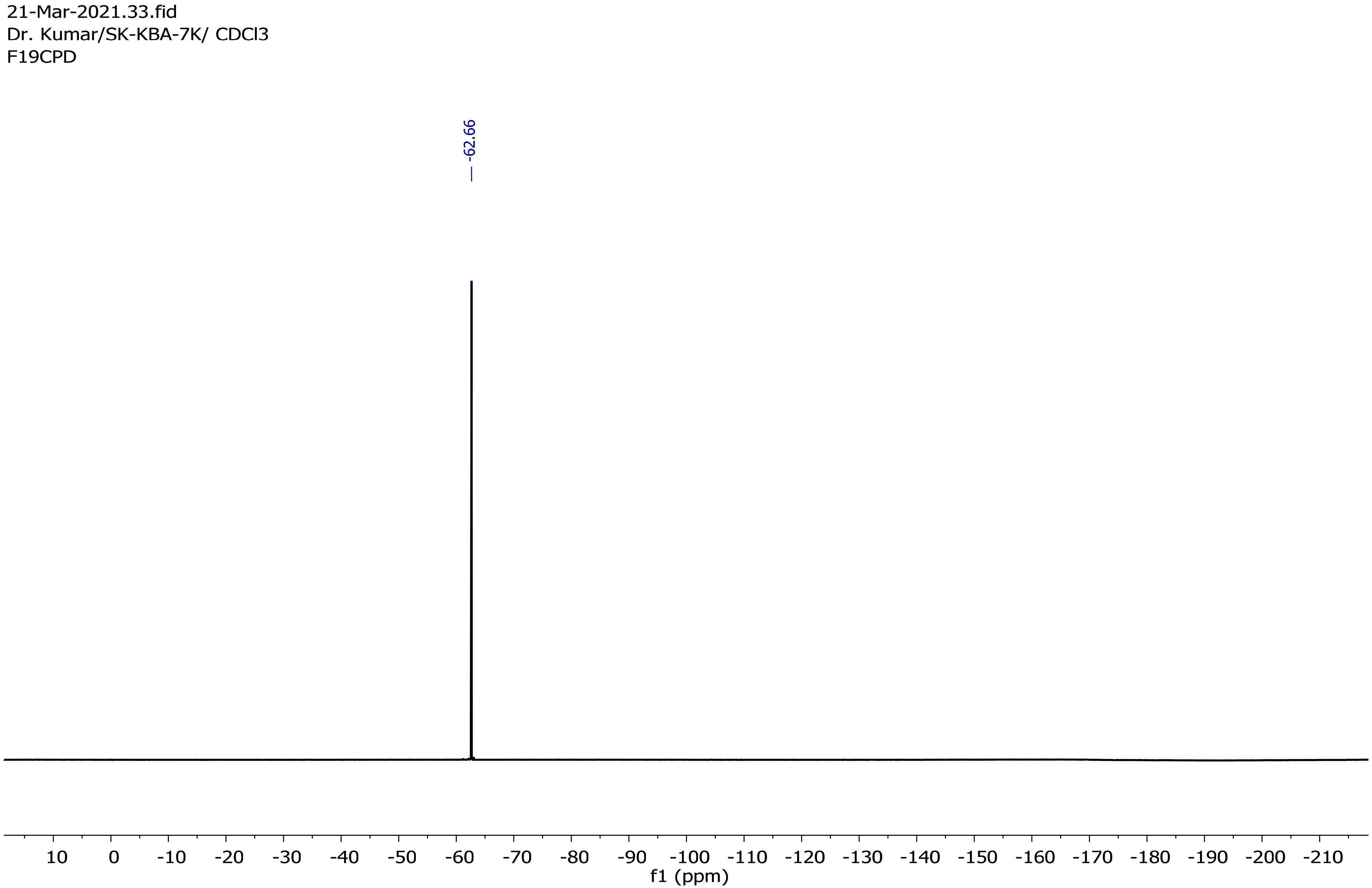

Figure S79: ^{19}F-NMR spectrum (564 MHz, $CDCl_3$) of compound **7k**

**Sample Name**	SK-KBA-7K	**Position**	Vial 36	**Instrument Name**	Instrument 1
**User Name**		**Inj Vol**	2	**InjPosition**	
**Sample Type**	Sample	**IRM Calibration Status**	Success	**Data Filename**	SK-KBA-7K.d
**ACQ Method**	POSITIVE ION METHOD MS.m	**Comment**		**Acquired Time**	22-Mar-21 3:07:40 PM

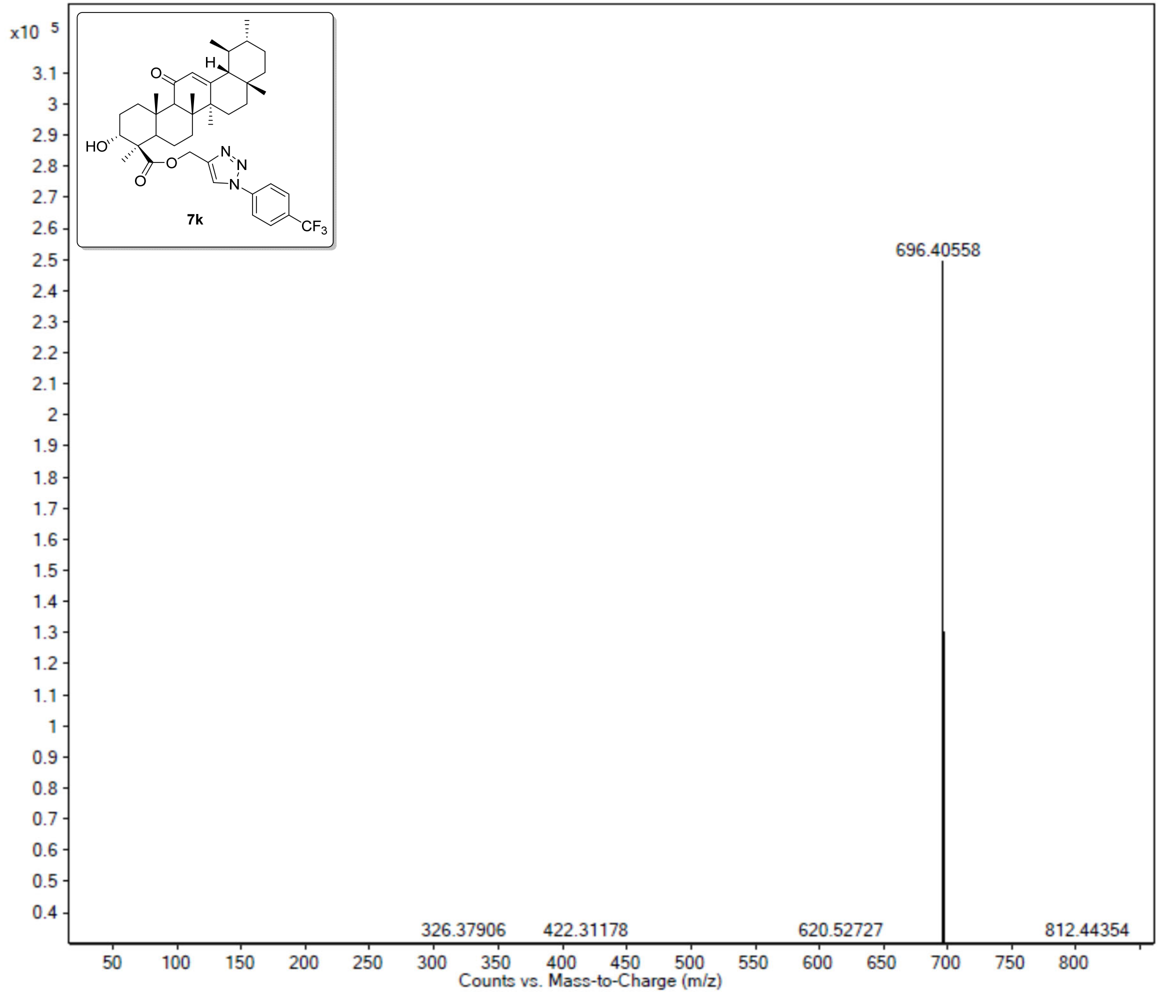

Figure S80: HRMS spectrum of compound **7k**

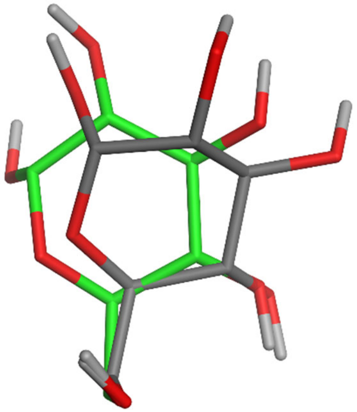

Figure S81. The re-docked conformation (green stick model) of maltose is superimposed on the X-ray conformation (grey stick model) present in the PDB code 3A4A

Table S1. The docking results of all the compounds.

Compounds	Score (Kcal/mol)	Ligand Atom	Receptor Atom	Interaction	Distance (Å)
**6f**	-9.18	O75	NE2-HIS280	HBA	2.00
		O97	N-ARG315	HBA	2.55
		O98	N-ARG315	HBA	2.44
		O86	OG-SER240	HBA	2.09
**7h**	-8.74	O58	O-SER157	HBD	2.59
		O77	N-ARG315	HBA	2.37
**6j**	-8.79	O86	OG-SER240	HBA	2.49
		O98	N-ARG315	HBA	2.26
		O99	N-ARG315	HBA	2.23
**6h**	-8.74	N84	NH1-ARG442	HBA	2.18
		O86	N-ARG315	HBA	1.83
**6g**	-8.67	O75	NE2-HIS280	HBA	1.95
		O86	OG-SER240	HBA	2.11
**6c**	-7.78	O76	NE2-HIS280	HBA	2.19
		O86	OG-SER240	HBA	2.48
		O104	NE2-GLN279	HBA	2.38
**6k**	-7.89	O75	NE2-HIS280	HBA	2.35
**7g**	-7.66	O58	OE2-GLU411	HBD	2.32

		O78	NE2-GLN279	HBA	2.07
**7k**	-7.04	O77	NE2-HIS280	HBA	2.13
		O78	NE2-HIS280	HBA	2.12
**6e**	-7.55	O75	NE2-HIS280	HBA	1.96
**6a**	-7.24	N84	NE2-HIS351	HBA	2.53
**7b**	-7.21	O58	NH1-ARG442	HBA	2.87
		6-ring	6-ring-TYR72	π-π	3.85
**7c**	-7.35	O58	OD2-ASP242	HBD	1.67
**6b**	-7.02	O76	NE2-HIS280	HBA	2.08
		O86	OG-SER240	HBA	2.44
**7a**	-7.11	N87	NE2-HIS280	HBA	2.27
		5-ring	CD2-PHE303	π-H	3.61
**6d**	-7.05	O75	NE2-GLN279	HBA	2.30
		F98	NE2-HIS351	HBA	1.86
**7e**	-7.45	O88	OG-SER240	HBA	2.13
**7f**	-7.53	N87	NE2-GLN279	HBA	3.09
		F102	NE2-HIS112	HBA	2.98
**7d**	-6.98	F100	NH2-ARG213	HBA	2.88
**7j**	-6.36	O58	OD1-ASP215	HBD	1.97
		O58	OD2-ASP215	HBD	2.37

		O88	NE2-GLN279	HBA	1.89
		O78	NE2-HIS351	HBA	2.20
		6-ring	6-ring-PHE301	π-π	3.29
**6i**	-6.45	O75	NH1-ARG442	HBA	2.21
		O99	ND2-ASN350	HBA	2.21
		N85	NE2-HIS351	HBA	2.40
		O86	NH1-ARG315	HBA	2.83
		5-ring	NE2-HIS351	π-H	3.48
**7i**	-6.59	O58	O-TYR158	HBD	2.78
		C11	5-ring-HIS280	π-H	4.09
		C10	5-ring-HIS280	π-H	3.93
		6-ring	6-ring-PHE303	π-π	3.54
**3**	-6.15	O75	NH1-ARG442	HBA	2.01
**4**	-6.20	O58	NH1-ARG442	HBA	2.13
**2** (KBA)	-4.44	O58	OD1-ASP215	HBD	2.73
		O78	NH1-ARG442	HBA	2.87
**1** (AKBA)	-3.23	O83	NH2-ARG213	HBA	1.69
		O84	NE2-HIS351	HBA	1.98

HBA = Hydrogen bond acceptor, HBD = Hydrogen bond donor.

Table S2. Crystal data of the compound **4.**

**Compound**	**4**
Chemical formula	$C_{33}H_{47}O_4$
Mr	507.70
Crystal system, space group	Orthorhombic, $P2_12_12_1$
Temperature (K)	296
a (Å)	13.834(5)
b (Å)	23.617(8)
c (Å)	8.757(3)
α (°)	90
β (°)	90
γ (°)	90
V (Å^3)	2861.0(17)
Z	4
μ (mm^{-1})	0.08
Absorption correction	Multi-scan, SADABS
T_{min}, T_{max}	0.6596, 0.7451
No. of measured, independent and observed [I > 2σ(I)] reflections	43500, 4914, 3977
R_{int}	0.0770
$(\sin θ/λ)_{max}$ (Å^{-1})	0.591
$R[F^2 > 2σ(F^2)]$, $wR(F^2)$, S	0.0418, 0.1056, 1.043
No. of reflections	4914
No. of parameters	344
No. of restraints	1
$Δρ_{max}$, $Δρ_{min}$ (eÅ^{-3})	0.164, -0.163
